# TNF-**α** impairs platelet function by inhibiting autophagy and disrupting metabolism via syntaxin 17 downregulation

**DOI:** 10.1172/JCI186065

**Published:** 2025-06-10

**Authors:** Guadalupe Rojas-Sanchez, Jorge Calzada-Martinez, Brandon McMahon, Aaron C. Petrey, Gabriela Dveksler, Gerardo P. Espino-Solis, Orlando Esparza, Giovanny Hernandez, Dennis Le, Eric P. Wartchow, Ken Jones, Lucas H. Ting, Catherine Jankowski, Marguerite R. Kelher, Marilyn Manco-Johnson, Marie L. Feser, Kevin D. Deane, Travis Nemkov, Angelo D’Alessandro, Andrew Thorburn, Paola Maycotte, José A. López, Pavel Davizon-Castillo

**Affiliations:** 1 Bloodworks Research Institute, Seattle, Washington, USA.; 2 Division of Hematology, School of Medicine, University of Colorado-Anschutz Medical Campus, Aurora, Colorado, USA.; 3 Department of Pathology, Division of Microbiology and Immunology, University of Utah, Salt Lake City, Utah, USA.; 4 Department of Pathology, Uniformed Services University of the Health Sciences, Bethesda, Maryland, USA.; 5 Laboratorio Nacional de Citometría de Flujo, Facultad de Medicina y Ciencias Biomédicas, Universidad Autónoma de Chihuahua, Circuito Universitario Campus Universitario II, Chihuahua, México.; 6 Department of Pediatrics Hematology/Oncology and Bone Marrow Transplantation, University of Colorado-Anschutz Medical Campus, Aurora, Colorado, USA.; 7 Electron Microscopy Laboratory, Children’s Hospital Colorado, Aurora, Colorado, USA.; 8 Bioinformatics Solutions, LLC, Sheridan, Wyoming, USA.; 9 Stasys Medical, Seattle, Washington, USA.; 10 University of Colorado College of Nursing,; 11 Department of Surgery, University of Colorado School of Medicine,; 12 Department of Pediatrics, Hemophilia and Thrombosis Center,; 13 Division of Rheumatology,; 14 Biochemistry and Molecular Genetics Department, and; 15 Department of Pharmacology, University of Colorado-Anschutz Medical Campus, Aurora, Colorado, USA.; 16 Centro de Investigación Biomédica de Oriente, Instituto Mexicano del Seguro Social, Puebla, México.; 17 Division of Hematology and Oncology, and; 18 Department of Pediatrics, Hematology/Oncology, University of Washington, Seattle, Washington, USA.; 19 Seattle Children’s Cancer and Blood Disorders Center, Seattle, Washington, USA.

**Keywords:** Hematology, Metabolism, Autophagy, Mitochondria, Platelets

## Abstract

Platelets play a dual role in hemostasis and inflammation-associated thrombosis and hemorrhage. Although the mechanisms linking inflammation to platelet dysfunction remain poorly understood, our previous work demonstrated that TNF-α alters mitochondrial mass, platelet activation, and autophagy-related pathways in megakaryocytes. Here, we hypothesized that TNF-α impairs platelet function by disrupting autophagy, a process critical for mitochondrial health and cellular metabolism. Using human and murine models of TNF-α–driven diseases, including myeloproliferative neoplasms and rheumatoid arthritis, we found that TNF-α downregulates syntaxin 17 (STX17), a key mediator of autophagosome-lysosome fusion. This disruption inhibited autophagy, leading to the accumulation of dysfunctional mitochondria and reduced mitochondrial respiration. These metabolic alterations compromised platelet-driven clot contraction, a process linked to thrombotic and hemorrhagic complications. Our findings reveal a mechanism by which TNF-α disrupts hemostasis through autophagy inhibition, highlighting TNF-α as a critical regulator of platelet metabolism and function. This study provides potentially new insights into inflammation-associated pathologies and suggests autophagy-targeting strategies as potential therapeutic avenues to restore hemostatic balance.

## Abstract 2

TNFα reduces STX17 in platelets, inhibiting autophagy, disrupting mitochondrial function, and impairing clot contraction, highlighting autophagy-targeting therapies to counteract inflammation-driven thrombosis and hemorrhage.

## Graphical abstract

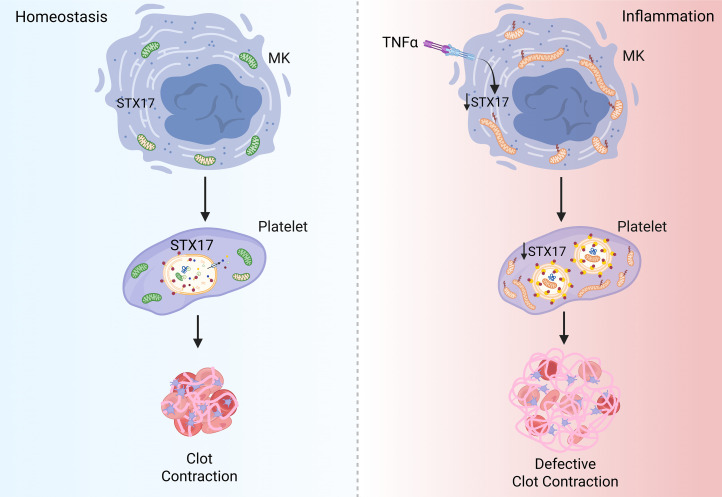

## Introduction

Despite their small size and lack of a nucleus, platelets rely on mitochondria to fulfill their primary hemostatic function. Although platelets contain relatively few mitochondria, these organelles provide approximately 35% of their basal energy demands (1). Upon platelet activation, mitochondrial respiration increases to meet the elevated energy requirements necessary for effective clot formation and contraction (2–6). Therefore, conditions that alter mitochondrial function, such as changes in mitochondrial fusion, fission, or clearance, can potentially disrupt platelet hemostatic function (7–9).

Autophagy is an essential catabolic process that eliminates damaged or long-lived proteins, lipids, nucleic acids, and organelles (10). The byproducts of autophagy help sustain cellular functions and support mitochondrial metabolism (11–17). Mitophagy, a subtype of autophagy, targets damaged mitochondria for degradation to preserve mitochondrial fitness and to support optimal energy production (18–20). Consequently, defects in autophagy and mitophagy can disrupt cellular metabolism and cause reduced ATP production and the accumulation of dysfunctional mitochondria. Because of the critical role of autophagy and mitophagy in maintaining mitochondrial function, disruptions in these processes are implicated in various pathological states (21–23).

Recent studies have demonstrated that both human and murine platelets express autophagy-related (ATG) proteins and engage in active basal autophagy and mitophagy (24–29). Defects in platelet autophagic pathways are associated with various inflammatory conditions including sepsis and diabetes (28, 30). Notably, these conditions result in consistently elevated plasma levels of TNF-α and a disrupted hemostatic balance marked by increased thrombosis or hemorrhage (31, 32). Our previous work established a link between TNF-α and the generation of platelets with increased mitochondrial mass and reactivity, suggesting the pivotal role of TNF-α in platelet metabolism and function (33). Owing to the anucleate nature and limited protein synthesis capacity of platelets, the observed increase in mitochondrial mass is unlikely to result from mitochondrial biogenesis. Furthermore, transcriptome analysis of megakaryocytes (MKs) from mice with elevated TNF-α levels suggested that impaired autophagy may drive these observed changes. Given that autophagy plays a crucial role in maintaining mitochondrial mass, health, and energy homeostasis, we hypothesized that TNF-α–driven autophagic disruption in platelets may drive these changes.

This study reveals a mechanism by which TNF-α inhibits autophagy and mitophagy in platelets and MKs through the downregulation of syntaxin 17 (STX17). This SNARE protein is essential for the fusion of autophagosomes with lysosomes, a critical step in both processes (34–36). The downregulation of STX17 resulted in the accumulation of dysfunctional mitochondria, impaired mitochondrial respiration, and decreased cellular energy production. These metabolic deficits compromised platelet function, leading to impaired clot contraction. Notably, TNF-α blockade in a mouse model of aseptic inflammation prevented STX17 downregulation in platelets. This intervention preserved autophagic pathways, maintained mitochondrial function, and sustained clot contraction. These findings underscore the critical role of STX17 in maintaining platelet metabolism and function under inflammatory conditions.

Collectively, our findings demonstrated that TNF-α disrupts the hemostatic balance by downregulating STX17, impairing autophagic pathways, and altering the metabolism of MKs and platelets. Our research underscores the essential role of autophagy in platelet function. Targeting TNF-α signaling or enhancing autophagy could be potential therapeutic strategies to restore platelet function and hemostatic balance under inflammatory conditions.

## Results

### JAK2 V617F MPN platelets exhibit a hypometabolic state and defective clot contraction.

Our research revealed a link between elevated TNF-α levels and increased mitochondrial mass in platelets from individuals with JAK2 V617F polycythemia vera (PV) (33). PV is a subtype of BCR/ABL1-negative myeloproliferative neoplasms (MPNs). In PV (hereafter referred to as MPN), the JAK2 V617F mutation drives excessive erythrocyte production and induces TNF-α release by mononuclear myeloid cells (37–39). Thrombotic and hemorrhagic complications are the primary causes of death and disability in patients with MPNs (40–43). These characteristics make MPNs an ideal model for studying the functional and metabolic characteristics of platelets in TNF-α–driven conditions associated with hemostatic imbalance.

**Figure 1:**
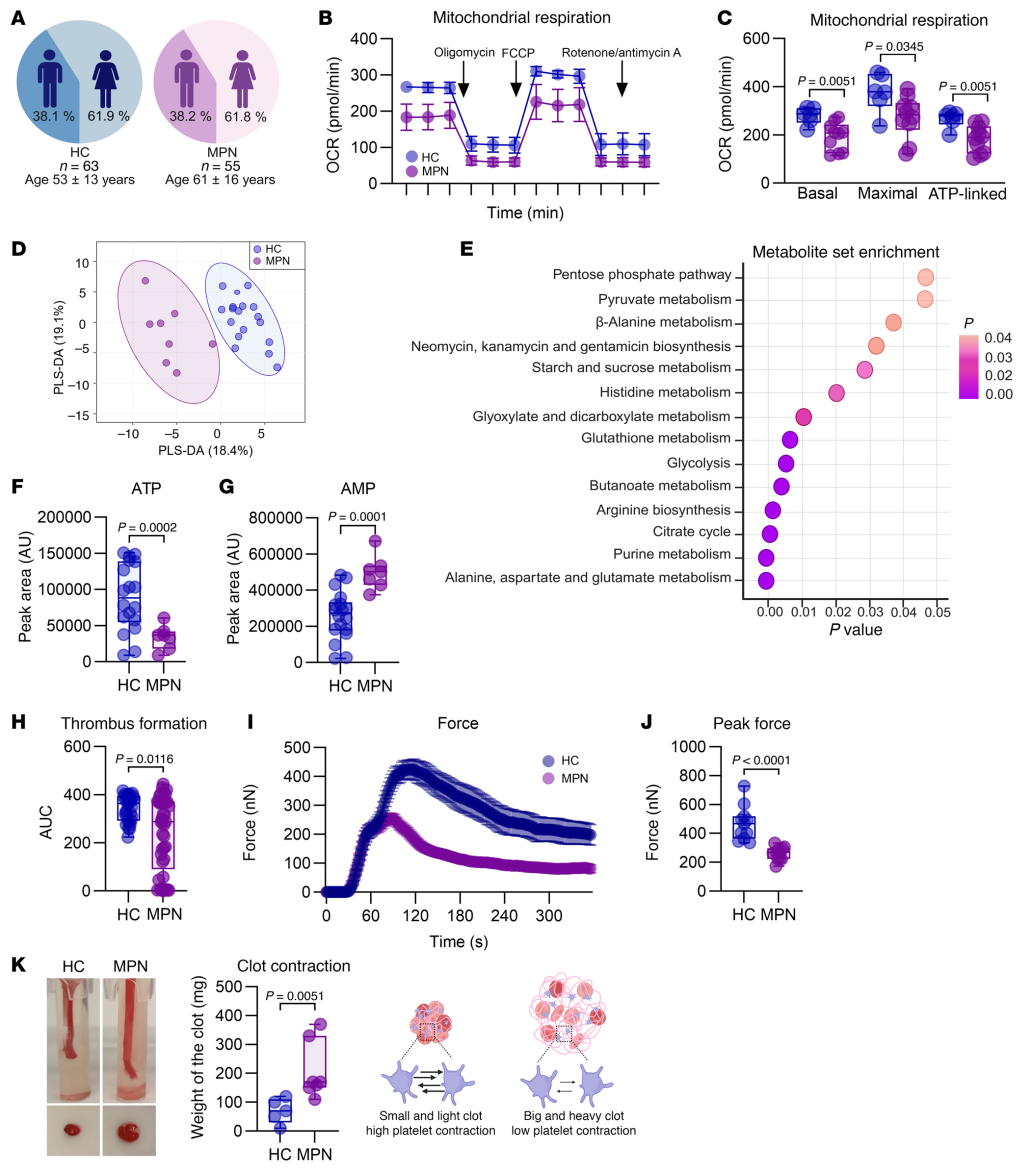
Metabolic and functional characterization of MPN platelets. (**A**) Demographics of volunteers providing healthy controls (HCs) and MPN platelets (JAK2 V617F polycythemia vera). (**B**) The OCRs of washed platelets from HCs (*n* = 6) and patients with MPN (*n* = 10) were measured using a Seahorse XFe24 analyzer (mean ± SEM). (**C**) Basal, maximal, and ATP-linked respiration parameters (*n* = 6 HCs, *n* =10 patients with MPN); unpaired *t* test. (**D**) PLS-DA of metabolome profiles (*n* = 18 HCs, *n* = 8 patients with MPN) was performed using UHPLC-MS. (**E**) Metabolite enrichment of the top 30 significant hits (see Supplemental Figure 1D). (**F**) ATP and (**G**) AMP levels (*n* = 6 HCs, *n* = 10 MPNs); unpaired *t* test with Welch’s correction. (**H**) Thrombus formation assay in whole blood using the T-TAS analyzer and PL chips (*n* = 30 HCs, *n* = 41 patients with MPN); Mann-Whitney *U* test. (**I**) Platelet strength assay and (**J**) peak force (*n* = 11 each); unpaired *t* test. (**K**) Thrombin-induced clot contraction assay (*n* = 5 HCs, *n* = 7 MPNs); Mann-Whitney *U* test. Box plots (**C**, **F**, **G**, **H**, **J**, and **K**) represent the data distribution.

We studied 63 age- and sex-matched healthy controls (HCs) and 55 patients with MPN (Figure 1A and Supplemental Table 1; supplemental material available online with this article; https://doi.org/10.1172/JCI186065DS1). Most patients (87.3%) in the MPN group received standard therapies including at least one medication, such as aspirin, anticoagulants, hydroxyurea, JAK2 inhibitors, or IFN-α (Supplemental Table 1). At the time of sample collection, the hematocrit levels of these patients were at or below the cytoreductive treatment goal of 45% (Supplemental Figure 1A) (44, 45).

Given the increased mitochondrial mass in MPN platelets, we analyzed their bioenergetic profiles using Seahorse analysis. Surprisingly, mitochondrial respiration was significantly lower than that in HC platelets (Figure 1B), as indicated by decreased basal, maximal, and ATP-linked oxygen consumption rates (Figure 1C). These changes were not caused by diminished levels of key electron transport chain proteins, as MPN platelets presented increased levels of SDHB, UQCRC2, and COXII, which are components of complexes II, III, and IV, respectively (Supplemental Figure 1B). The decreased mitochondrial respiration was also not attributable to aspirin or other medications (Supplemental Figure 1C). Additionally, MPN platelets exhibited mitochondrial dysfunction, including mitochondrial depolarization, elevated cytoplasmic calcium levels, and increased ROS production (Supplemental Figure 1, D–F).

We subsequently conducted an unbiased semiquantitative metabolomic analysis using UHPLC-MS to identify metabolites that are differentially regulated in MPN platelets compared with age- and sex-matched HC platelets. By performing partial least squares discriminant analysis (PLS-DA), we identified cluster separation of the MPN and HC groups across principal component 1 (PC1), which explained 18.4% of the variance (Figure 1D). A heatmap displaying the top 30 significantly altered metabolites (*P* < 0.05) between HC and MPN platelets was created (Supplemental Figure 1G). Metabolite set enrichment analysis (MSEA) was performed to identify the functional pathways associated with these metabolites (Figure 1E). This analysis revealed significant enrichment in key mitochondrial pathways, including amino acid metabolism (e.g., alanine, aspartate, glutamate, and histidine), the TCA cycle, glycolysis, the pentose phosphate pathway, and fatty acid–related pathways such as glyoxylate, dicarboxylate, and butanoate metabolism. We observed a global decrease in purine metabolism, including decreased levels of ATP, ADP, GTP, and GDP (Figure 1, E and F, and Supplemental Figure 1G), alongside a concomitant elevation in AMP levels (Figure 1G). Overall, these data indicate that MPN platelets are in a hypometabolic state, marked by impaired mitochondrial respiration and reduced ATP production.

Next, we sought to identify the functional differences linked to the metabolic deficits of MPN platelets. Platelet adhesion and clot formation under flow conditions were assessed via a T-TAS analyzer in an assay designed to evaluate primary hemostasis. Whole blood treated with the anticoagulant benzylsulfonyl-D-Arg-Pro-4-amidinobenzylamide (BAPA) was perfused over a collagen-coated surface at an arterial shear rate of 1,500/s. These conditions mimic vessel injury. Compared to HC platelet samples, MPN platelets demonstrated significantly reduced adhesion and a lower AUC (Figure 1H). These findings demonstrate impaired adhesion and aggregation of MPN platelets under physiological shear conditions.

To determine whether the low metabolic activity of MPN platelets affects their contractile forces, we measured clot contraction using 2 methods: the platelet strength assay and the thrombin-induced clot contraction test. The platelet strength assay (Figure 1I and Supplemental Videos 1 and 2) is used to evaluate the first 10 minutes of clot contraction. As platelets contract within a clot, sensors measure the force exerted (46). Although MPN platelets achieved peak contractile force more rapidly than HC platelets did (Supplemental Figure 1H), the magnitude of the peak contractile force of MPN platelets was significantly lower (Figure 1J). Additionally, thrombi in the MPN group were unstable, as evidenced by an increased percentage of clot lysis (Supplemental Figure 1I). Hematocrit, platelet count, and age did not affect the observed functional differences between MPN and HC platelets (Supplemental Figure 1J). Similarly, in the thrombin-induced clot contraction assay, MPN platelets exhibited significantly reduced contraction. This led to the formation of larger and heavier clots because of incomplete serum extrusion (Figure 1K). Overall, these findings reveal a potential link between the metabolic deficits in MPN platelets and their impaired contractile forces. These functional differences are not attributable to variations in platelet size or granule content. The mean platelet volume was similar between MPN and HC platelets (Supplemental Figure 1A), and no significant differences were observed in dense granules or key α-granule proteins. Consistent with a previous report, resting levels of exposed P-selectin were elevated in MPNs (47). However, after thrombin stimulation, P-selectin levels were comparable between groups (Supplemental Figure 1, K–M).

### Autophagy and mitophagy are inhibited in MPN platelets.

**Figure 2:**
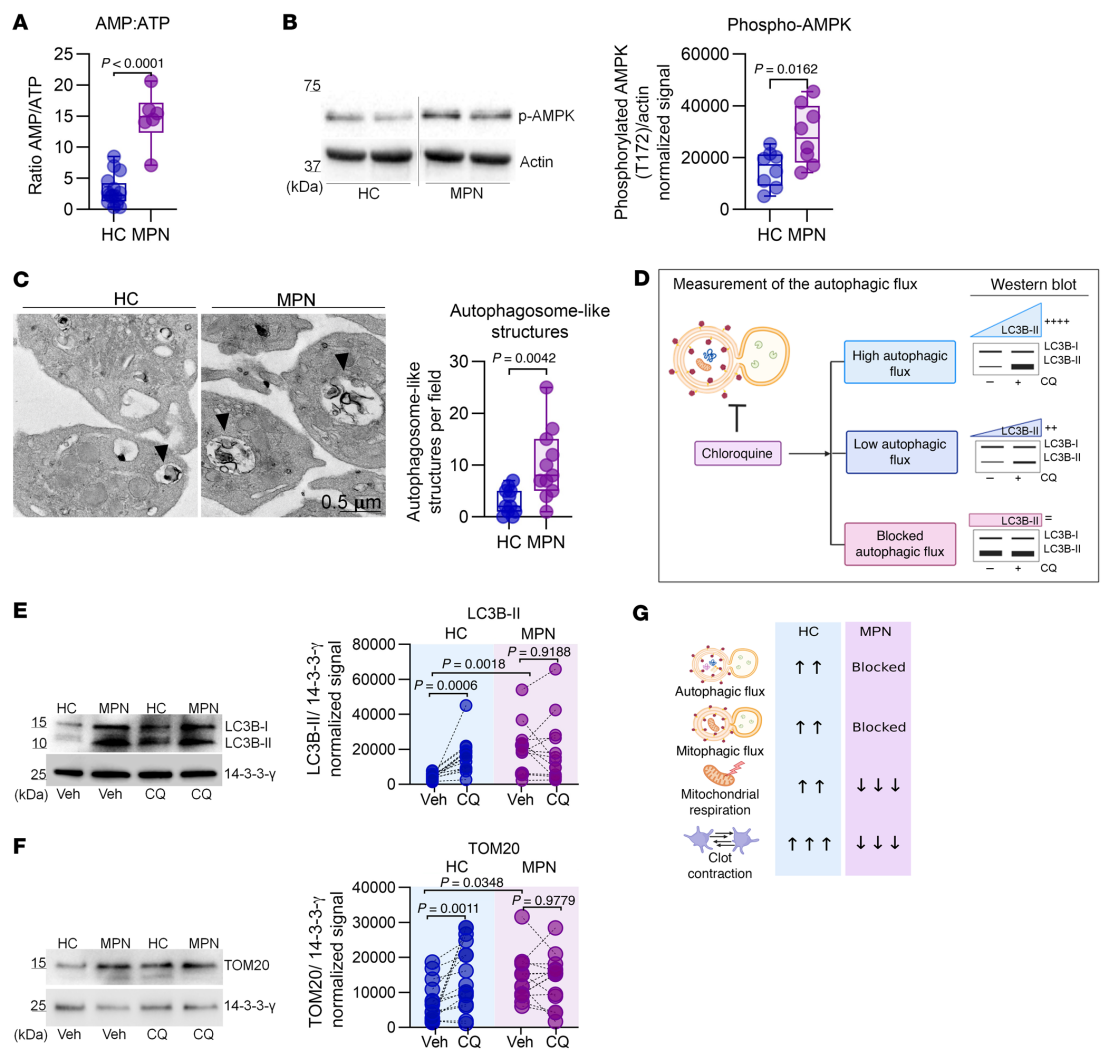
Assessment of autophagy pathways in MPN platelets. (**A**) AMP/ATP ratios derived from identified metabolites (Supplemental Figure 1D); unpaired *t* test. (**B**) Immunoblot of phosphorylated AMPK (T172) in platelets from HCs (*n* = 8) and patients with MPN (*n* = 8); unpaired *t* test. Noncontiguous gel lanes are indicated. (**C**) Transmission electron microscopy (TEM) analysis of pooled HC and pooled MPN platelets (*n* = 5 each). Representative images showing autophagosome-like structures, unpaired *t* test. Scale bar: 0.5 μm. (**D**) Guide for interpreting autophagic flux by immunoblotting. Platelets from HCs (*n* = 14) and patients with MPN (*n* = 14) were incubated for 2 hours with vehicle (Veh, PBS) or CQ (50 μM). Immunoblots of (**E**) LC3B-II and (**F**) TOM20. The graphs show their levels before and after CQ treatment; 2-way ANOVA with Šídák’s test. (**G**) Summary of the differences in autophagic flux status, mitochondrial respiration, and platelet clot contraction differences between HCs and patients with MPN. Box-and-whisker plots (**A**–**C**) represent the data distribution.

To test whether defective platelet autophagy is linked to inflammatory conditions with elevated TNF-α, we measured the autophagic and mitophagic flux in HC and MPN platelets. First, we calculated the AMP/ATP ratio and assessed the phosphorylation status of AMPK, a critical cellular energy sensor activated under low-energy conditions (48, 49). The AMP/ATP ratio, calculated from the metabolome dataset, was significantly higher in MPN platelets (Figure 2A). Consistent with these findings, phosphorylated AMPK levels were markedly increased in MPN samples (Figure 2B). These data confirmed the hypometabolic state of MPN platelets.

In line with previous reports (24, 25), we confirmed that platelets possess the machinery for autophagy and mitophagy. Specifically, platelets expressed key proteins involved in these processes, including LC3B-I/II, GABARAP-I/II, p62, TOM20, PINK, PARKIN, and LAMP2A, as well as components essential for autophagosome-lysosome fusion, such as VAMP8, RAB7, and STX17 (Supplemental Figure 2A). Furthermore, transmission electron microscopy (TEM) revealed an increased number of phagophore-like structures that initiate autophagosome formation and autophagosome-like structures enclosing cargo for degradation (50). In addition, MPN platelets exhibited mitochondria with disrupted cristae compared with those in HC platelets (Figure 2C and Supplemental Figure 2B).

We evaluated autophagy and mitophagy by immunoblotting in accordance with current guidelines (51, 52). We quantified autophagic flux with LC3B-II and mitophagic flux with TOM20, which are markers of autophagosomes and mitochondria, respectively. In agreement with prior reports (24, 25, 30), we determined that incubating platelets with 50 μM chloroquine (CQ) for 2 hours was sufficient to assess platelet autophagy effectively (Supplemental Figure 2, C and D). Figure 2D outlines the interpretation of these assays: a statistically significant increase in LC3B-II or TOM20 levels between CQ-treated and vehicle control samples indicates active autophagic or mitophagic flux, respectively, whereas a nonsignificant delta indicates inhibited flux.

HC platelets showed robust positive autophagic and mitophagy flux, as indicated by statistically significant differences in the LC3B-II and TOM20 levels between CQ-treated and control cells. In contrast, MPN platelets presented no changes in LC3B-II or TOM20 levels, indicating that both autophagy and mitophagy were blocked (Figure 2, E and F). Furthermore, the heightened baseline LC3B-II levels in MPN platelets, coupled with the greater number of autophagosome-like structures observed via TEM, indicated a higher number of autophagosomes in these platelets. We then aimed to eliminate the possibility that defects in autophagosome formation accounted for the lack of LC3B-II accumulation in MPN platelets incubated with CQ. TEM analysis confirmed the presence of phagophore-like structures and fully formed autophagosome-like structures in both HC and MPN platelets, indicating that the autophagosome formation process is functional (Supplemental Figure 2E). These findings indicated that autophagosome degradation was not detected within the timeframe of this assay.

Collectively, these findings suggest that defects in the autophagic pathways of MPN platelets may account for their increased mitochondrial mass (33, 53) and impaired clot contraction (Figure 2F and Supplemental Figure 2B). Figure 2G summarizes the metabolic and functional defects associated with blocked autophagic pathways in MPN platelets.

### Pharmacological inhibition of platelet autophagy with CQ decreases both mitochondrial respiration and clot contraction in vitro.

**Figure 3:**
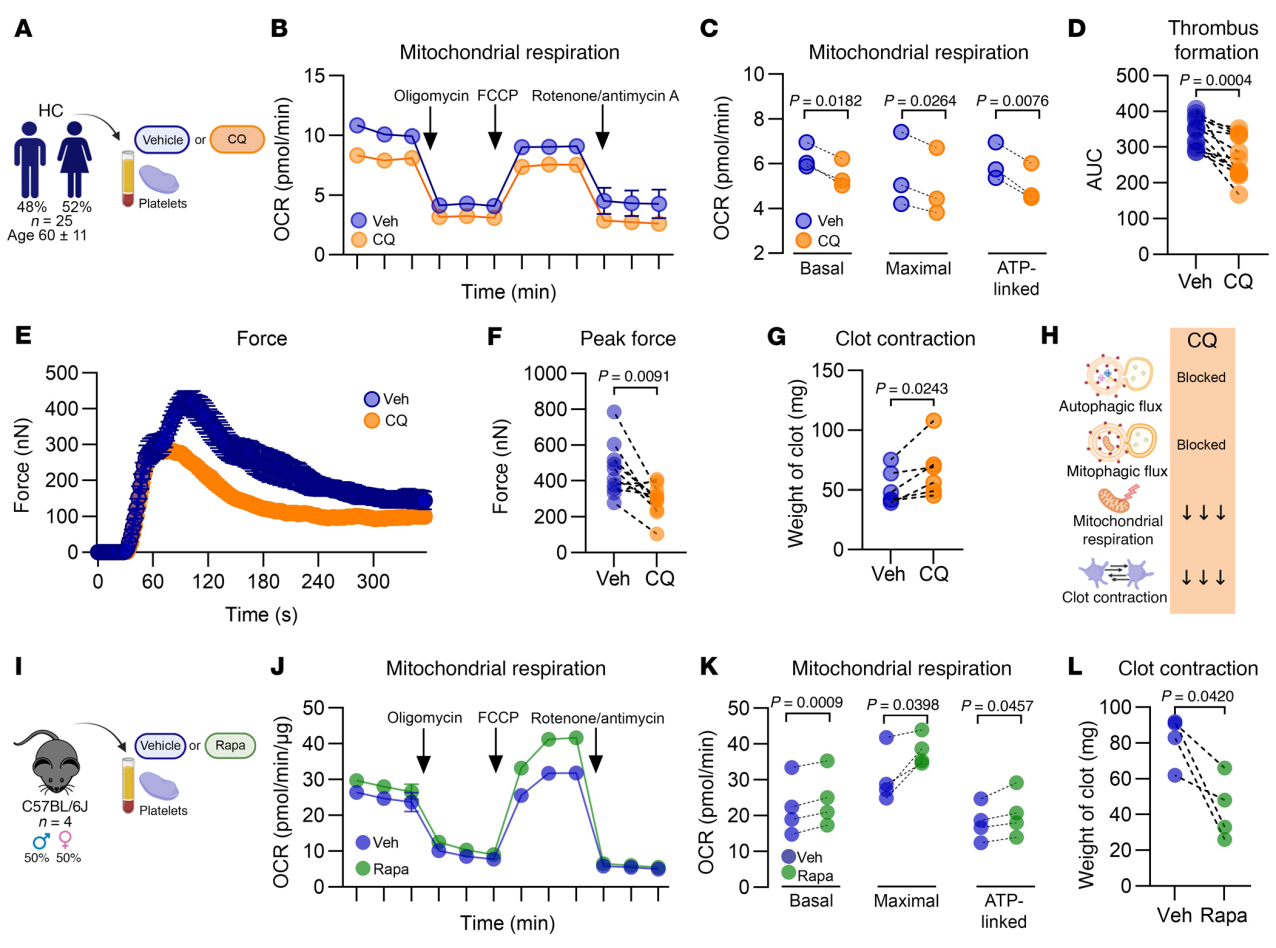
Pharmacological inhibition of autophagy impairs mitochondrial respiration and clot contraction in vitro. (**A**) Experimental approach. (**B**) The OCR of washed platelets (*n* = 3) was measured using a Seahorse XF HS Mini Analyzer (mean ± SEM). (**C**) Basal, maximal, and ATP-linked respiration parameters with before-and-after graphs (*n* = 3); paired *t* test. (**D**) Thrombus formation assay in whole blood using the T-TAS analyzer and PL chips; before-and-after graph (*n* = 12); paired *t* test. (**E**) Platelet strength assay (*n* = 11) and (**F**) peak force, before-and-after graph (*n* = 11); paired *t* test. (**G**) Thrombin-induced clot contraction assay with normalized platelet counts (*n* = 6), before-and-after graph; paired *t* test. (**H**) Summary of the effects of the pharmacological inhibition of autophagy with CQ on platelets from HCs. (**I**) Methodology. (**J**) The OCR of washed platelets (*n* = 4) was measured using a Seahorse XF HS Mini Analyzer (mean ± SEM of 3 independent samples). (**K**) Basal, maximal, and ATP-linked respiration, before-and-after graph (*n* = 4); paired *t* test. (**L**) Thrombin-induced clot contraction assay with normalized platelet counts (*n* = 4); before-and-after graph; paired *t* test.

We examined whether the disruption of platelet autophagic pathways underlies the metabolic and functional defects in MPN platelets. To this end, platelets from healthy volunteers (53 ± 13 years old) were incubated with CQ (Figure 3A). Unlike other autophagy inhibitors, CQ does not alter the baseline levels of LC3B-II (Supplemental Figure 3A), making it the ideal inhibitor for this experiment. Importantly, under these experimental conditions, CQ did not affect platelet viability or baseline activation status (Supplemental Figure 3B). There were no differences in the activation profiles of the CQ-treated platelets compared with those of control platelets after stimulation with thrombin, convulxin, or ADP.

CQ-treated platelets displayed significantly lower mitochondrial respiration, as evidenced by the basal, maximal, and ATP-linked respiration parameters (Figure 3, B and C). Their ability to adhere and form clots on collagen-coated slides was reduced (Figure 3D). The pharmacological inhibition of autophagy also impaired platelet contractility. In both the platelet strength and thrombin-induced assays, clot contraction was decreased (Figure 3, E–G). Furthermore, the platelet strength assay revealed that although CQ treatment shortened the time to maximal peak force (Figure 3, E and F), it reduced clot strength and stability, as indicated by the percentage of force lysis (Supplemental Figure 3, C–D). Similarly, CQ-treated platelets from old mice had decreased mitochondrial respiration and reduced clot contraction (Supplemental Figure 3, E–H).

To investigate the role of mitochondrial respiration in clot contraction, platelets from healthy volunteers were treated with electron transport chain complex inhibitors. These included oligomycin, which inhibits F-ATPase; rotenone, an inhibitor of complex I; and antimycin A, an inhibitor of complex III. Treatment with these inhibitors significantly impaired clot contraction (Supplemental Figure 3I).

Next, we investigated whether rapamycin, an inducer of platelet autophagy (25, 30, 54, 55), enhanced clot contraction in platelets from healthy individuals (Figure 3H). Notably, rapamycin increased basal, maximal, and ATP-linked mitochondrial respiration, as well as clot contraction (Figure 3, I–L).

Collectively, these findings demonstrate that pharmacological blockade of autophagy recapitulates the metabolic and functional defects observed in MPN platelets.

### STX17 regulates platelet metabolism and function by controlling autophagy and mitophagy.

**Figure 4:**
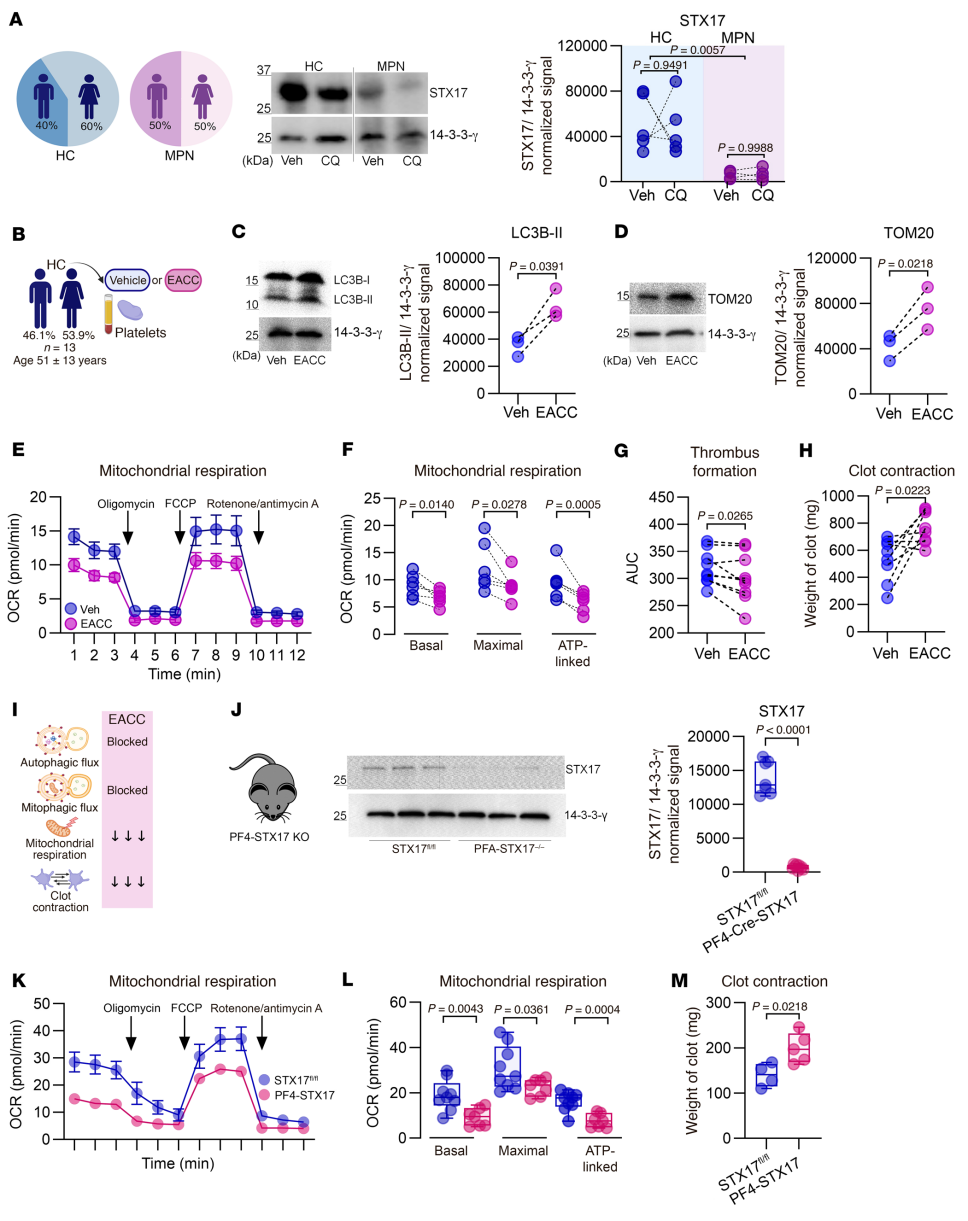
STX17 regulates platelet metabolism and function via autophagy. (**A**) Immunoblot analysis of STX17 in HCs (*n* = 5) and MPN (*n* = 4) platelets; before-and-after graph; 2-way ANOVA, Šídák’s test. Noncontiguous gel lanes are indicated. (**B**) Methodology. (**C**) Immunoblotting for LC3B-II and (**D**) TOM20. Before-and-after graphs (*n* = 3); paired *t* test. (**E**) OCR of washed platelets (*n* = 5) was measured with a Seahorse XF HS Mini Analyzer (mean ± SEM). (**F**) Basal, maximal, and ATP-linked respiration parameters with before-and-after graphs (*n* = 5); paired *t* test. (**G**) Thrombus formation assay in whole blood using the T-TAS analyzer and PL chips, before-and-after graph (*n* = 9); paired *t* test. (**H**) Thrombin-induced clot contraction assay using normalized platelet counts, before-and-after graph (*n* = 8); paired *t* test. (**I**) Summary of the effects of the pharmacological inhibition of STX17 on HC platelets. (**J**) Immunoblot analysis of STX17 expression in platelets from PF4-STX17-KO mice and STX17*^fl/fl^* littermate controls, STX17*^fl/fl^* (*n* = 7) and PF4-STX17-KO (*n* = 9); unpaired *t* test with Welch’s correction. (**K**) OCR of washed platelets measured with a Seahorse XF HS Mini Analyzer; (*n* = 9) and PF4-STX17-KO (*n* = 7). (**L**) Basal, maximal, and ATP-linked respiration parameters, STX17*^fl/fl^* (*n* = 9) and PF4-STX17-KO (*n* = 7); unpaired *t* test for basal and ATP-linked respiration, unpaired *t* test with Welch’s correction for maximal respiration. (**M**) Thrombin-induced clot contraction assay using normalized platelet counts, STX17*^fl/fl^* (*n* = 4) and PF4-STX17-KO (*n* = 5); unpaired *t* test. Box-and-whisker plots (**J**, **L**, and **M**) represent the data distribution.

Our findings of increased autophagosome-like structures and elevated baseline LC3B-II levels in MPN platelets suggested a blockade at the autophagosome-lysosome fusion step. Supporting this, a recent study linked TNF-α to reduced STX17 levels and autophagy blockade in cultured necrotizing fibroblasts (56). Given STX17’s critical role in mediating autophagosome-lysosome fusion (35, 36), we evaluated its level in MPN platelets. Western blot analysis revealed an 89% reduction in STX17 levels in MPN platelets compared with those in age- and sex-matched HC platelets (Figure 4A).

We then investigated whether STX17 plays a modulatory role in platelet function and metabolism and whether its inhibition recapitulates the defects observed in MPN platelets. To this end, HC platelets were treated with ethyl (2-(5-nitrothiophene-2-carboxamido) thiophene-3-carbonyl) carbamate (EACC), a validated STX17-specific inhibitor (Figure 4B) that blocks the translocation of STX17 to fully formed autophagosomes (57). This prevents their fusion with lysosomes, thereby inhibiting autophagic pathways, analogous to the effects observed with CQ treatment. Both autophagy and mitophagy were blocked in EACC-treated platelets, as evidenced by the accumulation of LC3B-II and TOM20 (Figure 4, C–D). These findings indicated that the autophagosomes and mitochondria were not degraded.

Moreover, the specific inhibition of autophagic pathways in platelets by EACC recapitulated the metabolic and functional defects observed in MPN platelets. EACC treatment (as well as CQ treatment) increased ROS levels, including mitochondrial, NADPH-derived, and other sources (Supplemental Figure 3J). It also reduced basal, maximal, and ATP-linked mitochondrial respiration (Figure 4, E and F), decreased clot formation (Figure 4G), and significantly diminished clot contraction (Figure 4, H and I). The role of STX17 was further validated using a platelet-specific KO (PF4-Cre, STX17) mouse model (Figure 4J), whose platelets also had decreased mitochondrial respiration and reduced clot contraction (Figure 4, K–M). Collectively, these findings demonstrate that STX17 serves as a critical modulator of platelet autophagic pathways, mitochondrial respiration, and clot contraction.

### TNF-α downregulates STX17 levels in the MK cell line Meg-01.

**Figure 5:**
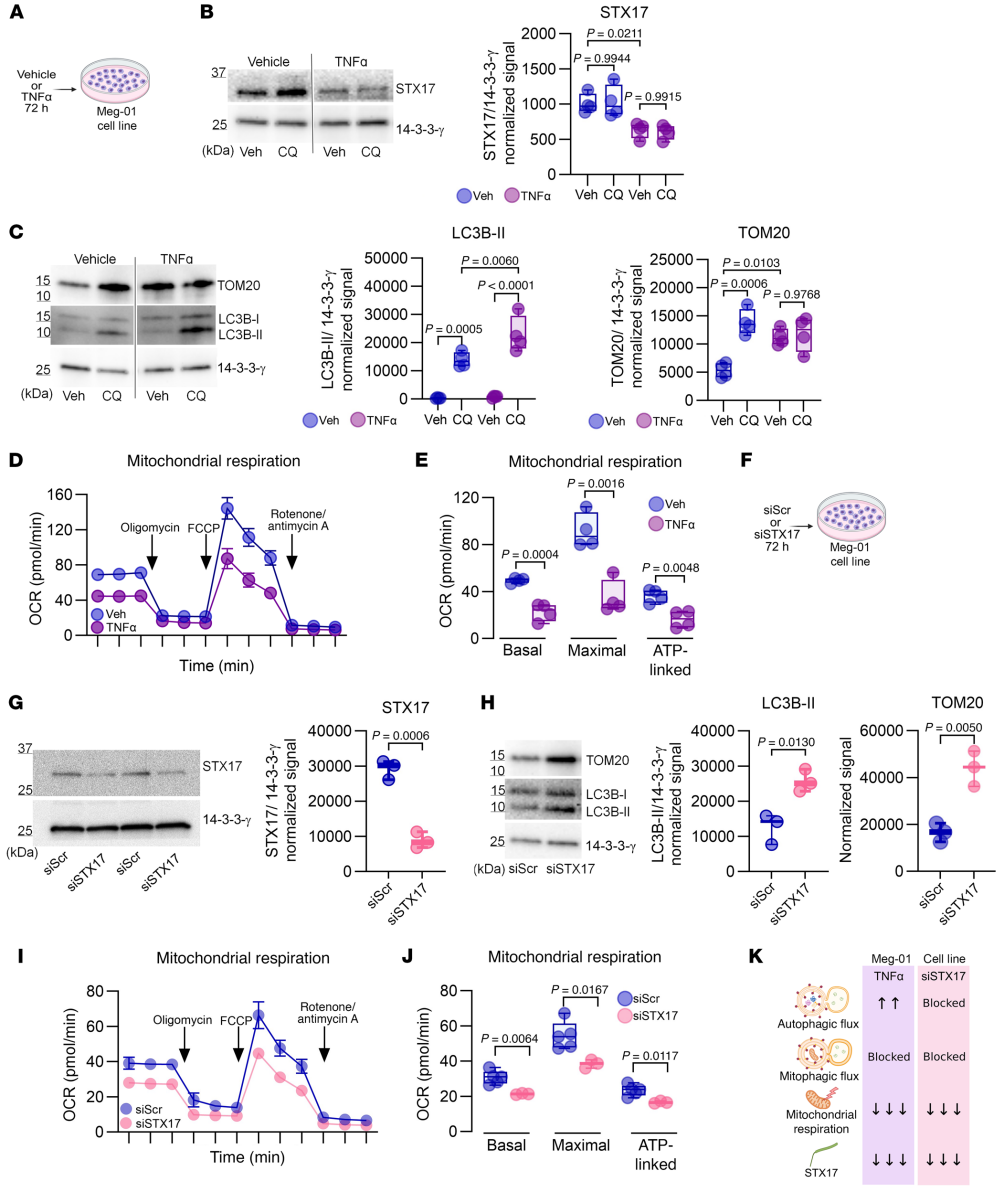
TNF-α downregulates STX17 levels and impairs mitophagy in Meg-01 cells. (**A**) Experimental design. (**B**) Immunoblot analysis of STX17(*n* = 4), 1-way ANOVA, Tukey’s post hoc test. Noncontiguous gel lanes are indicated. (**C**) Immunoblot analysis of LC3B-II and TOM20, (*n* = 4), 1-way ANOVA, Tukey’s post hoc test. Noncontiguous gel lanes are indicated. (**D**) The OCR of Meg-01 cells (*n* = 4) was measured with a Seahorse XF HS Mini Analyzer (mean ± SEM of 3 independent samples). (**E**) Basal, maximal, and ATP-linked respiration parameters (*n* = 3); unpaired *t* test. (**F**) Experimental design. (**G**) Immunoblot analysis of STX17 (*n* = 3); unpaired *t* test. (**H**) Immunoblot analysis of LC3B-II and TOM20 (*n* = 3); unpaired *t* test. (**I**) The OCR of Meg-01 cells (*n* = 4) was measured with a Seahorse XF HS Mini Analyzer (mean ± SEM of 3 independent samples). (**J**) Basal, maximal, and ATP-linked respiration rates (*n* = 4); unpaired *t* test. (**K**) Summary of the effects of TNF-α and siSTX17 on autophagy, mitophagy, and mitochondrial respiration. Box-and-whisker plots (**B**, **C**, **E**, **G**, **H**, and **J**) represent the data distribution.

To determine whether TNF-α directly downregulates STX17 levels in MKs, we utilized the Meg-01 cell line (Figure 5A), a well-established model for studying MK biology (58–60). After treatment with 1.25 ng/mL TNF-α for 72 hours, p65 was phosphorylated without inducing cell death (Supplemental Figure 4, A and B). Compared with the control treatment, this treatment reduced STX17 levels by 67% (Figure 5B). Notably, neither IL-6 nor IL-1β treatment affected STX17 levels (Supplemental Figure 4C). Consistent with these findings, TEM analysis of TNF-α–treated cells revealed increased numbers of autophagosome-like structures containing large dense conglomerates. Additionally, their mitochondria exhibited swelling and disrupted cristae (Supplemental Figure 4D).

Next, we investigated the activity of autophagic pathways in Meg-01 cells via the use of CQ. In TNF-α–treated cells, LC3B-II levels increased significantly, indicating increased autophagic flux. In contrast, TOM20 levels remained unchanged, confirming that mitophagy was blocked (Figure 5C). As expected, neither IL-6 nor IL-1β impaired autophagy or mitophagy (Supplemental Figure 4C). Furthermore, in TNF-α–treated cells, significant reductions in basal, maximal, and ATP-linked mitochondrial respiration (Figure 5, D and E) accompanied mitophagy blockade.

Finally, siRNA knockdown (siSTX17) confirmed the role of STX17 in regulating autophagic pathways in Meg-01 cells (Figure 5F). This approach reduced STX17 levels by approximately 70%, leading to the blockade of both autophagy and mitophagy (Figure 5, G and H). Elevated LC3B-II and TOM20 levels indicate the impaired autophagosome degradation and the accumulation of damaged mitochondria resulting from low STX17 levels. Additionally, STX17 knockdown significantly decreased basal, maximal, and ATP-linked mitochondrial respiration (Figure 5, I and J). Figure 5K summarizes the effects of TNF-α and siSTX17 treatments on Meg-01 cells. Collectively, these findings highlight the regulatory role of TNF-α in modulating STX17 levels and confirm that under noninflammatory conditions, STX17 is critical for autophagy, mitophagy, and mitochondrial respiration.

### Inhibition of autophagic and mitophagic flux in platelets from mice with rheumatoid arthritis and inflammatory bowel disease is linked to low STX17 levels.

TNF-α is a proinflammatory cytokine that is critical not only to the pathogenesis of MPN but also to that of rheumatoid arthritis (RA) and inflammatory bowel disease (IBD) (61–63). These conditions are characterized by imbalanced hemostasis (64, 65). Although an increased incidence of thrombosis is common to all 3 conditions, both thrombotic and hemorrhagic complications are defining features of MPN and IBD (43, 66, 67). Notably, these complications remain the leading causes of death and disability, which underscores the need to better understand the mechanisms underlying TNF-α–driven diseases. Given the established role of TNF-α in these conditions, we investigated its impact on hemostatic balance. Specifically, we assessed whether it directly alters platelet function through autophagy, as observed in MPN platelets.

**Figure 6:**
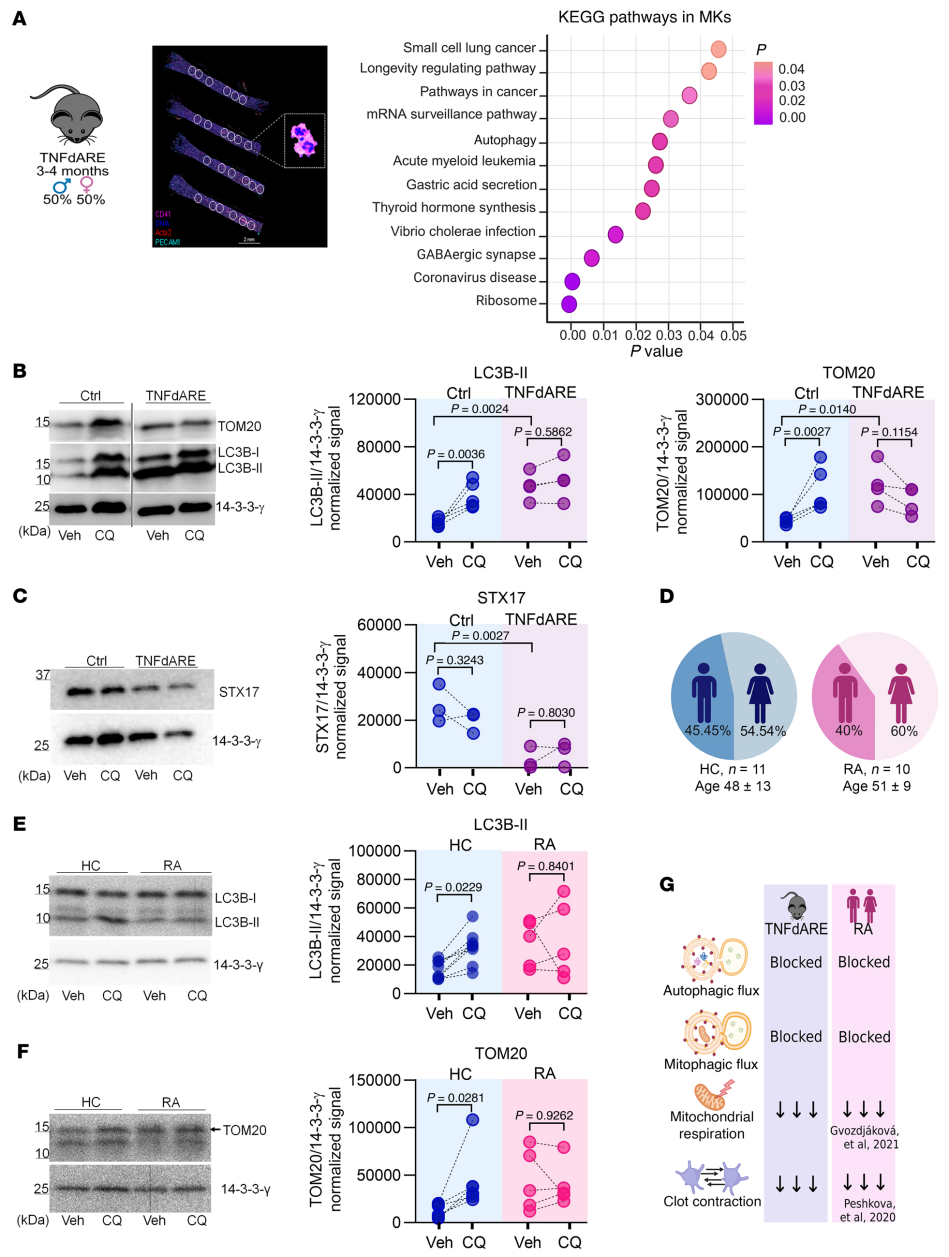
Inhibition of autophagic pathways in platelets from mice with rheumatoid arthritis and inflammatory bowel disease is linked to low STX17 levels. (**A**) Spatial RNA transcriptomics of bone marrow megakaryocytes (MKs); littermate controls (*n* = 1) and TNFdARE (*n* = 1) mice. Immunofluorescence for CD41, ACTA2, PECAM1, and DNA; the circles represent the regions of interest (ROIs) selected for the transcriptomic analysis. Scale bar: 2 mm. KEGG analysis of differentially expressed genes in the control (*n* = 9) and TNFdARE (*n* = 12) groups. (**B**) Immunoblot analysis of LC3B-II and TOM20 in platelets from control (*n* = 5) and TNFdARE (*n* = 4) mice treated with vehicle (PBS) or CQ (50 μM), before-and-after graph; 2-way ANOVA, Šídák’s test. Noncontiguous gel lanes are indicated. (**C**) Immunoblot analysis of STX17 in platelets from Ctrl (*n* = 3) and TNFdARE (*n* = 3) mice, before-and-after graph; 2-way ANOVA, Šídák’s test. (**D** and **E**) Immunoblot analysis of LC3B-II from HCs (*n* = 8) and patients with RA (*n* = 5) and TOM20 from HCs (*n* = 6) and patients with RA (*n* = 5) treated with vehicle (PBS) or CQ (50 μM). Before-and-after graph; 2-way ANOVA, Šídák’s test, Šídák’s test. (**F**) Summary of autophagic flux, accumulation of damaged mitochondria, and platelet hemostasis in TNFdARE mice and patients with RA. (**G**) Summary of autophagic flux.

We began by evaluating platelets from TNFdARE mice, which have constitutively elevated levels of TNF-α and develop RA and IBD by 6 weeks of age (68). TNFdARE platelets displayed significantly lower mitochondrial respiration (Supplemental Figure 5, A and B), a global decrease in purine metabolism (Supplemental Figure 5, C–E), and diminished contraction (Supplemental Figure 5F). These findings mirror those observed in MPN platelets. We then sought to determine the impact of chronically elevated TNF-α levels on the gene expression pathways associated with autophagy in MKs and platelets. Our experiments revealed differential expression of genes associated with autophagy in MKs and with mitophagy in the platelet transcriptomes of TNFdARE mice (Figure 6A and Supplemental Figure 5G). Moreover, these findings corroborate the direct impact of TNF-α on MKs and suggest that defects in autophagic pathways originate in bone marrow platelet precursors.

Autophagic flux analysis of these platelets revealed a blockade of both autophagy and mitophagy, as evidenced by the unchanged LC3B-II and TOM20 levels between the CQ-treated and vehicle samples (Figure 6B). This finding indicated that, within this timeframe, platelets do not actively degrade damaged mitochondria or other cellular components via autophagic pathways. This blockade was accompanied by an 86% reduction in STX17 levels in TNFdARE platelets, mirroring the decrease observed in MPN platelets (Figure 6C).

We subsequently sought to determine whether these findings could be generalized to other TNF-α–driven conditions, such as RA. In platelets from a cohort of newly diagnosed patients with RA, autophagic flux analysis revealed blockade of both the autophagy and mitophagy pathways (Figure 6, D and E). These findings demonstrated that the disruption of autophagic pathways is not exclusive to MPN or TNFdARE platelets. Owing to limited sample availability, STX17 levels could not be determined. Furthermore, although mitochondrial respiration and clot contraction were not assessed in this cohort, previous studies reported reductions in both parameters in platelets from patients with RA (69, 70) (Figure 6F).

Next, we tested the therapeutic potential of TNF-α neutralization in TNFdARE mice. For 20 days, the mice were treated with either a neutralizing anti–TNF-α antibody or an isotype control (Supplemental Figure 5H). Given the estimated 5-day lifespan of mouse platelets, this treatment duration would allow the turnover of 4 platelet generations by MKs exposed to the neutralizing antibody (33). TNF-α blockade restored platelet STX17 levels in all treated mice. Although not statistically significant, the autophagic activity improved in 3 of the 4 mice. However, mitophagic flux remained blocked, with improvement observed in only 1 mouse (Supplemental Figure 5I). Collectively, these findings demonstrate the role of TNF-α in regulating STX17 and platelet function and metabolism under inflammatory conditions (Figure 6G).

### In vivo, TNF-α blockade preserved the STX17 level, autophagy, mitochondrial respiration, and clot contraction in a mouse model of TNF-α–driven aseptic inflammation.

We aimed to evaluate the ability of TNF-α blockade to prevent the inhibition of autophagic pathways in platelets in a mouse model of TNF-α–driven aseptic inflammation. This was the same experimental model in which we initially observed the role of TNF-α in modulating platelet mitochondrial mass and activation (33). This approach allowed us to exclude chronic, in utero–initiated inflammation characteristic of TNFdARE mice as a potential confounding factor.

**Figure 7:**
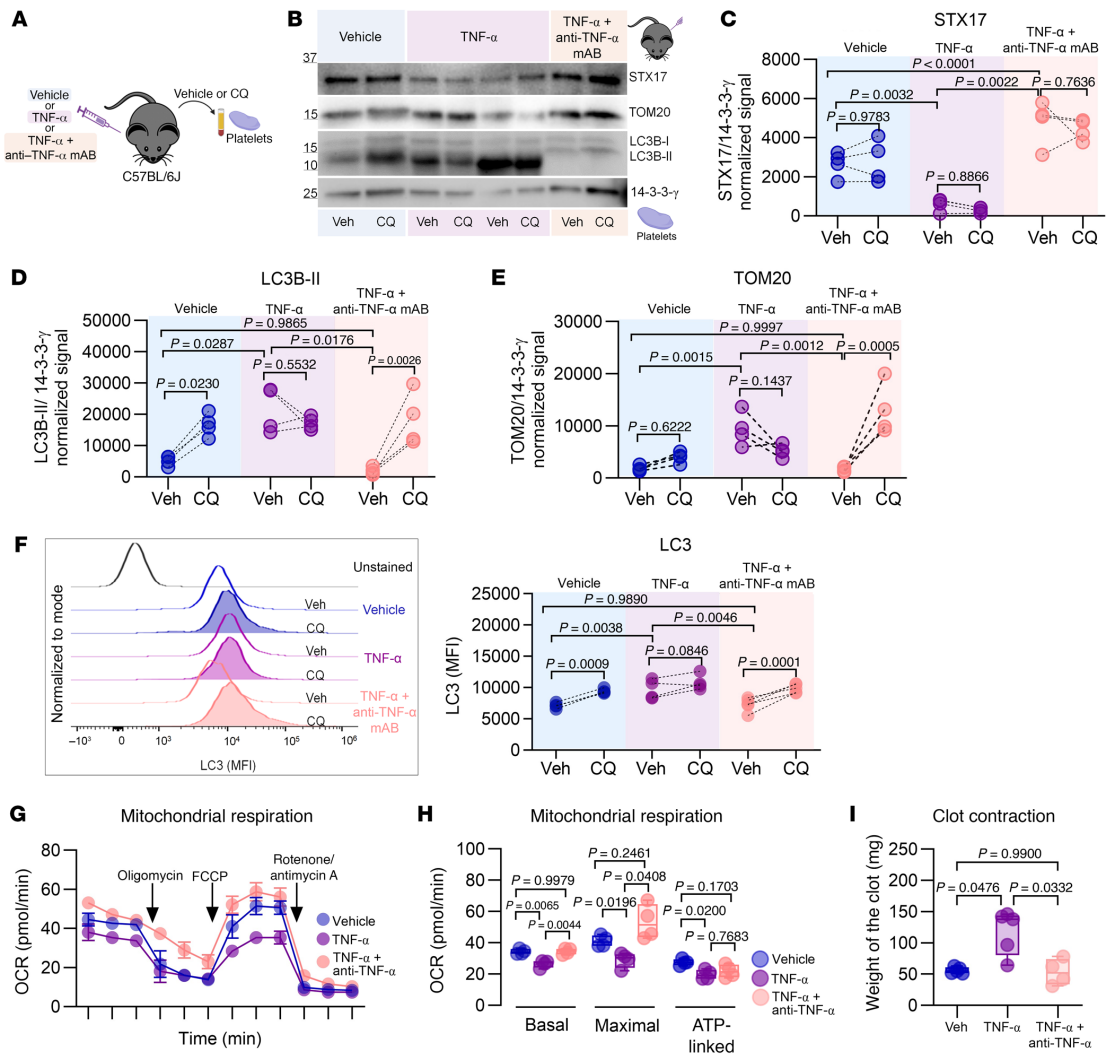
In vivo TNF-α blockade preserves STX17 levels, autophagy, mitochondrial respiration, and clot contraction in a mouse model of TNF-α–driven aseptic inflammation. (**A**) Experimental approach. Young (3-month-old) C57BL/6 mice were treated daily with vehicle (0.01% BSA in PBS), TNF-α, or TNF-α plus an anti–TNF-α mAb (every other day) for 15 days (*n* = 4 each). Platelets were incubated with vehicle (PBS) or CQ (50 μM) for 2 hours to analyze autophagic flux. (**B**) Immunoblotting of STX17, LC3B-II, and TOM20. (**C**) Analysis of STX17, (**D**) LC3B-II, and (**E**) TOM20 levels. Before-and-after graph, *n* = 4 each; 2-way ANOVA, Šídák’s test. (**F**) Quantification of intracellular LC3 by FACS, representative histogram with before-and-after graphs, Veh and TNF-α plus anti–TNF-α mAb *n* = 4, TNF-α *n* = 3; 2-way ANOVA, Šídák’s test. (**G**) The OCR of washed platelets (*n* = 4) was measured with a Seahorse XF HS Mini Analyzer (mean ± SEM of 3 independent samples). (**H**) Basal, maximal, and ATP-linked respiration (*n* = 4), Brown-Forsythe and Welch’s ANOVA tests. (**I**) Thrombin-induced clot contraction assay using normalized platelet counts, Veh (*n* = 4), TNF-α (*n* = 5), and TNF-α plus anti–TNF-α mAb (*n* = 4); Brown-Forsythe and Welch’s ANOVA test. Box-and-whisker plots (**H** and **I**) represent the data distribution.

WT C57BL/6J mice received 1 of 3 treatment regimens: (a) daily PBS-0.01% albumin (vehicle control), (b) daily murine recombinant TNF-α, or (c) daily TNF-α plus anti–TNF-α antibody given every other day (Figure 7A). Compared with the control treatment, the TNF-α treatment alone reduced the STX17 level by 78% (Figure 7, B and C). In contrast, STX17 levels remained unchanged in platelets from mice that received daily TNF-α plus every-other-day anti–TNF-α. Consistent with the preservation of STX17 levels by the neutralizing antibody, both autophagic and mitophagic flux remained intact (Figure 7, B, D, and E). A FACS-based assay that quantifies all the intracellular LC3 isoforms associated with autophagosomes confirmed these findings (Figure 7F). Finally, TNF-α blockade also preserved mitochondrial respiration and clot contraction (Figure 7, G–I). Taken together, these findings confirm the critical role of TNF-α in regulating platelet autophagic pathways, mitochondrial respiration, and clot contraction through the modulation of STX17 levels.

## Discussion

Our previous work established a mechanistic link between TNF-α and the generation of platelets with increased mitochondrial mass and increased reactivity. Transcriptome analysis of MKs from these TNF-α–inflamed mice revealed an association between autophagy-related gene pathways and the observed platelet hyperreactivity (33). Because platelets lack a nucleus and have limited protein synthesis, mitochondrial biogenesis in circulating platelets is unlikely to explain the increased mitochondrial mass (1, 71). This raised the question of how these metabolic changes arise. Because autophagy is critical for regulating cellular energy and maintaining mitochondrial mass and health (21), we hypothesized that the TNF-α–mediated disruption of autophagic pathways in platelets drives these metabolic and functional changes.

This study provides a mechanistic understanding of how TNF-α affects platelet function through autophagy. We demonstrated that reduced STX17 levels in platelets are a hallmark of inflammatory diseases characterized by elevated TNF-α (Figures 4 and 6). STX17 is a critical mediator of autophagosome-lysosome fusion in autophagy; low STX17 levels prevent this fusion, blocking the degradation phase of this pathway. This blockade results in the accumulation of autophagosomes, the buildup of damaged mitochondria, and a reduced supply in the levels of the autophagy-derived molecules required to sustain cellular respiration. Although these platelets have an increased mitochondrial mass, they are also hypometabolic, as demonstrated by reduced ATP levels and impaired basal, maximal, and ATP-linked mitochondrial respiration (Figure 1 and Supplemental Figure 5). Elevated levels of phosphorylated AMPK indicated metabolic deficits in MPN platelets.

TEM analysis of control and MPN platelets provided valuable insights into the autophagosome formation process. Both groups of platelets contained phagophore-like structures, cup-shaped membranes that initiate autophagosome formation, and autophagosome-like structures, namely, double-membrane vesicles enclosing cargo for degradation (Figure 2 and Supplemental Figure 2). These findings confirm that autophagosome formation remains functional in platelets during inflammation and suggest that defective fusion is the primary autophagic defect.

The marked decrease in mitochondrial respiration was not attributable to deficiencies in key proteins of the electron transport chain. For instance, MPN platelets presented elevated levels of electron transport chain proteins, including SDHB, UQCRC2, and COXII, corresponding to complexes II, III, and IV, respectively. These elevations suggest a compensatory mechanism by which MKs and platelets sustain adequate ATP levels.

This work also demonstrated that normal mitochondrial respiration is essential for effective platelet contraction. Pathways likely to be involved include glycolysis, glycogenolysis, oxidative phosphorylation, and fatty acid β-oxidation, which have been shown to be necessary for platelet function under homeostatic conditions (3, 5, 72, 73). According to our findings, autophagy-derived metabolites are fundamental for mitochondrial respiration in platelets. The essential role of autophagy in sustaining cellular metabolism has been shown in other contexts, such as in cancer cell lines and neonatal tissues (12–17, 74).

The inhibition of autophagy, whether by inflammation, drugs, or the platelet-specific deletion of STX17 (Figures 3, 4, and 7, and Supplemental Figure 3), consistently reduced clot contraction. This platelet-driven process is critical for sealing damaged tissues, reducing intraluminal obstruction, stabilizing the clot, and facilitating its organized dissolution by the fibrinolytic system (75). Despite the central role of clot contraction in hemostasis, its regulation in both health and disease is poorly understood. Notably, impaired clot contraction is linked to inflammatory diseases characterized by hemostatic imbalance, including RA, acute ischemic stroke, sickle cell disease, systemic lupus erythematosus, and venous thromboembolism (70, 76–79). In these conditions, both thrombosis and bleeding are complications leading to morbidity and mortality, and both are caused by defective clot contraction.

Poor contractile clots may cause thrombosis by enlarging to the point they obstruct blood flow, while their weakened clot strength can cause dislodgement, resulting in embolization or hemorrhage during vascular repair. The lack of standardized methods has made it difficult to carry out mechanistic studies of clot contraction in vivo and in real time. Nevertheless, one study in mice provided direct evidence that clot contraction occurs in vivo (80). Lending further credence for a role in thrombosis, defective ex vivo clot contraction on day 1 after surgery significantly predicted venous thrombosis risk in patients with brain tumor (81).

An important role for autophagy in clot contraction is supported by our finding that rapamycin, a drug that enhances autophagic flux by inhibiting mTOR (82), augments clot contraction. In platelets from healthy volunteers, rapamycin treatment increased clot contraction and mitochondrial respiration, particularly the maximal respiratory capacity (Figure 3). This effect may result from increased availability of autophagy-derived biomolecules supporting cellular respiration. This suggests that the use of autophagy-enhancing drugs represents a potential new therapy to restore platelet function and hemostatic balance in inflammatory diseases.

Experiments using Meg-01 cells provided mechanistic insights into the regulation of STX17 in MKs and platelets under inflammatory conditions. TNF-α reduced STX17 levels in Meg-01 cells, whereas IL-6 and IL-1β had no effect (Figure 5 and Supplemental Figure 4). Since platelets lack significant protein synthesis capacity, their protein composition is largely determined by MKs. As a result, the low STX17 levels in MKs are reflected in the platelets they produce, rendering these platelets less capable of effectively performing autophagy and mitophagy upon entering the circulation. Data from our TNF-α neutralization studies in mice provide 2 key insights into the regulation of STX17 under inflammatory conditions. First, blocking TNF-α signaling in a model of aseptic inflammation prevented STX17 downregulation and preserved autophagic pathways in platelets (Figure 7). Second, in TNFdARE mice exposed to elevated TNF-α during in utero development, the intervention restored STX17 levels. Although some mice exhibited improved autophagy, the observed changes were not statistically significant (Supplemental Figure 5). These results indicated that the duration and intensity of inflammation may determine the degree to which autophagy is impaired, as observed in patients with osteoarthritis (83–85). Primary chondrocytes from these patients exhibit reduced expression of FOXO1/3 and SIRT1, key regulators of several autophagy-related genes (83, 84). Osteoarthritis synoviocytes also display increased levels of *N*^6^-methylation of ATG7 mRNA, resulting in decreased protein levels (85). Because of the critical role of ATG7 in autophagosome formation, its reduced expression disrupts autophagic pathways. These findings imply that elevated TNF-α in the joints of these patients may impair autophagy through transcriptional and epigenetic mechanisms. Similar processes may underlie autophagy blockade observed in TNFdARE mice, potentially explaining why TNF-α neutralization alone was insufficient to fully restore autophagy and mitophagy in this RA model.

The regulation of STX17 levels and activity remains poorly understood, particularly in the context of inflammatory conditions. A recent study reported that TNF-α induces the proteolysis of STX17 in necroptotic fibroblasts (56). Although this aspect was beyond the scope of our study, we did not observe truncated STX17 in platelets from patients with MPN or in TNFdARE mice. Nevertheless, the targeted deletion of full-length STX17 in platelets functionally recapitulated the phenotype observed across various inflammatory models, underscoring its essential role in platelet function.

In addition to its primary role in regulating the autophagosome-lysosome fusion step of autophagy, STX17 triggers mitophagy of damaged mitochondria (34, 86). This may explain why downregulation of STX17 by TNF-α or siRNA consistently inhibited mitophagy in Meg-01 cells. Interestingly, autophagy increased in TNF-α–treated cells but was blocked in STX17-silenced cells, suggesting that MKs may activate compensatory nuclear programs to sustain autophagic function and mitigate inflammation-associated stress. Identifying the molecular drivers that underly these divergent responses of autophagy and mitophagy to TNF-α, as well as their implications for megakaryopoiesis, are critical areas for future research.

STX17 may also play a role in modulating mitochondrial fission and fusion, dynamic processes that enable cells to maintain their mitochondrial network by selectively exchanging mitochondrial contents. Proper mitochondrial dynamics are essential for the removal of damaged mitochondria via mitophagy (20). In cardiomyocytes, STX17 facilitates mitophagy by phosphorylating and activating DRP-1, a key regulator of mitochondrial fission (87). Studies on platelet-specific mitofusin-2–KO mice highlight the importance of mitochondrial dynamics for platelet function. These mice exhibit impaired hemostatic function and a reduced platelet lifespan. This dysfunction is evidenced by exacerbated pulmonary hemorrhage in LPS-induced acute lung injury and increased clearance of platelets from the circulation (7). Altogether, these findings emphasize the crucial need to define STX17’s role in mitochondrial dynamics in MKs and platelets under both homeostatic and inflammatory conditions.

The JAK2 V617F mutation increases TNF-α secretion by mononuclear myeloid cells in MPN (38, 39). Our data suggest that the TNF-α–driven downregulation of STX17 is the primary cause of autophagy blockade in MPN platelets. However, we cannot rule out the possibility that the mutation could also alter autophagy through inflammation-independent mechanisms. Nevertheless, these data suggest that targeting TNF-α signaling pathways in MPNs may restore the hemostatic balance in this disease, but this requires further study.

Collectively, our findings demonstrate that TNF-α disrupts the hemostatic balance by downregulating STX17, impairing autophagic pathways, and reprogramming the metabolism of MKs and platelets. These metabolic disruptions, observed across human and mouse models of inflammation, suggest a mechanism that can be generalized to diverse inflammatory diseases. Importantly, these disruptions may contribute to the increased incidence of thrombotic and hemorrhagic events observed in such conditions and represent a compelling therapeutic target.

## Methods

Further information can be found in Supplemental Methods.

### Sex as a biological variable.

Our study included comparable numbers of male and female human participants and animals, and similar findings were observed in both sexes. However, we did not conduct explicit analyses to evaluate sex as a biological variable.

### Human volunteers.

We collected samples between April 29, 2021, and January 12, 2023. The inclusion criteria required a diagnosis of JAK2 V617F–positive PV, and 90% of patients were receiving treatment at the time of sampling. The RA cohort included newly diagnosed individuals who met the American College of Rheumatology/European Alliance of Associations for Rheumatology (ACR/EULAR) 2010 classification criteria (88) or were diagnosed with RA by a board-certified rheumatologist; they may have received oral corticosteroids but were not receiving other disease-modifying antirheumatic drugs.

Healthy age- and sex-matched controls were included and selected via a disease-screening questionnaire. HC donors taking antithrombotic or antiplatelet drugs were excluded. The demographic and relevant clinical data for the HC, MPN, and RA cohorts are summarized in Supplemental Table 1. Blood samples were collected in citrate (0.109 M, 3.2%) or BAPA tubes.

### Resource table.

Detailed information regarding the antibodies, reagents, and software utilized in this study can be found in the Supplemental Material, Supplemental Tables 2–4.

### Platelet isolation.

Mouse and human platelets were isolated via centrifugation as previously described (33, 89, 90). Briefly, whole blood was centrifuged to separate platelet-rich plasma (PRP). To obtain washed platelets, PRP was then incubated with PIG2 (1.5 μg/mL) for 3 minutes and subsequently centrifuged. The isolated platelets were resuspended in Tyrode’s buffer (pH 7.3). The purity of platelet samples used in experiments was assessed by flow cytometry and confirmed to be up to 99% (Supplemental Figure 1N).

### Statistics.

Normal distributions, outliers, and variances among groups were assessed (refer to figures and the raw data sheet for details). Normality was evaluated using the D’Agostino & Pearson, Anderson-Darling, Shapiro-Wilk, and Kolmogorov-Smirnov tests, and outliers were identified using ROUT analysis. Depending on the experimental design and the assumptions of normality and variance, 2-tailed paired or unpaired *t* tests were used for 2-group comparisons, with Welch’s correction applied for unequal variances. Comparisons among 3 or more groups were analyzed using 1-way ANOVA followed by Tukey’s post hoc test, and 2-factor experiments were evaluated using 2-way ANOVA with Šídák’s multiple-comparison test. Correlation analysis was conducted using Correlation Matrix Online Software: Analysis and Visualization (91), and the resulting correlation matrix was plotted in GraphPad Prism v9.5.1. Statistical significance was set at *P* less than 0.05, and all analyses were performed using GraphPad Prism v9.5.1. All box-and-whisker plots display the mean (horizontal line within the box), the 25th and 75th percentiles (lower and upper bounds of the box), and whiskers indicating the minimum and maximum values. All individual data points are shown.

### Study approval.

Informed consent was obtained in accordance with a protocol approved by the Colorado Multiple IRB (COMIRB 00-004, 20-2318, 10-0477, and 16-2427) and the Western Institutional Review Board (WIRB)-Copernicus Group IRB (20141589).

This study was designed in accordance with the ARRIVE guidelines and approved by the IACUC at the University of Colorado Anschutz Medical Campus (077) and Bloodworks Northwest Research Institute (658-01).

### Data availability.

The values underlying all the graphed data and reported means presented in the main text and supplemental materials are provided in the Supporting Data Values file. Spatial transcriptome data are also provided in the Supporting Data Values file. The RNA-Seq data are available in NCBI’s Gene Expression Omnibus database (GEO GSE282993).

## Author contributions

GRS, JCM, GD, OE, PM, AT, JAL, and PDC conceptualized the study and designed the methodology. GRS, JCM, ACP, GD, MLF, OE, GH, DL, EW, KDD, and PDC conducted the investigation. GRS, JCM, BMM, OE, GH, EW, KJ, LHT, TN, and ADA performed the formal analysis. GRS, JCM, GD, KJ, LHT, and TN curated the data. GRS wrote the original draft. GRS, JCM, BMM, ACP, GD, GPES, OE, GH, GD, EW, KJ, MLF, KDD, TN, AT, PM, JAL, and PDC reviewed and edited the manuscript. GRS, JCM, OE, GH, and TN performed visualization. BMM, CJ, MRK, MMJ, and PDC provided resources. PDC was responsible for supervision, project administration, and funding acquisition.

## Supplementary Material

Supplemental data

Unedited blot and gel images

Supplemental video 1

Supplemental video 2

Supporting data values

## Version 1 06/10/2025

In-Press Preview

## Version 2 08/01/2025

Electronic publication
