# Supplementary material for: TNF-α impairs platelet function by inhibiting autophagy and disrupting metabolism via syntaxin 17 downregulation
Source: J Clin Invest. 2025 Jun 10;135(15):e186065. doi: 10.1172/JCI186065 (PMC12321402; doi:10.1172/JCI186065)
Supplement: Supplemental data [file jci-135-186065-s025.pdf]

TNFα impairs platelet function by inhibiting autophagy and disrupting metabolism via Syntaxin-17 downregulation

Guadalupe Rojas-Sanchez[1], Jorge Calzada-Martinez[1], Brandon McMahon[2], Aaron Petrey[3], Gabriela Dveksler[4], Gerardo P. Espino-Solis[5], Orlando Esparza[6], Giovanny Hernandez[6], Dennis Le[6], Eric Wartchow[7], Ken Jones[8], Lucas Ting[9], Catherine Jankowski[10], Marguerite Kelher[11], Marilyn Manco-Johnson[12], Marie L. Feser[13], Kevin D. Deane[13], Travis Nemkov[14], Angelo D´Alessandro[14], Andrew Thorburn[15], Paola Maycotte[16], José A. López[1,17], and Pavel Davizon-Castillo[1,18*]

[1]Bloodworks Research Institute, Seattle, WA, USA; [2]University of Colorado School of Medicine Hematology- Anschutz Medical Campus, Aurora, CO, USA; [3]Department of Pathology, Division of Microbiology and Immunology, University of Utah, Salt Lake City, UT, USA; [4]Department of Pathology, Uniformed Services University of the Health Sciences, Bethesda, MD USA; [5]Laboratorio Nacional de Citometría de Flujo, Facultad de Medicina y Ciencias Biomédicas, Universidad Autónoma de Chihuahua, Circuito Universitario Campus Universitario II, 31125 Chihuahua, México; [6]Department of Pediatrics Hematology/Oncology and Bone Marrow Transplantation, University of Colorado Anschutz Medical Campus, Aurora, CO, USA; [7]Electron Microscopy Laboratory, Children's Hospital Colorado, Aurora, CO, US; [8]Bioinformatics Solutions, LLC, Sheridan, WY, USA**;** [9]Stasys Medical, Seattle, WA, USA; [10]University of Colorado College of Nursing, University of Colorado-Anschutz Medical Campus, Aurora, CO, USA; [11]University of Colorado School of Medicine, Department of Surgery, University of Colorado-Anschutz Medical Campus, Aurora, CO, USA; [12]Department of Pediatrics, Hemophilia and Thrombosis Center, University of Colorado-Anschutz Medical Campus, Aurora, CO, USA; [13]Division of Rheumatology, University of Colorado Anschutz Medical Campus, Aurora, CO, USA; [14]Biochemistry and Molecular Genetics Department, University of Colorado-Anschutz Medical Campus, Aurora, CO, USA; [15]Department of Pharmacology, University of Colorado-Anschutz Medical Campus, Aurora, CO, USA; [16]Centro de Investigación Biomédica de Oriente, Instituto Mexicano del Seguro Social, Puebla, México; [17]Division of Hematology and Oncology, University of Washington, Seattle, WA, USA; [18]Department of Pediatrics, Hematology/Oncology, University of Washington, Seattle, WA, USA.

***Corresponding author**:
Pavel Davizon-Castillo, MD
Associate Member
Bloodworks Northwest Research Institute

Table of Contents

Materials and Methods…………………………………………………………………….... 1

Supplementary tables.…………………………………………………………………….. 7

Supplementary figures……………………...……………………………………………… 12

Legends for videos...………………………………………………………………….... 21

References………………………………………………………………………......….. 22

Mouse strains and housing conditions. Young (8–12 weeks of age) or old (>18 months of age) C57BL/6J mice (RRID: IMSR JAX:000664) were obtained from the Jackson Laboratory. TNFdARE mice were provided by Dr. Edwin De Zoeten of the Mucosal Inflammation Program, University of Colorado Anschutz Medical Campus. TNFdARE mice were backcrossed with C57BL/6J mice. STX17 fl/fl mice were obtained from GemPharmatech and crossed with PF4-Cre mice (Jackson Laboratory) to generate a platelet and MK-specific knockout. All mice were genotyped by Transnetyx. The mice were maintained in-house in a controlled environment of 22 ± 2 °C and 50 + 10% relative humidity under a 12-hour light/dark cycle. Mice were group-housed (maximum five mice per cage). A normal chow diet and water were provided *ad libitum.* A veterinarian monitored the health of the mice daily for signs of distress or suffering. Age- and sex-matched littermates were employed as experimental controls in the study.

Mouse treatment. As shown in Supplementary Figure 5H, TNFdARE mice or littermate controls received intraperitoneal (IP) injections of either an isotype control antibody or an anti-TNFα neutralizing antibody, each administered at 10 μg/g of body weight every other day for a total of 20 days. As shown in Figure 7A, C57BL/6J mice received daily intraperitoneal (IP) injections for 15 days of one of the following: vehicle (PBS with 0.01% BSA), recombinant murine TNFα (40 ng/g body weight), or a combination of TNFα and an anti-TNFα neutralizing antibody (10 μg/g body weight).

Flow cytometry. Platelet activation and platelet granule content were assessed using anti-PAC-1 and anti-P-selectin antibodies, respectively, under resting conditions or after stimulation. The washed platelets were stimulated with 0.075 U/mL thrombin for 2 and 5 minutes, 1.25 µM ADP for 10 minutes, or 50 ng/mL convulxin for 3 minutes. Platelets were identified using an anti-CD41 antibody. ROS levels were quantified using CellROX Green. Events were acquired on a CytoFLEX flow cytometer (Beckman Coulter). To evaluate the contribution of different ROS sources, platelet-rich plasma was pre-treated with either apocynin (300 µM) or Mito-TEMPO (20 µM), or vehicle (0.001% DMSO-Tyrode's buffer) for 60 minutes. Following pre-treatment, platelets were incubated with vehicle (0.001% DMSO-Tyrode's buffer), chloroquine (CQ, 50 µM), or EACC (5 µM) for 2 hours. FCCP and thrombin stimulation were used as ROS-positive controls (Data not shown). Platelets were then stained with CellROX Green, and events were acquired using a CytoFLEX flow cytometer (Beckman Coulter).

Bioenergetics. The platelets were gently resuspended in Tyrode's buffer and counted before seeding. For Figure 1B, C, and Supplementary Figure 1B, we used an XFe24 analyzer with $22x10^6$ washed platelets per well. An XF HS Mini Analyzer with 1×10^6 washed human platelets, $5x10^6$ murine platelets, or 7×10^3 Meg-01 cells per well was used for the other assays. Mitochondrial respiration was measured using the mitochondrial stress test (Agilent) according to the manufacturer's protocol.

The figures present data without normalization; however, we confirmed that the relationship between variables remained consistent even when the data were normalized to protein content (data not shown), indicating that normalization does not alter the interpretation of the results.

Autophagy enhancement assay. Platelet-rich plasma was incubated with rapamycin (Rapa) (200 nM) for two hours as previously described (1, 2).

Pharmacological inhibition of STX17. Platelet-rich plasma was incubated with EACC (5 µM) for two hours as previously described (3).

Inhibition of mitochondrial respiration. Washed platelets were treated with oligomycin (1.5 µM), rotenone (0.5 µM), and antimycin A (0.5 µM) for two hours.

Clot characterization

1. *Total thrombus formation analysis.* Thrombus formation was analyzed in a flow chamber with PL chips (collagen-coated) using 320 µL of whole blood collected in BAPA tubes.
2. *Platelet strength assay (PSA).* A total of 400 µL of citrate-containing blood was loaded into microforce cartridges and analyzed using the ATLAS system, as previously described (4).
3. *Thrombin-induced clot contraction assay.* Glass tubes were filled with 745 µL of Tyrode's buffer and 5 µL of red blood cells (RBCs). PRP was normalized to a platelet count of 250,000 for humans and 400,000 for mice in a final volume of 200 µL. Thrombin (1 U/mL) was added, and the mixture was incubated at 37 °C for 60 minutes. Clot contraction was assessed by the weight of the clot, as previously reported (5, 6).

Electron microscopy. Platelets from five HCs and five MPN patients were evaluated by TEM. Meg-01 cells from up to four different passages were also analyzed. The pellets were cleared and resuspended in warm Histo-Gel. The pellet was processed for electron microscopy by rinsing in

PBS, fixing in 1% osmium tetroxide for 1 hour, rinsing again in buffer, and dehydrated in a graded ethanol series (50%, 75%, 90%, 100%X2) with 15-minute changes. Following dehydration, the cell pellet was embedded in EPON epoxy resin and allowed to cure overnight. Then, 80 nm thick ultrathin TEM sections were collected on copper grids and post-stained using lead citrate and UranyLess contrasting agents. Images were acquired using a JEOL JEM-1400plus TEM (Tokyo, Japan) operated at 120kV and equipped with a Gatan Orius SC1000B digital camera. Autophagic structures such as phagophore-like, and autophagosome-like structures were identified according to published guidelines (7-11). Platelet morphology and δ-granules were assessed as previously described (12-15) (Supplementary Figure 1A). An expert in electron microscopy corroborated all the identified structures. A total of 12 fields per group were evaluated.

Metabolomics. Platelets were extracted in prechilled (-20 °C) methanol:acetonitrile:water (5/3/2 v/v/v), vortexed for 30 minutes at 4 °C, and extracts isolated by centrifugation at 18k × g for 10 minutes 4 °C. The extracts were resolved in a Kinetex C18 Column (Phenomenex) accoupled with a guard column (SecurityGuard™ Ultracartridge). The separation was done at 25 °C with a flow rate of 250 µL/min using a mixture of 5% acetonitrile, 95% water, and 0.1% formic acid. Analyses were performed with a Vanquish UHPLC system (Thermo Fisher Scientific) coupled online to a Q Exactive mass spectrometer (Thermo Fisher Scientific) as previously described (16). Graphs and statistical analysis (unpaired t Test) were performed with R (17) or GraphPad Prism v9.5.1.

Autophagic flux measurement. Platelet-rich plasma was incubated with CQ (50 µM) or vehicle (PBS) for two hours. Meg-01 cells were incubated with CQ (25 mM) or PBS for four hours. Autophagic flux was assessed using two methods, following established guidelines (7). For the first method, western blot analysis was used to measure the accumulation of LC3B-II in the presence or absence of CQ. The second method involved the use of flow cytometry to quantify LC3 in autophagosomes. For the flow cytometry assay, washed platelets were incubated with digitonin and 5% goat serum for 30 minutes following CQ treatment. The cells were then fixed and stained with an anti-LC3 antibody recognizing all the LC3 isoforms. Events were acquired using a CytoFLEX flow cytometer (Beckman Coulter).

Cell culture

1. *Cell culture conditions.* The Meg-01 cell line was acquired from ATCC and was cultured in RPMI media supplemented with 10% fetal bovine serum, and 1% antibiotics. Cells were

not used after 6 months from thawing. The cell line was cultured in a humidified atmosphere of 95% air/ 5% CO2 at 37 °C.

2. *Cell viability.* A total of 1 × 10^5 Meg-01 cells were treated with TNFα (0.625, 1.25, 2.5, or 20 ng/mL) or vehicle (0.01% albumin in PBS) for 72 hours (Supplementary Figure 4B). Similarly, the same number of cells were treated with CQ (12.5, 25, or 50 µM) or vehicle (PBS) for 4 hours (Supplementary Figure 4F). Following treatment, the cells were washed and stained with anti-CD41-PE, bovine lactadherin, and propidium iodide. The gating scheme is presented in Supplementary Figure 3G. Meg-01 cells subjected to heat shock served as a positive control for apoptotic cells.

3. Cytokine treatment. A total of 1 × 10^5 Meg-01 cells were treated with TNFα (1.25 ng/mL), IL-6 (6.25, 12.5, 25, or 50 ng/mL), IL-1β (1.25, 2.5, 5, or 10 ng/mL), or vehicle (0.01% albumin in PBS) for 72 hours. Afterward, to analyze their autophagic flux status, the cells were treated with CQ (25 µM) or vehicle (PBS) for 4 hours. Finally, the cells were lysed and analyzed by western blotting.

4. *Transfection.* Lipofectamine (Invitrogen) was used to transfect Meg-01 cells. Scramble siRNA (siScr, Ambion) was used at a final concentration of 1 pmol, and siSTX17 (Ambion) was used at a final concentration of 2.5 pmol. The cells were harvested for experiments 72 hours posttransfection.

Western blot. A total of 80 µg of platelet lysate was loaded per lane, and 25 µg of protein of MEG-01 cell lysate was loaded. Membranes were probed overnight with the indicated primary antibody (1:1000, see Supplementary Table 2 for the list of the antibodies used). Immunoblotting was performed using sections of the western blot membrane containing the corresponding molecular weight markers and primary protein targets. Proteins were detected by chemiluminescence using the western lightning plus-ECL (Perkin Elmer). A digital blot scanner (Syngene, G: BBOX Chemi XX6) was used for all blots, except for Figures 4C-D, 4J, 5G, 6 D-E, and Supplementary 2A blot 3**,** which were scanned with a different digital scanner was used (LG, ImageQuant 350). The exposure conditions can be found in Supplementary Table 3. Because the transfer efficiency varies for proteins of different molecular weights, we used actin, which is similar in size to AMPK, as the loading control. Validations studies showed no differences when normalizing AMPK levels to

total AMPK, actin and 14-3-3γ (data not shown). Due to its size, 14-3-3-γ served as the loading control for low molecular weight proteins. Additionally, its expression is unaffected by gender, age, or disease, making it a reliable choice for translational platelet research (18-20). The expected molecular weight of full-length STX17 was validated by comparison to its expression in platelets and liver and lung tissue (Data not shown).

Densitometry analysis. Raw 16-bit TIF files were processed in ImageJ and lanes were selected using the rectangle tool. Lane profiles were plotted with the gel analysis plug-in. The integrated area within the band profile was measured using the magic wand tool to obtain raw densitometry values. To account the for variability inherent in the western blot process and allow for reliable relative quantification, we calculated a lane normalization factor (LNF) using the following formula: LNF = observed signal of housekeeping protein for each lane / highest observed signal of housekeeping protein on the blot. The normalized experimental signal (NES) was then calculated using the formula: NES = observed experimental signal / LNF as described previously (21).

Bulk RNA sequencing. RNA was extracted from the platelets of TNFdARE and littermate control mice (n = 4 per group). The mice were aged 8–12 weeks and sex-matched, with an equal number of females and males. The extraction was performed via the use of TRIzol (Invitrogen). RNA was then treated with DNase (Qiagen) and purified using columns (Qiagen). The RNA libraries were constructed and sequenced as dual-pass 150 bp reads on an Illumina NovaSeq 6000 sequencer. Sequencing was performed at the University of Colorado Medical Campus Cancer Genomics Shared Resource Core Facility (RRID:SCR_021984). The derived sequences were analyzed as previously described (22-24). Genes with a significance of $p < 0.05$ were uploaded to Enrichr for KEGG pathway analysis (25). Differentially expressed pathways were plotted in R studio (17) using the open-source Ggplot2 package (26).

Spatial transcriptomics. The humeri of TNFdARE mice and littermates (one mouse per group) were fixed, decalcified, embedded in paraffin, mounted, and shipped to NanoString for GeoMx RNA next-generation sequencing (27). Sample preparation was performed as described by NanoString GeoMx RNA-next generation sequencing slide preparation manual (27). Bone marrow sections were first labeled with a Leica autostainer with the following markers: CD41 (1:100, Abcam), ACTA2 (1:200, Abcam), PECAM1 (1:100, R and D Systems), and DNA (Thermo Fisher,

1:10). We used these markers to select regions of interest (ROIs) for sequencing to ensure MK enrichment and a small fraction of precursor cells were positive for CD41 and CD31 (Figure 6A). Then, for sequencing, samples were prepared with the NovaSeq 6000 S2 Reagent Kit v1.5–100 cycles (Illumina). Once the ROIs were identified (sections in circles in Figure 6A), the MK-specific ROIs were exposed to UV light to liberate gene expression probes and quantify expression for liberated RNA probes. As in for bulk RNA sequencing, the normalized gene expression for each gene, in counts per million (CPM), was derived from the raw probe counts for a gene divided by the total number of probe counts (in units of millions) in that sample. Differential expression was analyzed as previously described(22), and genes that were significant at $p < 0.05$ were submitted to Enrichr for KEGG pathway analysis (25).

Illustration creation. Figures 1A, E, K; Figures 2D, G; Figures 3A, H, I; Figures 4A, B, I, J; Figures 5A, F, K; Figures 6A, D, F; Figures 7A; Supplementary Figure 3F; and Supplementary Figure 5H in this study were created using BioRender.com.

	HC		MPN		RA	
	n	%	n	%	n	%
	63	100	55	100	10	
**Age, mean ± SD**	53.66 ± 13.92		61.08 ± 16.19		51.2 ± 9.94	
**Range age years**	24-89		14-87			
**Female assigned at birth**	39	61.9	34	61.8	6	60
**Male assigned at birth**	24	38.1	21	38.2	4	40
**No medications reported**	63	100	7	12.7	10	100
**Aspirin**			28	50.9		
**Anticoagulation**			17	30.9		
**Hydroxyurea**			27	49.1		
**JAK2 inhibitors**			7	12.7		

Supplementary Table 1. Relevant clinical and demographic data of HCs, and patients with MPN and RA. Demographic data of self-reported healthy controls (HCs, *n*= 63) and patients with JAK2 V617F PV myeloproliferative disease (hereafter referred to as MPN, *n*= 55), and patients with rheumatoid arthritis (RA, *n*= 10) were obtained.

REAGENT or RESOURCE	SOURCE	IDENTIFIER
**Antibodies**		
BD™ FITC Mouse Anti-Human PAC-1	BD Bioscience	Cat#340507; RRID: AB_2230769
PE/Cyanine7 anti-human CD41 Antibody	Biolegend	Cat#303718; RRID: AB_10899413
Recombinant Anti-CD41 antibody [EPR17876] – BSA and Azide free	Abcam	Cat#AB225896; RRID:AB_3076747
Alexa Fluor® 594 Anti-alpha smooth muscle Actin antibody [1A4]	Abcam	Cat#ab02368; RRID:AB_2924381
Mouse/Rat CD31/PECAM-1 Antibody	R&D Systems	Cat#AF3628; RRID:AB_2161028
Phospho-AMPKα (Thr172) (40H9) Rabbit mAb	Cell Signaling	Cat#2535; RRID: AB_331250
Tom20 (D8T4N) Rabbit mAb (HRP Conjugate)	Cell Signaling	Cat#72610; RRID:AB_2799825
LC3B (E5Q2K) Mouse mAb	Cell Signaling	Cat#83506; RRID: 2800018
GABARAP	Cell Signaling	Cat #13733; RRID: AB_2798306
p62	Cell Signaling	Cat#39749T; RRID: AB_2799160
PINK	Cell Signaling	Cat#6946S; RRID: AB_11179069
PARKIN	Cell Signaling	Cat#4211S; RRID: AB_2159920
VAMP8	Cell Signaling	Cat#13060; RRID: AB_2798103
RAB7	Cell Signaling	Cat#9367S; RRID: AB_1904103
LAMP2A	Invitrogen	Cat# 51-2200; RRID: AB_2533900
Total OXPHOS Human WB Antibody Cocktail	Abcam	Cat#ab110411; RRID: AB_2756818
Syntaxin 17 (D3D7H) Rabbit mAb	Cell Signaling	Cat#31261; RRID: AB_3076725
Phospho-NF-κB p65 (Ser536) (93H1)	Cell Signaling	Cat#3033; RRID: AB_331284
NF-κB p65 (L8F6) Mouse mAb	Cell Signaling	Cat#6956; RRID: AB_10828935
β-Actin (13E5) Rabbit mAb (HRP Conjugate)	Cell Signaling	Cat#5125; RRID: AB_1903890
14-3-3 γ (D15B7) Rabbit mAb	Cell Signaling	Cat#5522; RRID: AB_1082788
Anti-mouse IgG, HRP-linked Antibody	Cell Signaling	Cat#7076P2; RRID: AB_330924

Anti-rabbit IgG, HRP-linked Antibody	Cell Signaling	Cat#7074; RRID: AB_2099233
anti-TNFα (Adalimumab) antibody	Leinco Technologies	Cat# LT100-1MG; RRID: AB_2893873
Ultra-LEAF™ Purified Rat IgG1, κ Isotype Ctrl Antibody	Biolegend	Cat# 400458; RRID AB_11150233
Anti-TNF-α neutralizing antibody	Biolegend	Cat#506348; RRID: AB_2616672
CoraLite® Plus 647-conjugated LC3 Polyclonal antibody	Proteintech	Cat#CL647-14600; RRID: AB_2920226
**Chemicals, peptides, and recombinant proteins**		
Bovine-Lactadherin - FITC	HTI	Cat# NC0691117
Chrono-Par Thrombin	Chrono Log	Cat#NC9864726
Prostaglandin I2 (Sodium Salt) 5 mg	Cayman Chemicals	Cat#NC1135464
Chloroquine Diphosphate Salt	MP Biochemicals	Cat#193919
Ethyl (2-(5-Nitrothiophene-2-Carboxamido) Thiophene-3-Carbonyl) Carbamate (EACC)	MedChemExpress	Cat#HY-129111
Recombinant murine TNFα	Preprotech	Cat# 315-01A
Human Tumor Necrosis Factor-α (TNF)α	Sigma	Cat#t0157-100G
Human IL-6	Preprotech	Cat# 200-06-5UG
Human IL-1 beta (IL-1B)	Preprotech	Cat# 200-01B-2UG
Lipofectamine 3000	Invitrogen	Cat# L3000015
Propidium iodide	Abcam	Cat# ab14083
TRIzol	Invitrogen	Cat#15-596-018
Restore plus western blot stripping buffer, 500 mL	Thermo Scientific	Cat#46430
Precision Plus Protein All Blue Standard	Bio-Rad	Cat#1610373
GPRP	MedChemExpress	Cat#HY-P0074
Oligomycin A	Selleckchem	Cat#S1478
Rotenone	Selleckchem	Cat#S2348
Antimycin A	Millipore	Cat#A8674-25MG
Rapamycin	Selleckchem	Cat# S1039
Mito-TEMPO	Sigma Aldrich	Cat #SML0737
Apocynin	Cayman Chemical	Cat #11976
CellROX Green	Invitrogen	Cat# C10444
FCCP	Cayman Chemical	Cat#15218
**Critical commercial assays**		
PL Chip (20 chips) 40 assays	Diapharma	Cat#18002
PL Chip Reservoir 100 assays	Diapharma	Cat#18003
BABA Blood Tubes	Diapharma	Cat#18004
Stasys Platelet Card Set	Stasys	Cat#STC-004-V13-10
Pierce Rapid Gold BCA Protein Assay, 500 mL Kit	ThermoScientific	Cat#A53225

Criterion TGX Precast Gels (4-20%) 12, 18 and 26 wells	Bio-Rad	Cat# 5671093, 5671095, 5671094
Trans-Blot Turbo Midi 0.2 µm PVDF Transfer Packs	Bio-Rad	Cat#1704157
Seahorse XF Cell Mito Stress Kit	Agilent	Cat#103015-100, 103010-100
Seahorse XF24 FluxPak	Agilent	Cat#102340-100
Seahorse XF HS Mini FluxPak	Agilent	Cat#103724-100
RNeasy MinElute Cleanup Kit (50)	Qiagen	Cat# 74204
RNase-Free DNase Set (50)	Qiagen	Cat# 79254
NovaSeq 6000 S2 Reagent Kit v1.5 (100 cycles)	Illumina	Cat# 20028316
**Deposited data**		
Sequence data		GSE282993 ID:200282993
**Experimental models: Cell lines**		
Meg-01 cell line	ATCC	Cat# CRL-2021; RRID CVCL_0425
**Experimental models: Organisms/strains**		
Mouse: C57BL/6J	The Jackson Laboratory	Cat#:000664; RRID: IMSR JAX:000664
Mouse: B6.129S-Tnf^{tm2Gkl}/Flmg (TNFdARE)	Collins et al. (28)	IMSR: EM:04974
Mouse: C57BL/6-Tg(Pf4-icre)Q3Rsko/J	The Jackson Laboratory Tiedt et al. (29)	Cat #:008535 RRID: IMSR_JAX:008535
Mouse: C57BL/6JGpt-Stx17^{em1Cflox}/Gpt	GemPharmatech Xu et al. (30)	Cat#: T018448 MGI:1914977
**Oligonucleotides**		
Silencer Select Negative Control Sequence is not available due to vendor's policies	Ambion	Cat# 4390844
STX17 Silencer Pre-designed siRNA GGAUGACCUAGUACUUCUGtt	Ambion	Cat#AM16704
**Software and algorithms**		
GraphPad Software v9.5.1	Graphpad	www.graphpad.com
ImageJ	Schneider et al. (31)	https://imagej.net/ij/
DSPDA software v2.3	Nanostring (27, 32)	https://nanostring.com/
Enrichr	Xie et al. (25)	https://maayanlab.cloud/Enrichr/

R for Windows v4.3.1	RCore Team (17)	https://cran.r-project.org/bin/windows/base/old/4.3.1/
Custom computational pipeline for sequences	Baird et al. (22)	
gSNAP	Wu et al. (23)	
Cufflinks	Trapnell et al.(24)	
Ggplot2 package	Wickham (26)	https://cran.r-project.org/web/packages/ggplot2/index.html
Correlation Matrix-online Software: Analysis and Visualization	STHDA(33)	http://www.sthda.com/english/rsthda/correlation-matrix.php
EnrichR	Xie et al.(25)	https://maayanlab.cloud/Enrichr/
MetaboAnalyst v5.0	Pang et al.(34)	https://www.metaboanalyst.ca/
Agilent Seahorse Analytics Software v1.0.0-520	Agilent	https://www.agilent.com/en/product/cell-analysis/real-time-cell-metabolic-analysis/xf-software/agilent-seahorse-analytics-787485
Biorender	Biorender	https://app.biorender.com/

Supplementary Table 2. Key resources: antibodies, reagents, and software used.

	**Digital scanner**	
Protein	G: BBOX Chemi XX6	ImageQuant 350
LC3BI/II	Plts 2 min, Meg-01 1 min	Plts 4 min
TOM20	Plts 2 min, Meg-01 1 min	Plts 4 min
STX17	Plts 3 min, Meg-01 2 min	Plts 6 min
Mitochondrial Complexes	1.5 min low exposure<br>3 min high exposure	
p62	1 min	
PINK	1 min	
PARKIN	1 min	
VAMP8		3 min

RAB7		3 min
LAMP2A	2 min	

Supplementary Table 3. Optimized exposure times for protein detection by immunoblotting.

**Figure**	**Access link**
Figure 1A	https://BioRender.com/7ii2uh4
Figure 1E	https://BioRender.com/5uz248g
Figure 1K	https://BioRender.com/z4sbxfs
Figure 2D	https://BioRender.com/p48f215
Figure 2G	https://BioRender.com/8k0f5vn
Figure 3A	*https://BioRender.com/6tpgi5g*
Figure 3I	*https://BioRender.com/l74g536*
Figure 3M	https://BioRender.com/slzgbt8
Figure 4A	https://BioRender.com/sbouros
Figure 4B	https://BioRender.com/tzompdp
Figure 4H	https://BioRender.com/7ovcqra
Figure 4I	*https://BioRender.com/1802kf8*
Figure 4J	https://BioRender.com/1802kf8
Figure 5A	https://BioRender.com/avehi3l
Figure 5F	https://BioRender.com/ifb5te8
Figure 5K	*https://BioRender.com/xwwr291*
Figure 6A Mouse	https://BioRender.com/6mb9f85
Figure 6A pathways	https://BioRender.com/bwfx590
Figure 6D	https://BioRender.com/6kfxaot
Figure 7A	https://BioRender.com/tfethhr
Supplementary Figure 2E	https://BioRender.com/l39m4rw
Supplementary Figure 3E	https://BioRender.com/50a1371
Supplementary Figure 5G	https://BioRender.com/f8kai43
Supplementary Figure 5H	https://BioRender.com/jvpiie7
Graphical Abstract	https://BioRender.com/f6kqogb

Supplementary Table 4. List of URL links to images created in Biorender

Supplementary Figure 1.

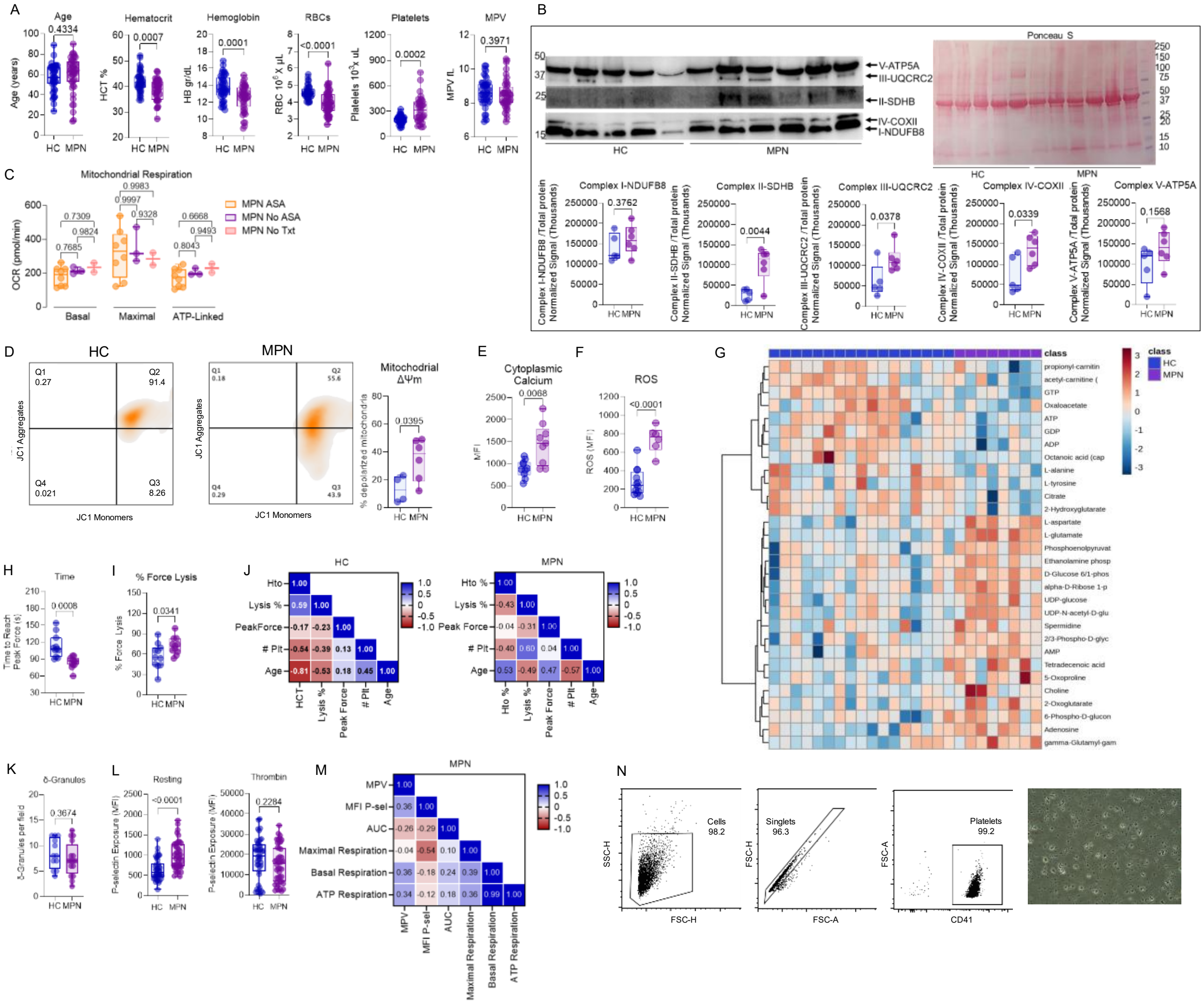

Supplementary Figure 1. Relevant metabolic and clinical data of HCs and patients with MPN. (**A**) Hematological profiles of healthy controls (HCs, *n*= 43) and patients with JAK2 V617F polycythemia vera (MPN) (*n*= 33); Mann–Whitney Test or unpaired t-tests were performed. **(B)** Immunoblot analysis of key components of the mitochondrial electron transport chain complexes in platelets from HCs (*n*= 5) and MPN patients (*n*= 6); unpaired t test. (**C**) Subgroup analysis of the platelet oxygen consumption rate (OCR) in patients with MPN receiving aspirin (ASA) treatment (from Fig. 1 B and C, *n*= 13); Brown-Forsythe and Welch ANOVA test and Dunnett's T3 multiple comparison test. (**D**) JC-1 analysis of the mitochondrial membrane potential in platelets from HCs (*n*= 4) and MPN patients (*n*= 6); unpaired t test. **(E)** Cytoplasmic calcium levels of platelets from HCs (*n*= 10) and MPN patients (*n*= 9); unpaired t test with Welch's correction. **(F)** Reactive oxygen species (ROS) level in platelets from HCs (*n*= 12) and MPN patients (*n*= 6); unpaired t test. **(G)** Heatmap of the top 30 differentially identified metabolites in platelets, HC (*n*= 18), and MPN platelets (*n*= 8); unpaired t test. **(H)** Time to reach peak force and (**I**) percentage of force lysis from the platelet force strength assay, HCs (*n*= 11) and MPN patients (*n*= 11); unpaired t test**,** or unpaired t test with Welch's correction, respectively. **(J)** Correlation analyses between the hematological parameters, the peak force and % of force lysis. (**K**) δ-Granule quantification

by electron microscopy; unpaired t test. **(L**) P-selectin exposure in resting and thrombin-activated washed platelets from HCs (*n*= 42) and MPN patients (*n*= 44); Mann–Whitney test. (**M**) Correlation analyses between mitochondrial respiration parameters, mean platelet volume (MPV), and P-selectin levels after activation (P-selectin MFI). (**N**) Flow cytometry gating strategy for assessing platelet purity and representative photograph of platelet-rich plasma. Box plots represent sample distributions unless otherwise noted. Box plots (A, B, C, D, E, F, H, I, K, and L) represent the data distributions.

Supplementary Figure 2.

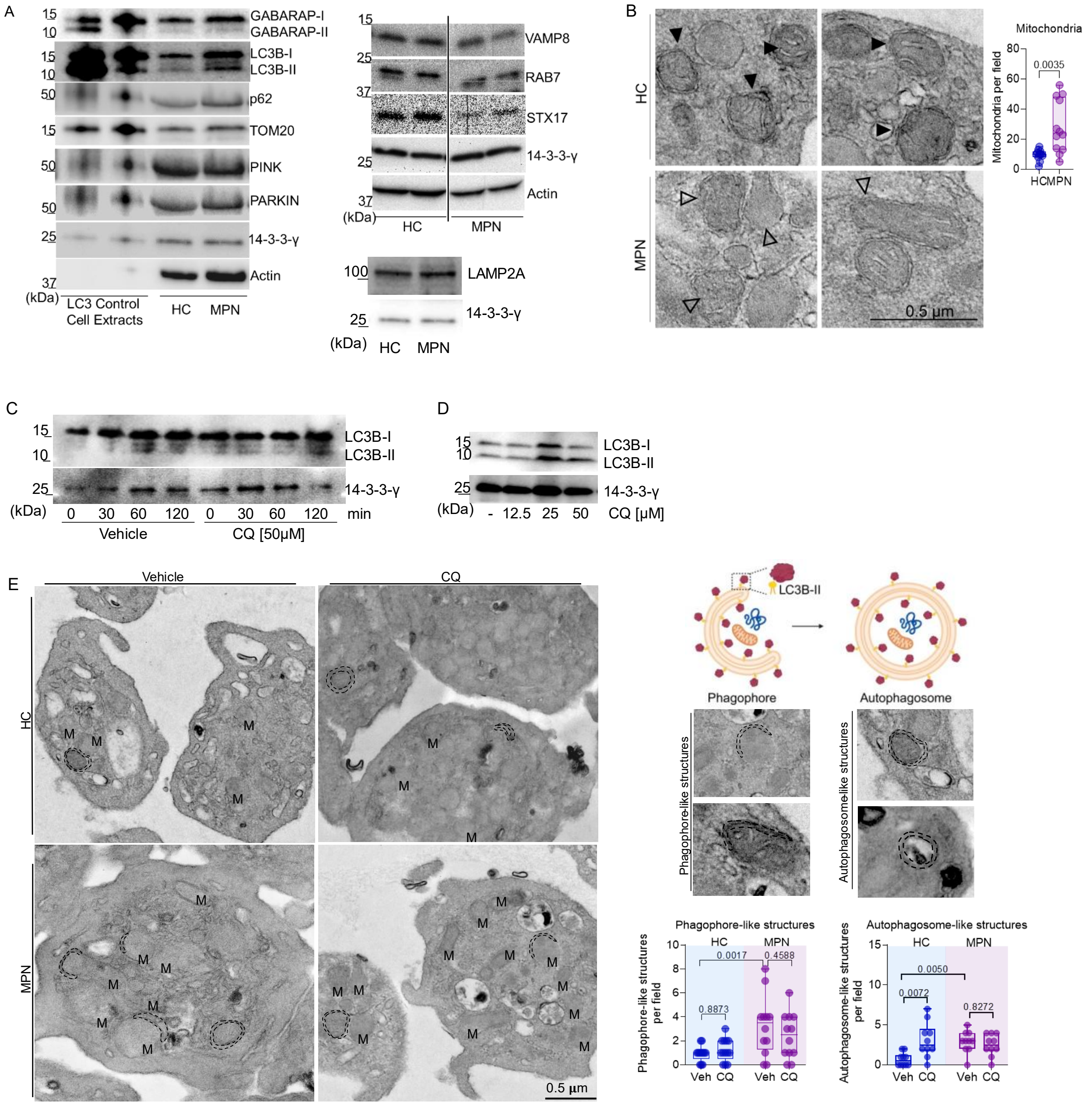

Supplementary Figure 2. MPN platelets contain key autophagic machinery and dysmorphic mitochondria. (**A**) Immunoblots showing the expression of proteins involved in autophagy and mitophagy pathways (GABARAP, LC3B, p62, TOM20, PINK, and PARKIN) and mediators of autophagosome–lysosome fusion (VAMP8, RAB7, STX17) in platelets from HCs and MPN patients. All proteins in each panel were probed from the same blot, with 14-3-3-γ and or actin as loading controls. Lanes were run on the same gel but were noncontiguous. (**B**) Representative transmission electron microscopy (TEM) images of pooled platelets (pooled from 5 individuals per group) from HCs and MPN patients. The black arrows indicate intact mitochondria with double membranes and normal cristae. The empty arrows indicate mitochondria with dysmorphic features, including disrupted cristae, discontinuous outer membranes, and swelling. The mitochondria were

identified and quantified; unpaired t test with Welch's correction. (**C**) Immunoblot of a kinetic assay quantifying LC3B-II accumulation in platelets treated with vehicle (PBS) or chloroquine (CQ, 50 µM). (**D**) Immunoblot analysis of the effects of different CQ concentrations (12.5, 25, and 50 µM) on optimal LC3B-II accumulation in platelets treated for 2 hours. (**E**) TEM images of HC and MPN platelets (pooled from 5 individuals per group) treated with CQ for 2 hours. Autophagy-related structures, including phagophore-like-structures (cup-shaped double membrane structures) and autophagosome-like structures (double-membrane vesicles), are highlighted with dashed lines. The right panel shows examples of autophagic structures used for quantification; two-way ANOVA with Šídák's multiple comparisons test. Box plots (B, E) represent the data distribution.

Supplementary Figure 3.

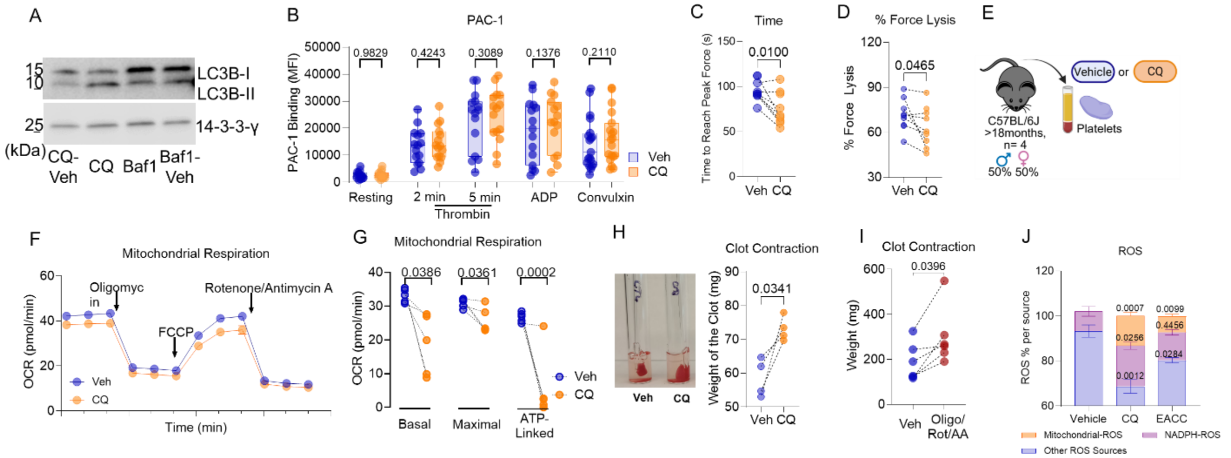

Supplementary Figure 3. Functional and metabolic effects of the pharmacological autophagy inhibition in human and murine platelets. (**A**) Comparison of the use of CQ and Bafilomycin 1 (Baf1) to measure the autophagic flux of platelets. Platelets from HCs were incubated with CQ-vehicle (PBS), Baf1-vehicle (DMSO), CQ (50 µM), or Baf1 (100 nM) for 2 hours. (**B**) Human platelets from HCs were treated with vehicle (PBS) or CQ (50 µM) for 2 hours. Platelet activation was analyzed by flow cytometry at baseline and upon activation with thrombin, ADP, and convulxin; box plots illustrate the sample distribution; paired t test. **(C)** Time to reach peak force and (**D)** percentage of force lysis from the platelet force strength assay, HC (*n*= 11) and MPN (*n*= 11); before-and-after graph; paired t test. **(E)** Experimental design (**F**) The OCR of washed platelets (*n*= 5) was measured with the Seahorse XF HS Mini Analyzer (mean ± SEM of three independent samples). **(G)** Basal, maximal, and ATP-linked respiration data. The before-and-after graph shows the effects of pharmacological inhibition of autophagy with CQ (*n*= 5); paired t test. **(H)** Thrombin-induced clot contraction assay using normalized platelet counts (*n*= 4); before-and-after-graph, paired t test. (**I**) HC platelets were incubated for 2 hours with vehicle or a combination of oligomycin (1.5 µM), rotenone (0.5 µM), and antimycin A (0.5 µM). Thrombin-induced clot contraction assay using normalized platelet counts (*n*= 6); before-and-after graphs; paired t test. (J) Platelets from human donors were pre-treated either with mito-TEMPO (20 µM) or apocynin (300 µM) for 1 hour, then autophagy was inhibited with CQ (25 µM) or EACC (5 µM) for 2 hours. ROS were measured by flow cytometry using Mito-Sox staining. One-way ANOVA, Sidak post-test.

Supplementary Figure 4.

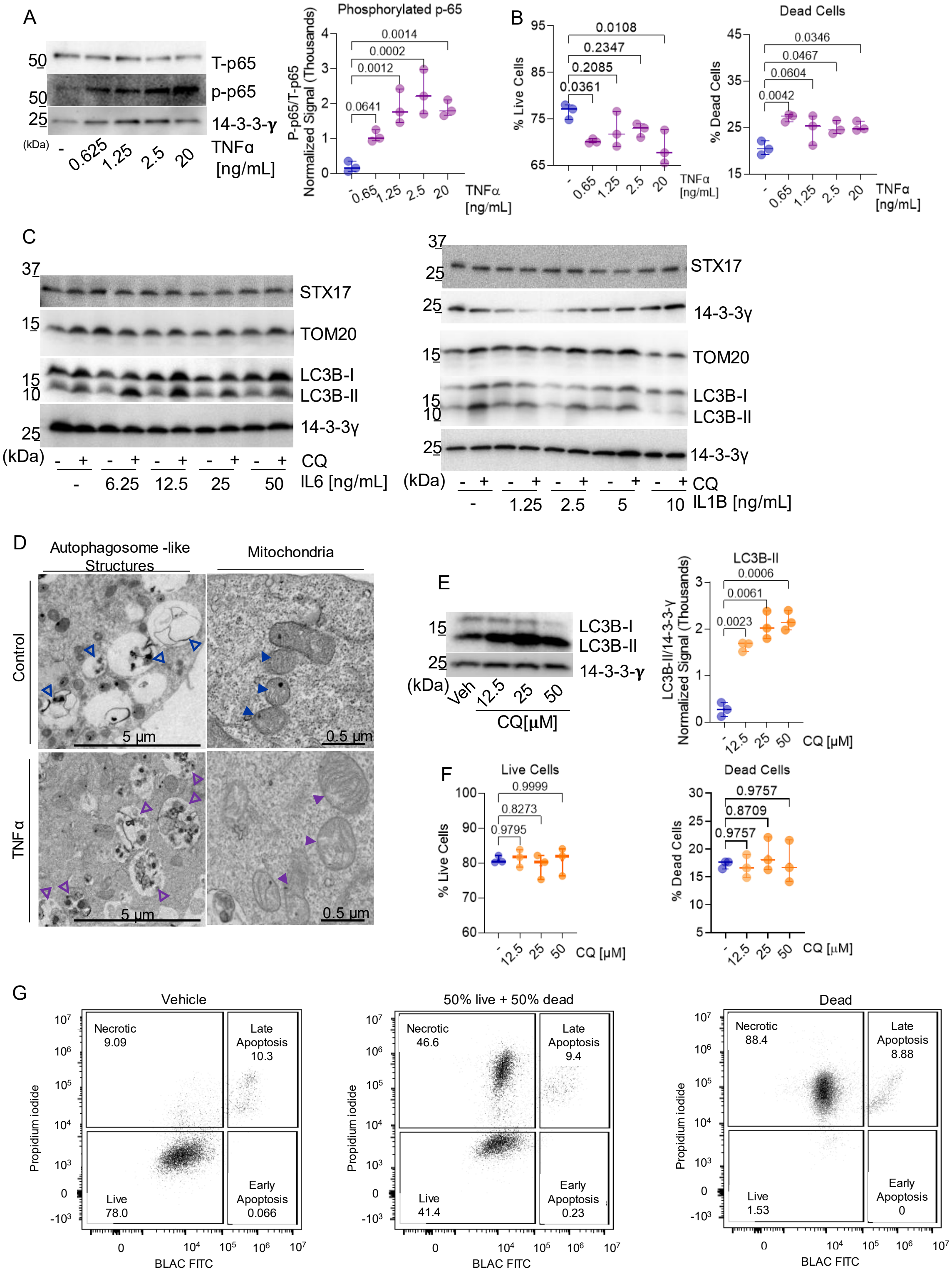

Supplementary Figure 4. Pharmacological inhibition of autophagy decreases mitochondrial respiration in Meg-01 cells. (A) Immunoblot of phosphorylated and total p65 from Meg-01 cells

treated with vehicle (0.01% BSA in PBS or different doses of TNFα (0.65, 1.25, 2.5, 20 ng/mL) for 72 h (*n*= 3 per condition); one-way ANOVA, Dunnett post-test. (**B)** Live and dead cell analysis of Meg-01 cells treated for 72 hours with vehicle (0.01% BSA in PBS) or TNFα at doses of 0.65, 1.25, 2.5, 20 ng/mL (*n*= 3 per condition); one-way ANOVA, post-hoc test. **(C)** Immunoblot analysis of the STX17, LC3B-II, and TOM20 proteins in Meg-01 cells treated with vehicle (PBS-0.01% albumin), IL-6 (6.25, 12.5, 25, or 50 ng/mL), or IL-1B (1.15, 2.5, 5, or 10 ng/mL) for 72 hours. The cells were subsequently treated with CQ (25 µM) or vehicle (PBS) for 4 hours. (**D**) Representative TEM images of pooled samples from 4 independent passages. The blue arrows indicate spheroid-shaped healthy mitochondria, whereas the purple arrows indicate swollen mitochondria with disrupted cristae. The empty blue arrows highlight the autophagosome-like structures, and the empty purple arrows denote the highly dense content of the autophagosome-like structures. **(E)** Immunoblot analysis of LC3B-II accumulation in Meg-01 cells treated with vehicle (PBS) or CQ at doses of 12.5, 25, 50 µM for 4 hours (*n*= 3 per condition); one-way ANOVA with Tukey's post hoc test. **(F)** Analysis of live and dead cells that were treated with vehicle (PBS) or different concentrations of CQ (12.5, 25, or 50 µM) for 4 hours (*n*= 3 each); one-way ANOVA with Tukey's post hoc test. **(G)** Gating strategy for analyzing the viability of Meg-01 cells. Box plots (A, B, E) represent the data distribution.

Supplementary Figure 5.

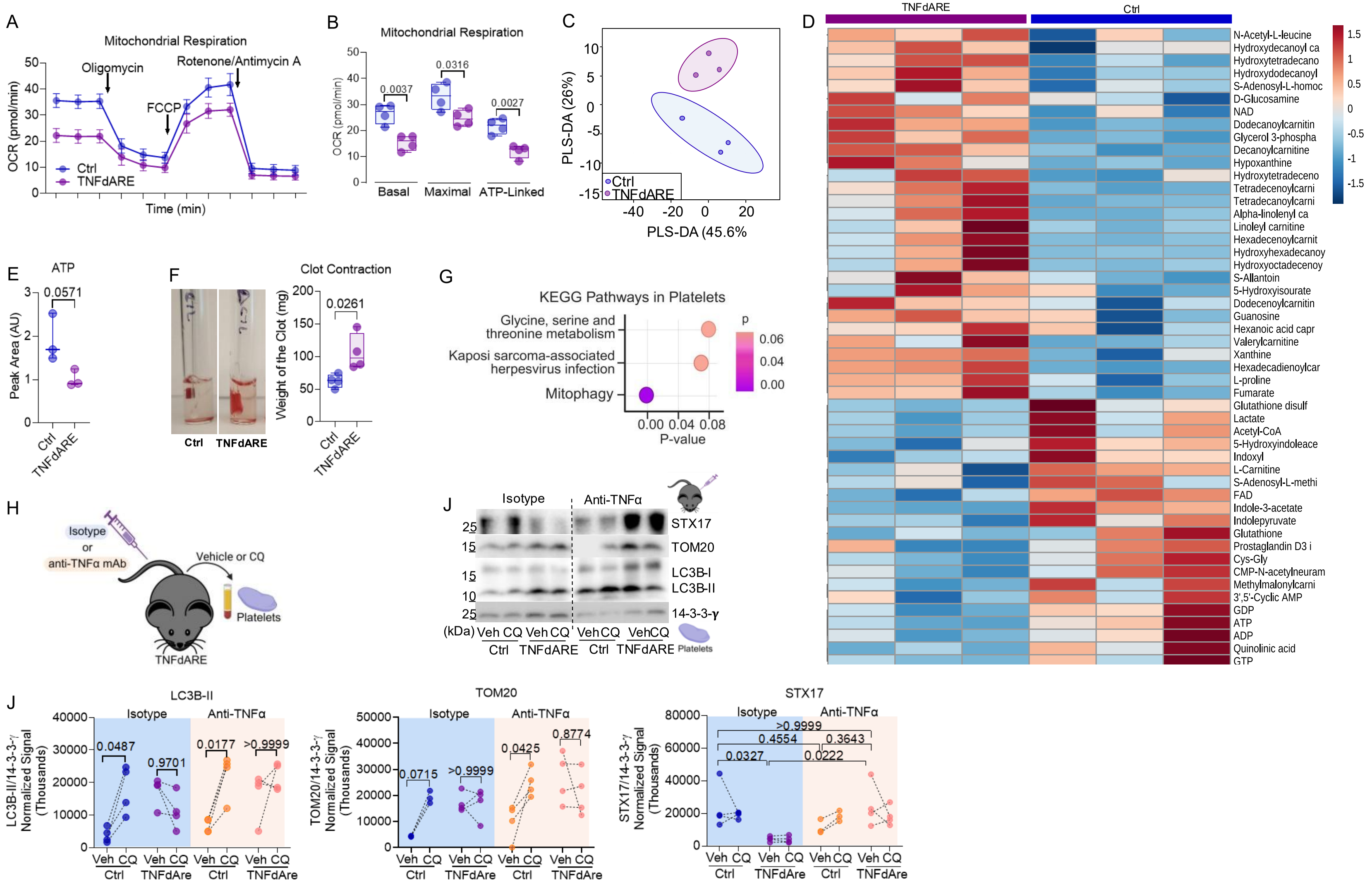

Supplementary Figure 5. Platelets from TNFdARE mice exhibit metabolic and hemostatic defects similar to those observed in MPN platelets. (A) The OCR of washed platelets (*n*= 4 per group) was measured with the Seahorse XF HS Mini Analyzer (mean ± SEM of four independent samples). **(B)** Basal, maximal, and ATP-linked respiration (*n*= 4 each); unpaired t test. **(C)** Partial least squares discriminant analysis (PLS-DA) from unbiased metabolome analysis using a UHPLC-MS system from washed platelets (*n*= 3 each group). **(D)** Heatmap showing the top abundant metabolites, unpaired t-test. **(E)** ATP metabolite levels from the metabolome assay (*n*= 3); unpaired t test. **(F)** Thrombin-induced clot contraction assay (*n*= 4 each group); before-and-after graph; unpaired t-test. **(G)** Differentially expressed KEGG pathways (*n*= 4 each). **(H)** Experimental design: Ctrl and TNFdARE mice (*n*= 4 per group) were treated every other day with IP injections of isotype control antibody at 10 µg/g of body weight or anti-TNFα neutralizing antibody at 10 µg/g body weight for 20 days. Platelets were treated with vehicle (PBS) or CQ (50 µM) for 2 hours. (**I**) Immunoblot analysis of LC3B, TOM20, and STX17 levels in platelets from Ctrl and TNFdARE mice treated as described in (**H**). The before and after graph illustrates the effect of the vehicle/CQ-treatment of each group, one-way ANOVA with Tukey's post-hoc test. The immunoblot lanes were run on the same gel but were noncontiguous. Box plots (B, E, F) represent the data distribution.

Legends for videos

Supplementary video 1. Platelet contractile forces of HC platelets. The platelet strength assay (PSA) was performed using the ATLAS system to measure the force exerted by platelets during clot contraction. The sensors quantified the contraction force exerted by HC platelets during the initial minutes of clot formation (4).

Supplementary video 2. MPN platelets exhibit impaired contractile forces. The PSA was performed using the ATLAS system to measure the force exerted by platelets during clot contraction. Compared with HC platelets, MPN platelets demonstrate a reduced contraction force during the initial minutes of clot formation.

References

1. Feng W, Chang C, Luo D, Su H, Yu S, Hua W, et al. Dissection of autophagy in human platelets. *Autophagy.* 2014;10(4):642-51.
2. Wang C-Y, Ma S, Bi S-J, Su L, Huang S-Y, Miao J-Y, et al. Enhancing autophagy protects platelets in immune thrombocytopenia patients. *Annals of Translational Medicine.* 2019;7(7):134.
3. Vats S, and Manjithaya R. A reversible autophagy inhibitor blocks autophagosome-lysosome fusion by preventing Stx17 loading onto autophagosomes. *Mol Biol Cell.* 2019;30(17):2283-95.
4. Ting LH, Feghhi S, Taparia N, Smith AO, Karchin A, Lim E, et al. Contractile forces in platelet aggregates under microfluidic shear gradients reflect platelet inhibition and bleeding risk. *Nature Communications.* 2019;10(1):1204.
5. Esparza O, Hernandez G, Rojas-Sanchez G, Calzada-Martinez J, Nemkov T, Kelher M, et al. Platelets from blood diversion pouches (DPs) are a suitable alternative for functional, bioenergetic, and metabolomic analyses. *Blood Transfus.* 2023.
6. Tucker KL, Sage T, and Gibbins JM. Clot retraction. *Methods Mol Biol.* 2012;788:101-7.
7. Klionsky DJ, Abdel-Aziz AK, Abdelfatah S, Abdellatif M, Abdoli A, Abel S, et al. Guidelines for the use and interpretation of assays for monitoring autophagy (4th edition)(1). *Autophagy.* 2021;17(1):1-382.
8. Ylä-Anttila P, Vihinen H, Jokitalo E, and Eskelinen EL. Monitoring autophagy by electron microscopy in Mammalian cells. *Methods Enzymol.* 2009;452:143-64.
9. Jung M, Choi H, and Mun JY. The autophagy research in electron microscopy. *Appl Microsc.* 2019;49(1):11.
10. Eskelinen E-L. To be or not to be? Examples of incorrect identification of autophagic compartments in conventional transmission electron microscopy of mammalian cells. *Autophagy.* 2008;4(2):257-60.
11. Eskelinen E-L. In: Deretic V ed. *Autophagosome and Phagosome*. Totowa, NJ: Humana Press; 2008:11-28.
12. Neumüller J, Ellinger A, and Wagner T. Transmission Electron Microscopy of Platelets from Apheresis and Buffy-Coat-Derived Platelet Concentrates. *InTech.* 2015.
13. White JG. Electron microscopy methods for studying platelet structure and function. *Methods Mol Biol.* 2004;272:47-63.
14. White JG, and Krumwiede M. Some contributions of electron microscopy to knowledge of human platelets. *Thromb Haemost.* 2007;98(1):69-72.
15. White JG. Electron opaque structures in human platelets: which are or are not dense bodies? *Platelets.* 2008;19(6):455-66.
16. Nemkov T, Hansen KC, and D'Alessandro A. A three-minute method for high-throughput quantitative metabolomics and quantitative tracing experiments of central carbon and nitrogen pathways. *Rapid Commun Mass Spectrom.* 2017;31(8):663-73.
17. Team RC. Vienna, Austria: R Foundation for Statistical Computing; 2021.
18. Baumgartner R, Umlauf E, Veitinger M, Guterres S, Rappold E, Babeluk R, et al. Identification and validation of platelet low biological variation proteins, superior to GAPDH, actin and tubulin, as tools in clinical proteomics. *J Proteomics.* 2013;94:540-51.
19. Lomnytska M, Pinto R, Becker S, Engström U, Gustafsson S, Björklund C, et al. Platelet protein biomarker panel for ovarian cancer diagnosis. *Biomarker Research.* 2018;6(1):2.

20. Schmidt GJ, Reumiller CM, Ercan H, Resch U, Butt E, Heber S, et al. Comparative proteomics reveals unexpected quantitative phosphorylation differences linked to platelet activation state. *Scientific Reports.* 2019;9(1):19009.
21. Invitrogen. Normalization in western blotting to obtain relative quantitation. https://assets.thermofisher.com/TFS-Assets/BID/Technical-Notes/ibright-normalization-western-blotting-relative-quantitation-technical-note.pdf. Accessed 01/01/2022.
22. Baird NL, Bowlin JL, Cohrs RJ, Gilden D, and Jones KL. Comparison of varicella-zoster virus RNA sequences in human neurons and fibroblasts. *J Virol.* 2014;88(10):5877-80.
23. Wu TD, and Nacu S. Fast and SNP-tolerant detection of complex variants and splicing in short reads. *Bioinformatics.* 2010;26(7):873-81.
24. Trapnell C, Williams BA, Pertea G, Mortazavi A, Kwan G, van Baren MJ, et al. Transcript assembly and quantification by RNA-Seq reveals unannotated transcripts and isoform switching during cell differentiation. *Nat Biotechnol.* 2010;28(5):511-5.
25. Xie Z, Bailey A, Kuleshov MV, Clarke DJB, Evangelista JE, Jenkins SL, et al. Gene Set Knowledge Discovery with Enrichr. *Curr Protoc.* 2021;1(3):e90.
26. Wickham H. ggplot2: Elegant Graphics for Data Analysis. 2016.
27. NanoString Technologies I. *Automated Slide Preparation.* Online: NanoString Technologies, Inc.; 2023.
28. Collins CB, Strassheim D, Aherne CM, Yeckes AR, Jedlicka P, and de Zoeten EF. Targeted inhibition of heat shock protein 90 suppresses tumor necrosis factor-α and ameliorates murine intestinal inflammation. *Inflamm Bowel Dis.* 2014;20(4):685-94.
29. Tiedt R, Schomber T, Hao-Shen H, and Skoda RC. Pf4-Cre transgenic mice allow the generation of lineage-restricted gene knockouts for studying megakaryocyte and platelet function in vivo. *Blood.* 2007;109(4):1503-6.
30. Xu H, Yu W, Sun M, Bi Y, Wu NN, Zhou Y, et al. Syntaxin17 contributes to obesity cardiomyopathy through promoting mitochondrial Ca2+ overload in a Parkin-MCUb-dependent manner. *Metabolism - Clinical and Experimental.* 2023;143.
31. Schneider CA, Rasband WS, and Eliceiri KW. NIH Image to ImageJ: 25 years of image analysis. *Nature Methods.* 2012;9(7):671-5.
32. Goytain A, and Ng T. In: Li H, and Elfman J eds. *Chimeric RNA: Methods and Protocols*. New York, NY: Springer US; 2020:125-39.
33. STHDA. Correlation Matrix-online Software: Analysis and Visualization. http://www.sthda.com/english/rsthda/correlation-matrix.php. Accessed 10/2023, 2023.
34. Pang Z, Zhou G, Ewald J, Chang L, Hacariz O, Basu N, et al. Using MetaboAnalyst 5.0 for LC–HRMS spectra processing, multi-omics integration and covariate adjustment of global metabolomics data. *Nature Protocols.* 2022;17(8):1735-61.
