# Supplementary material for: TNF-α impairs platelet function by inhibiting autophagy and disrupting metabolism via syntaxin 17 downregulation
Source: J Clin Invest. 2025 Jun 10;135(15):e186065. doi: 10.1172/JCI186065 (PMC12321402; doi:10.1172/JCI186065)

Supplementary File 2

Unedited Western Blots

Figure 2B

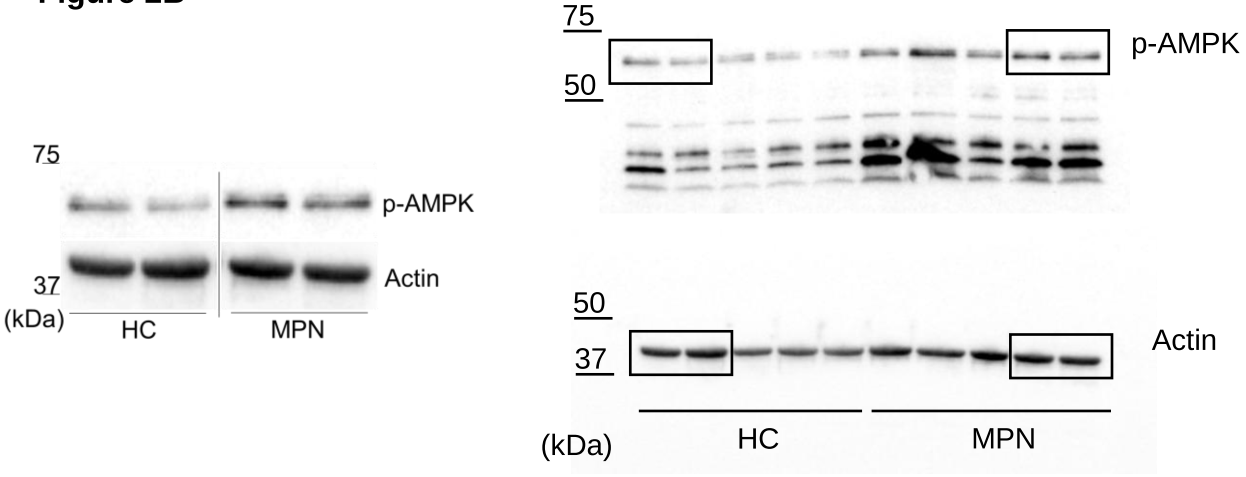

Figure 2E

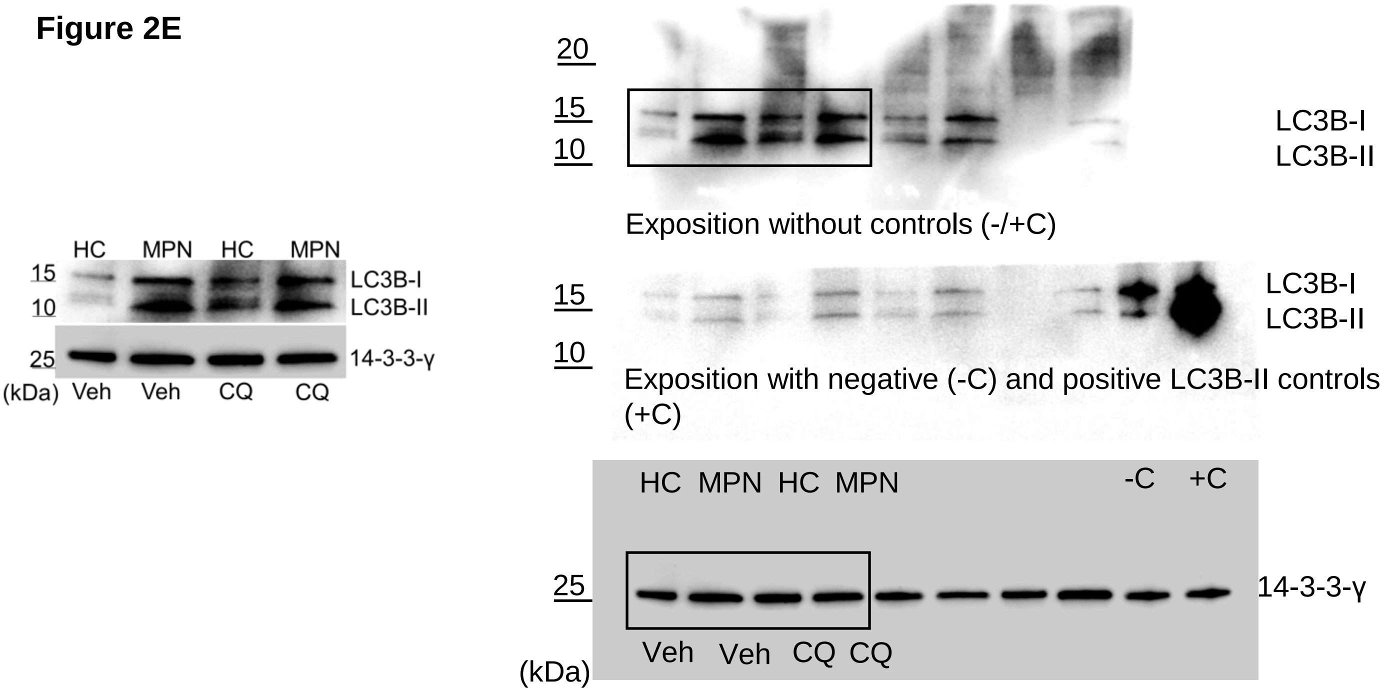

Figure 2F

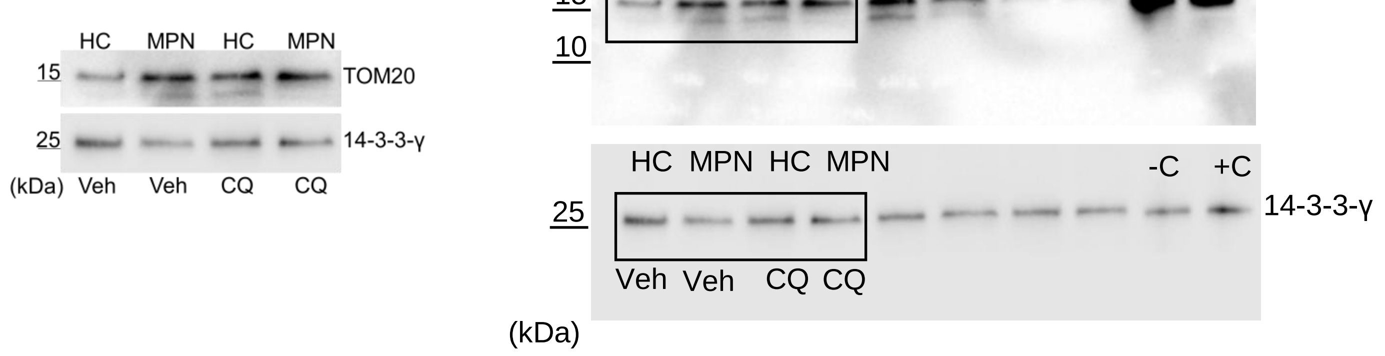

-C and +C are negative and positive autophagic controls

Figure 4A

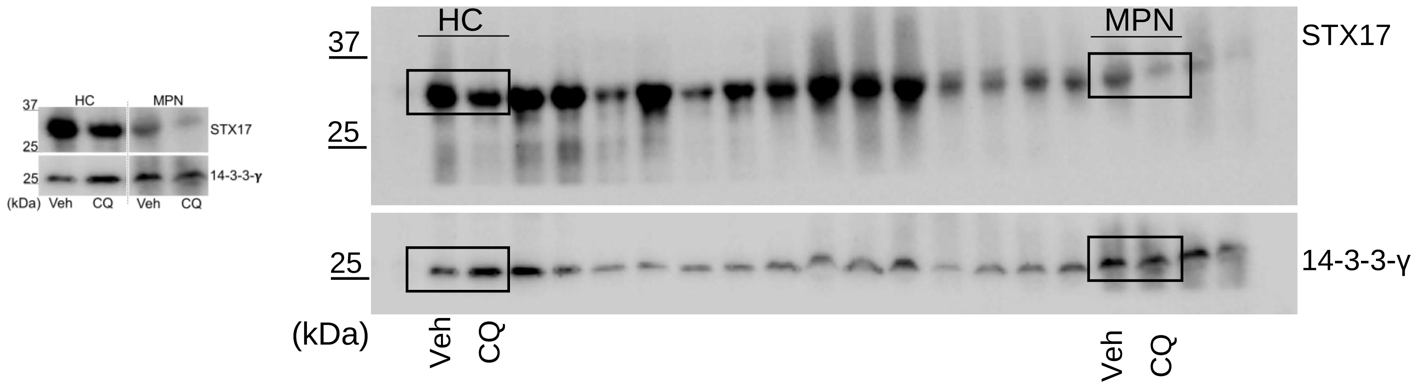

Figure 4C

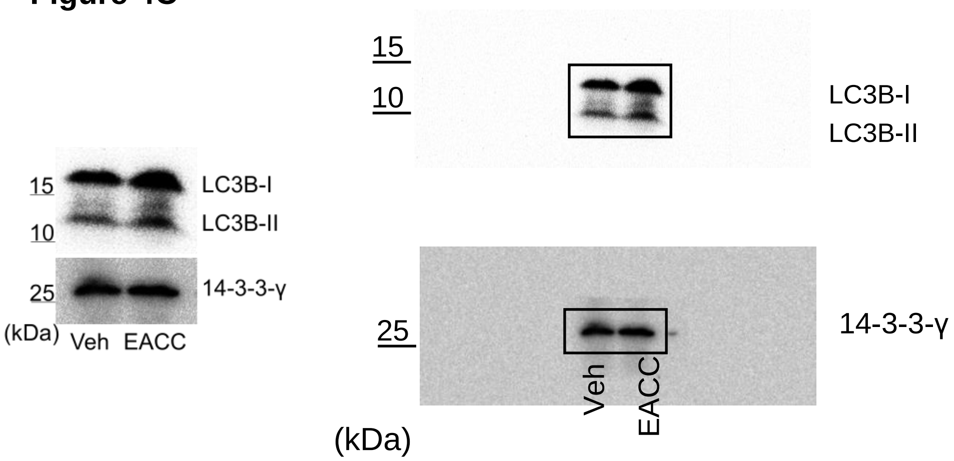

Figure 4D

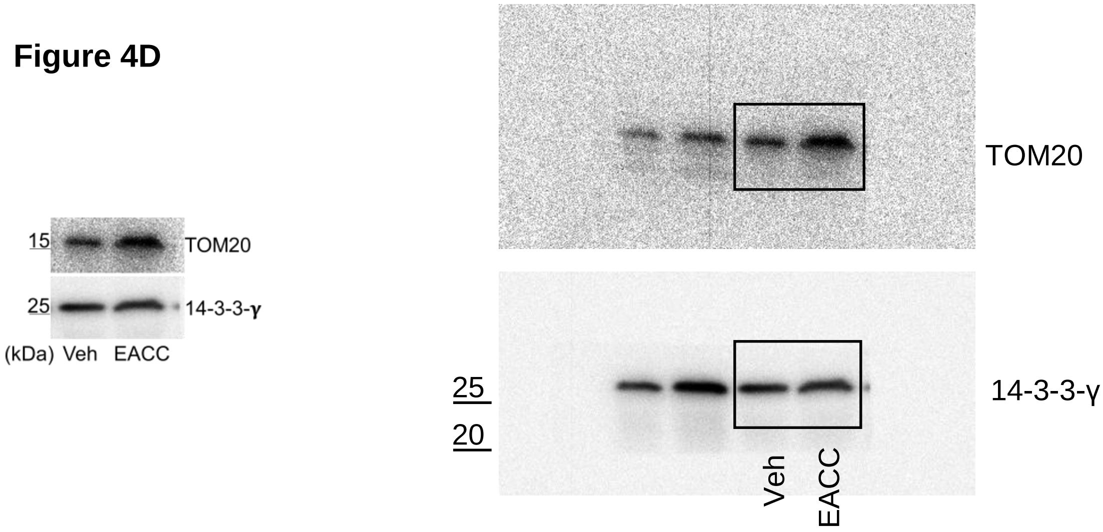

Figure 4J

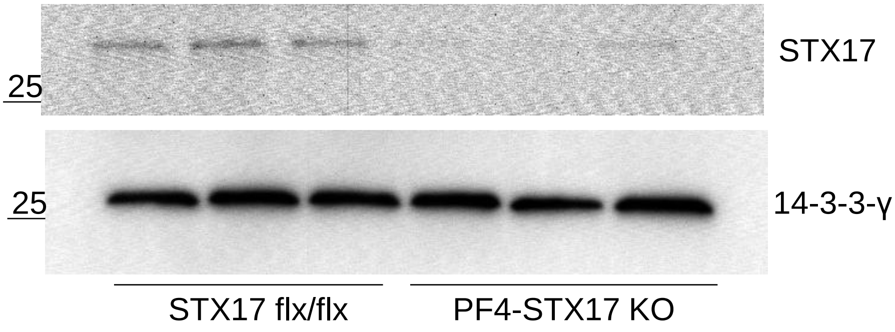
25
STX17
25
14-3-3-γ
STX17 flx/flx
PF4-STX17 KO

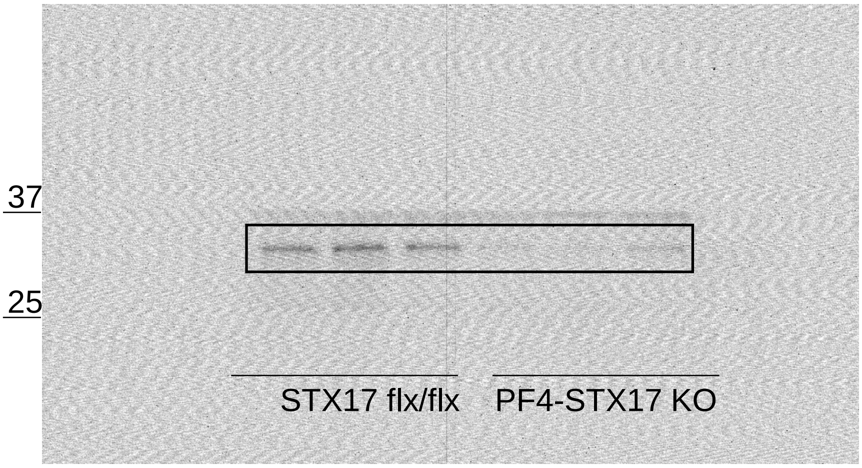
37
25
STX17 flx/flx
PF4-STX17 KO

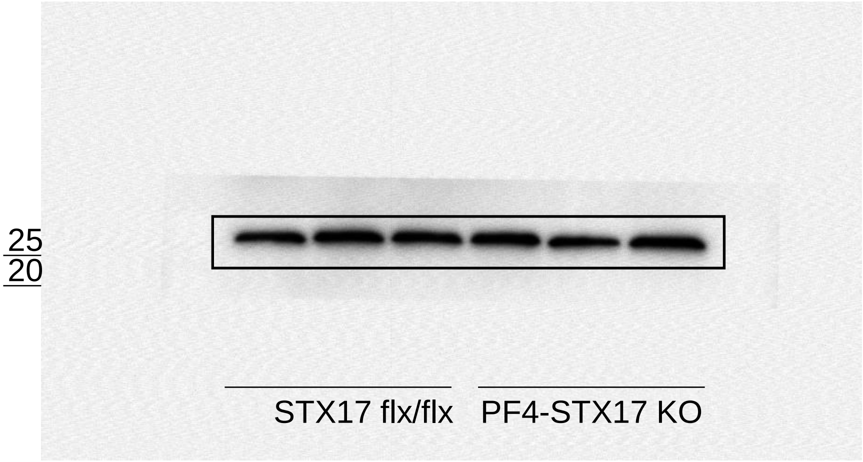
25
20
STX17 flx/flx
PF4-STX17 KO

Figure 5B

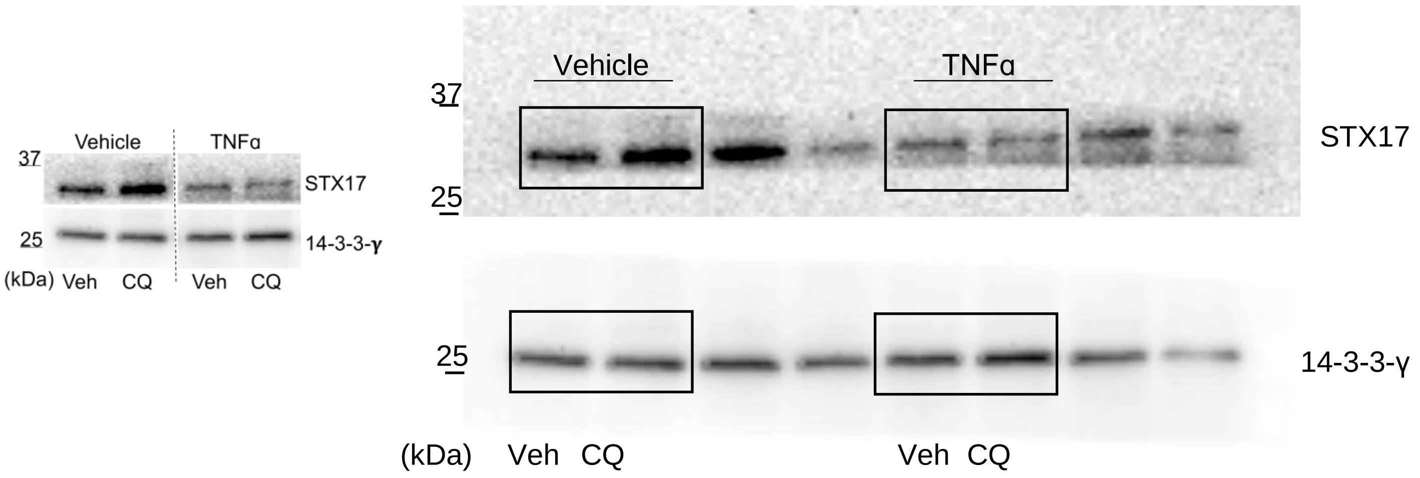

Figure 5C

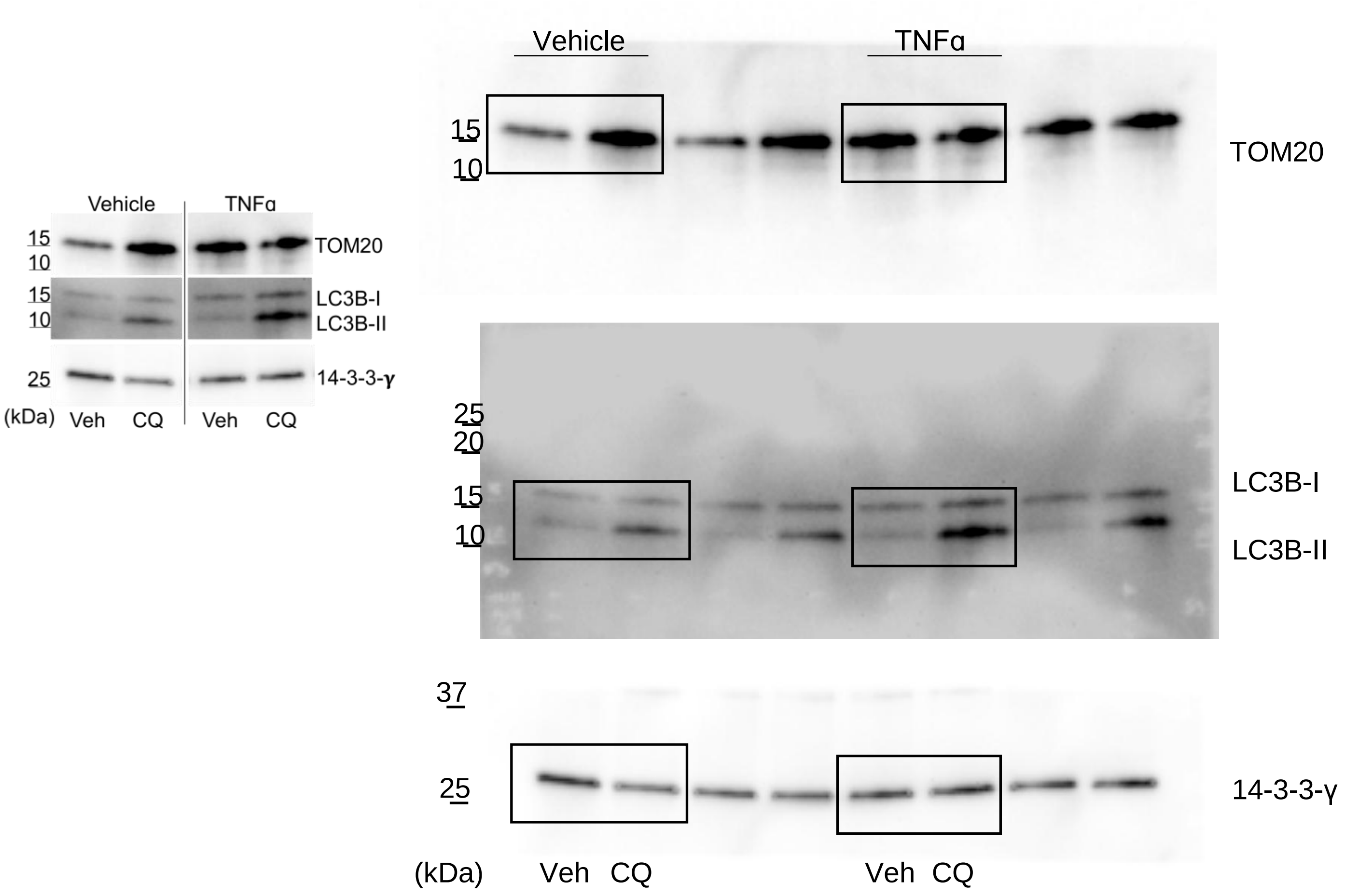

Figure 5G

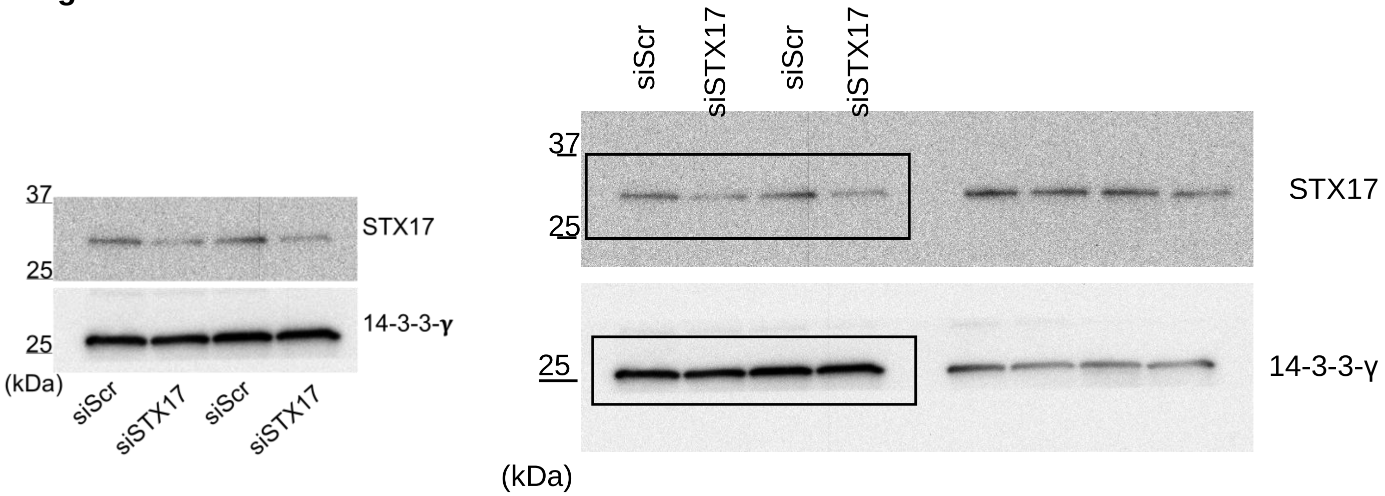

Figure 5H

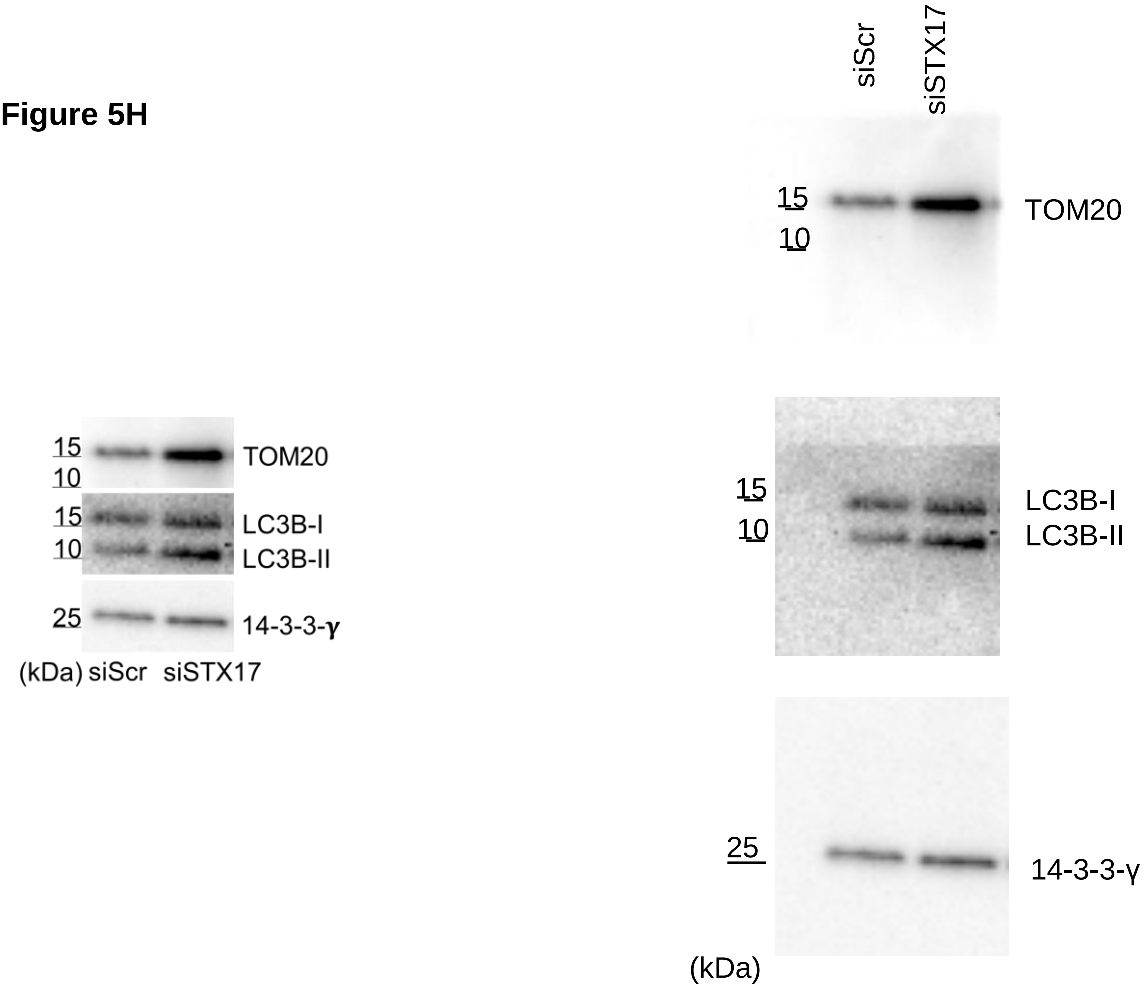

Figure 6B

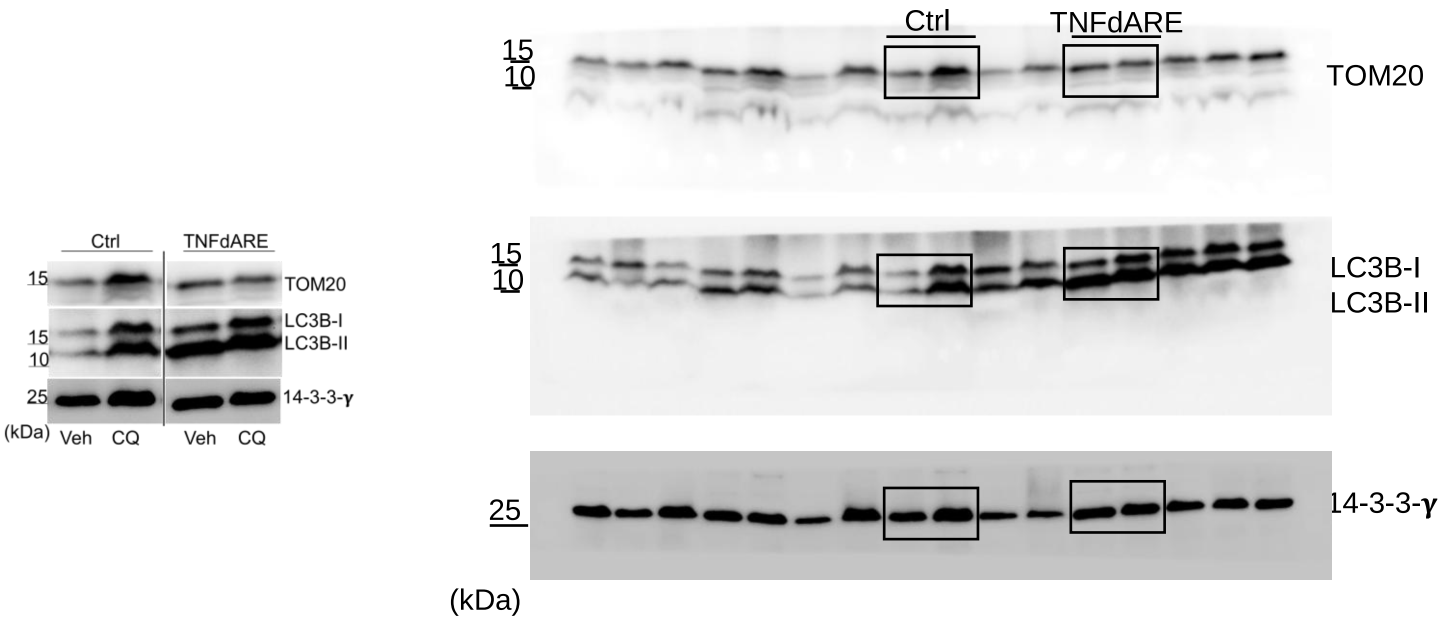

Figure 6C

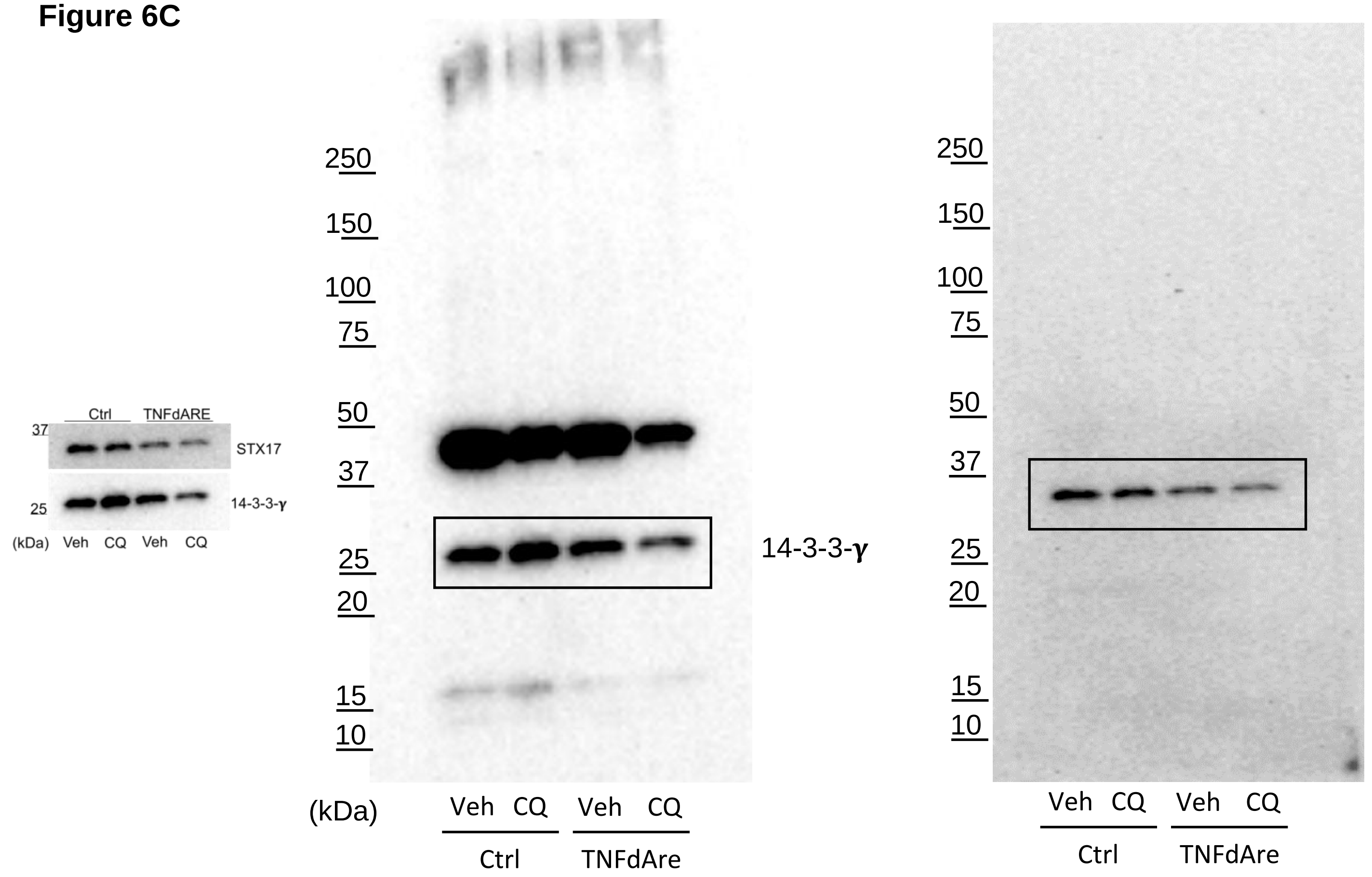

Figure 6D

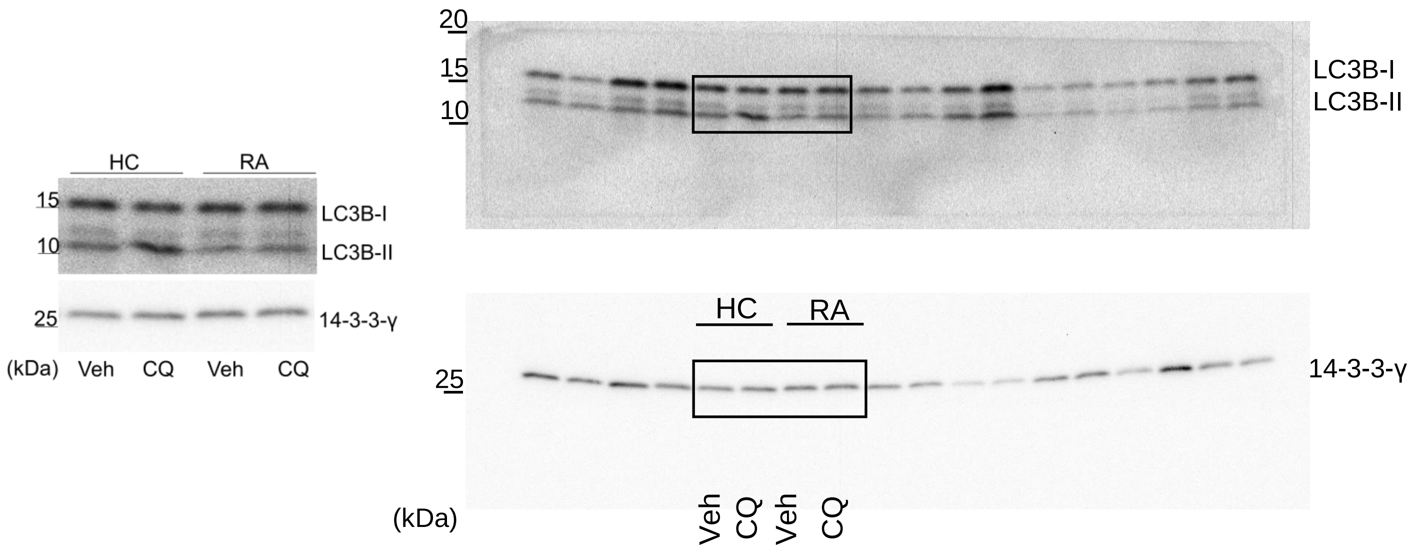

Figure 6E

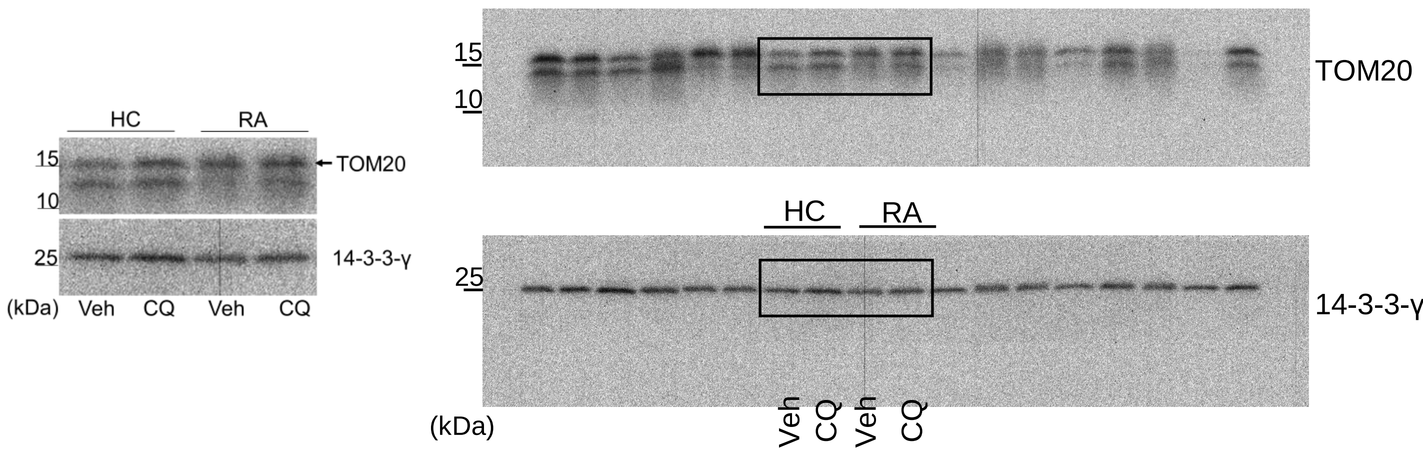

Figure 7B

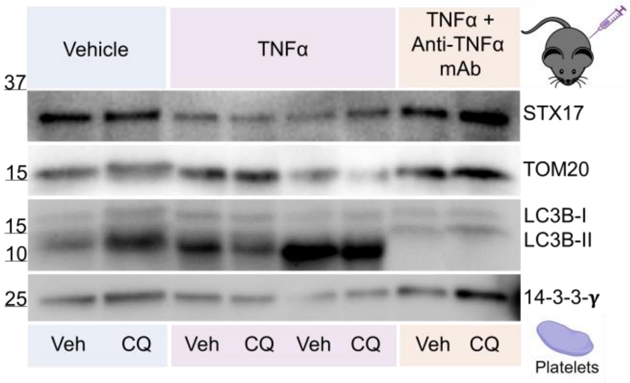
Vehicle
TNFα
TNFα + Anti-TNFα mAb
37
STX17
15
TOM20
15
10
LC3B-I
LC3B-II
25
14-3-3-γ
Veh
CQ
Veh
CQ
Veh
CQ
Veh
CQ
Platelets

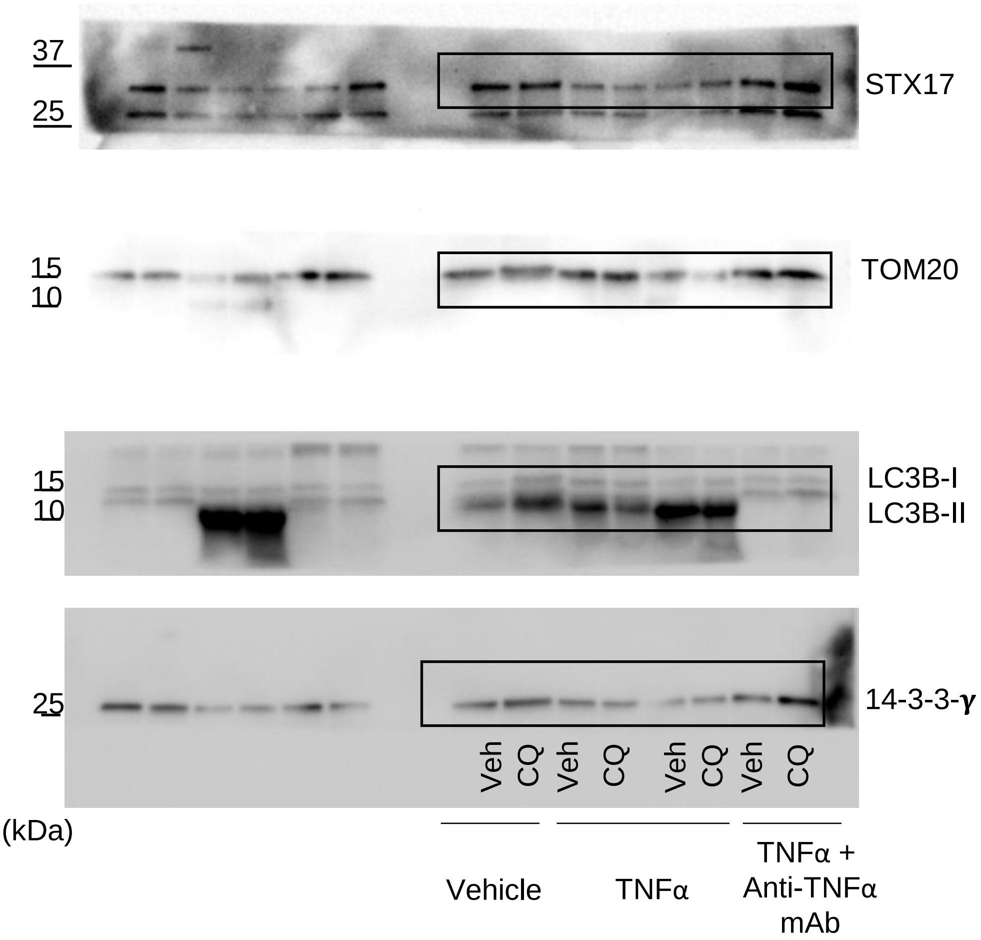
37
25
STX17
15
10
TOM20
15
10
LC3B-I
LC3B-II
25
14-3-3-γ
Veh
CQ
Veh
CQ
Veh
CQ
Veh
CQ
(kDa)
Vehicle
TNFα
TNFα + Anti-TNFα mAb

Supplementary Figure 1B

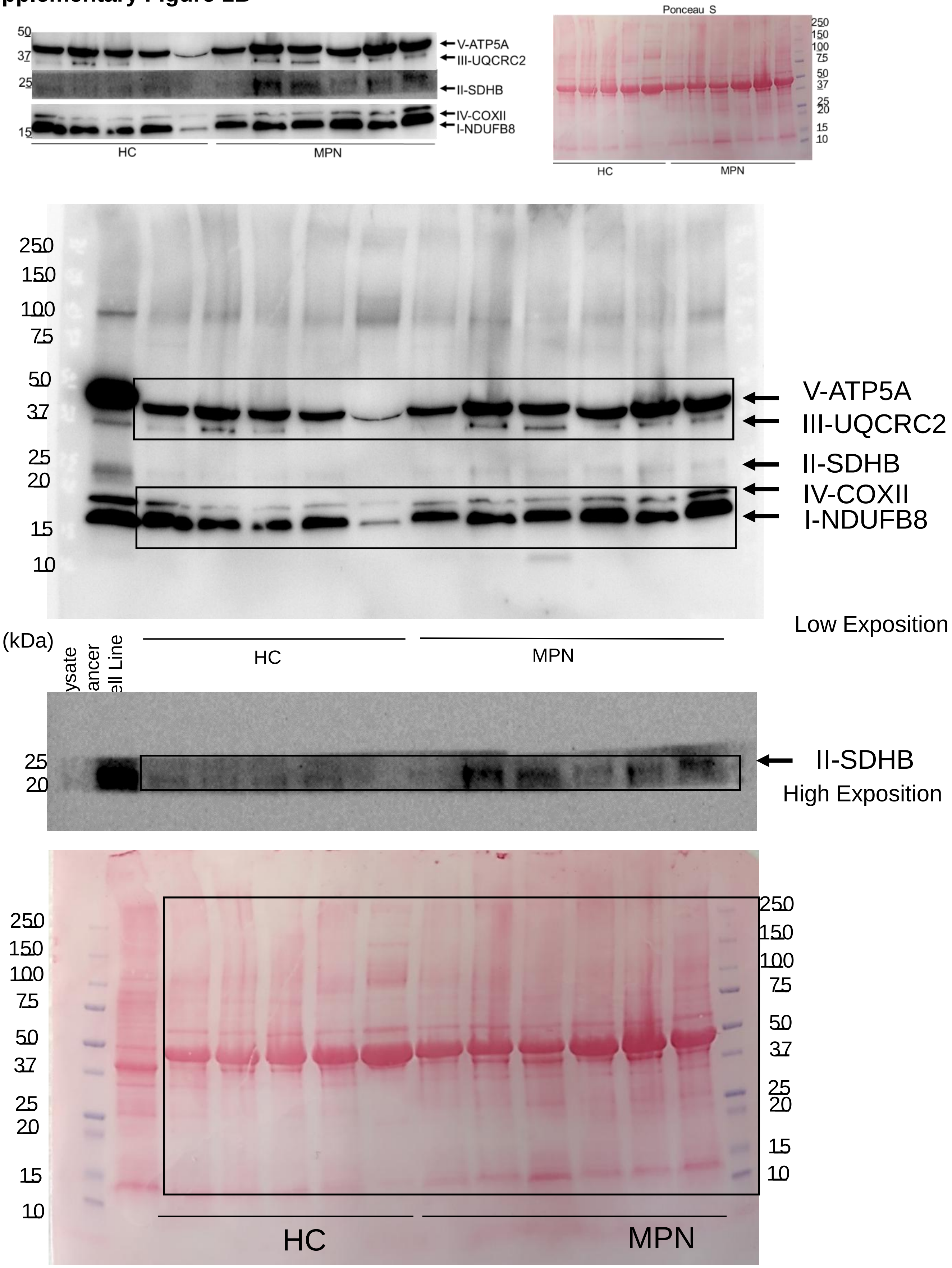

Supplementary Figure 2A

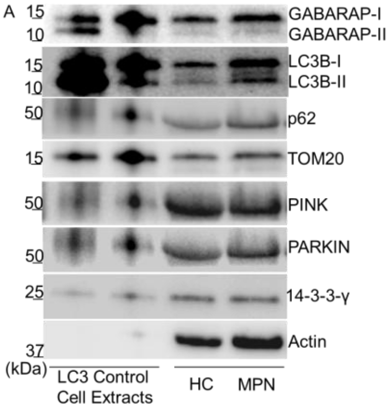

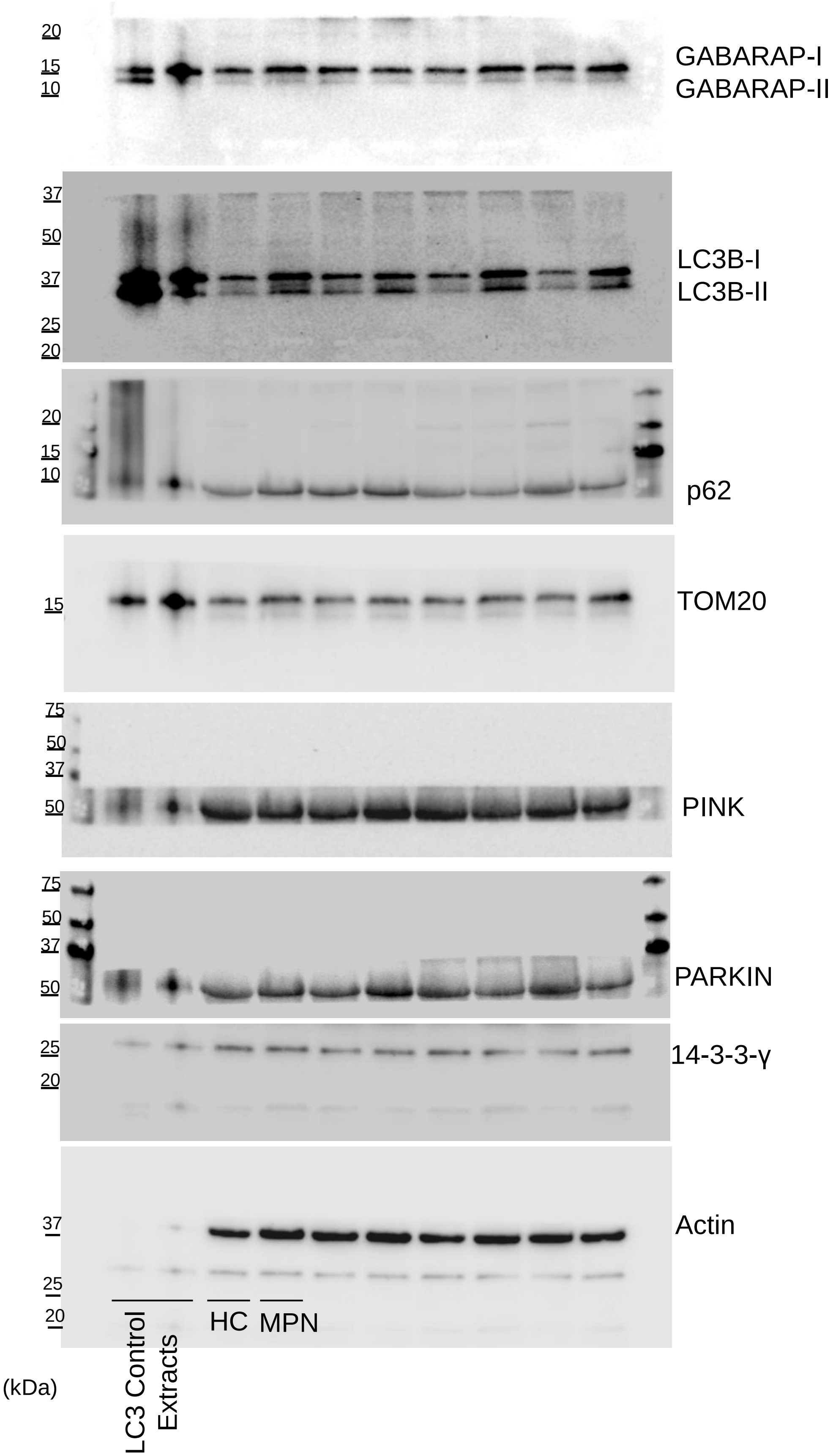

Supplementary Figure 2A

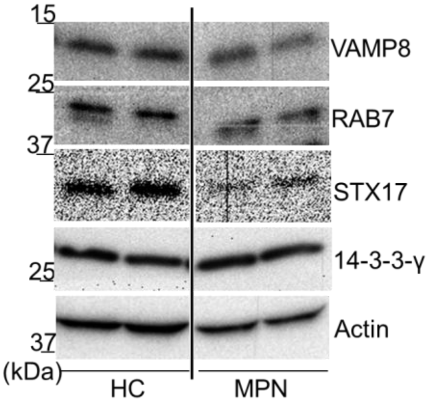

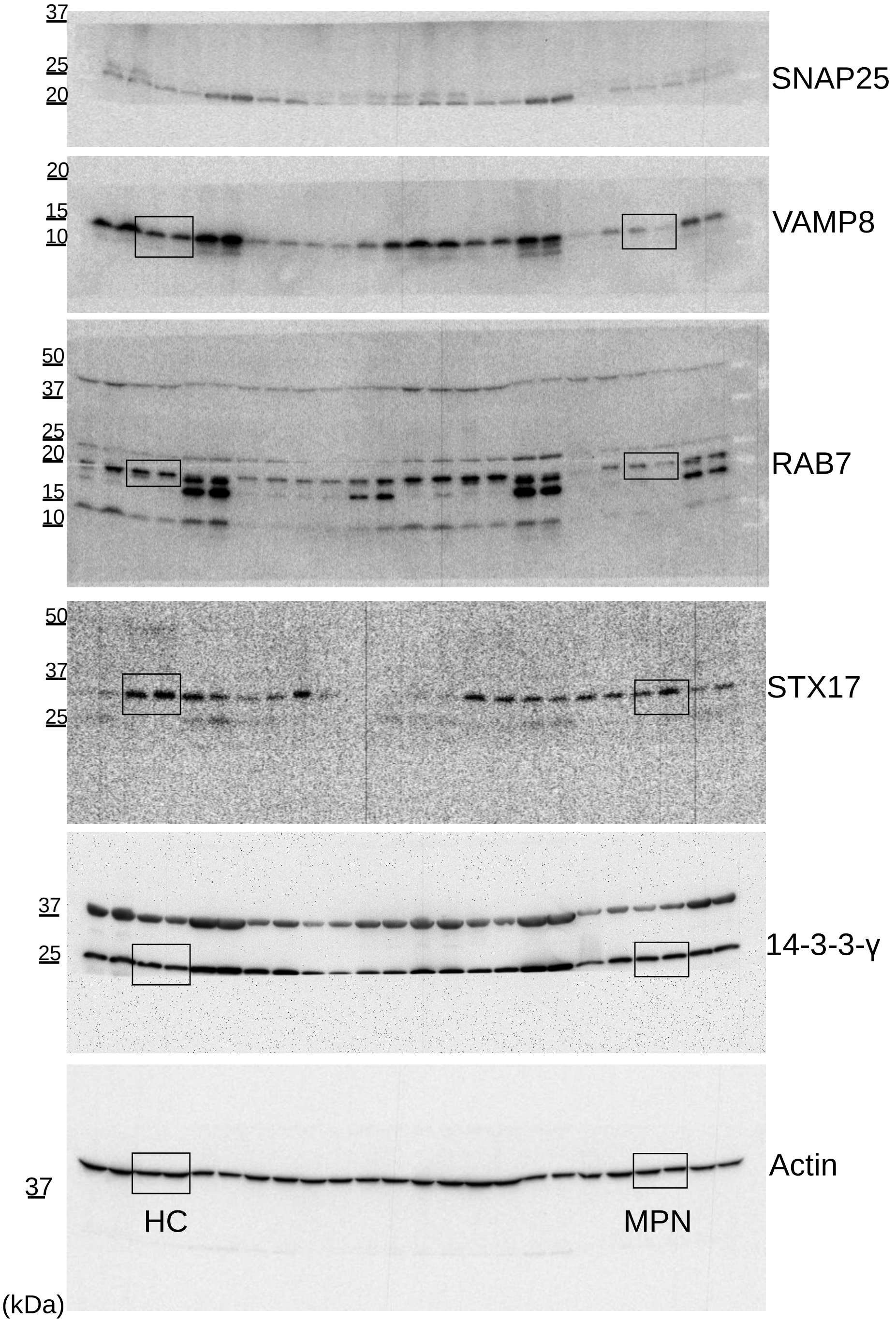

Supplementary Figure 2A

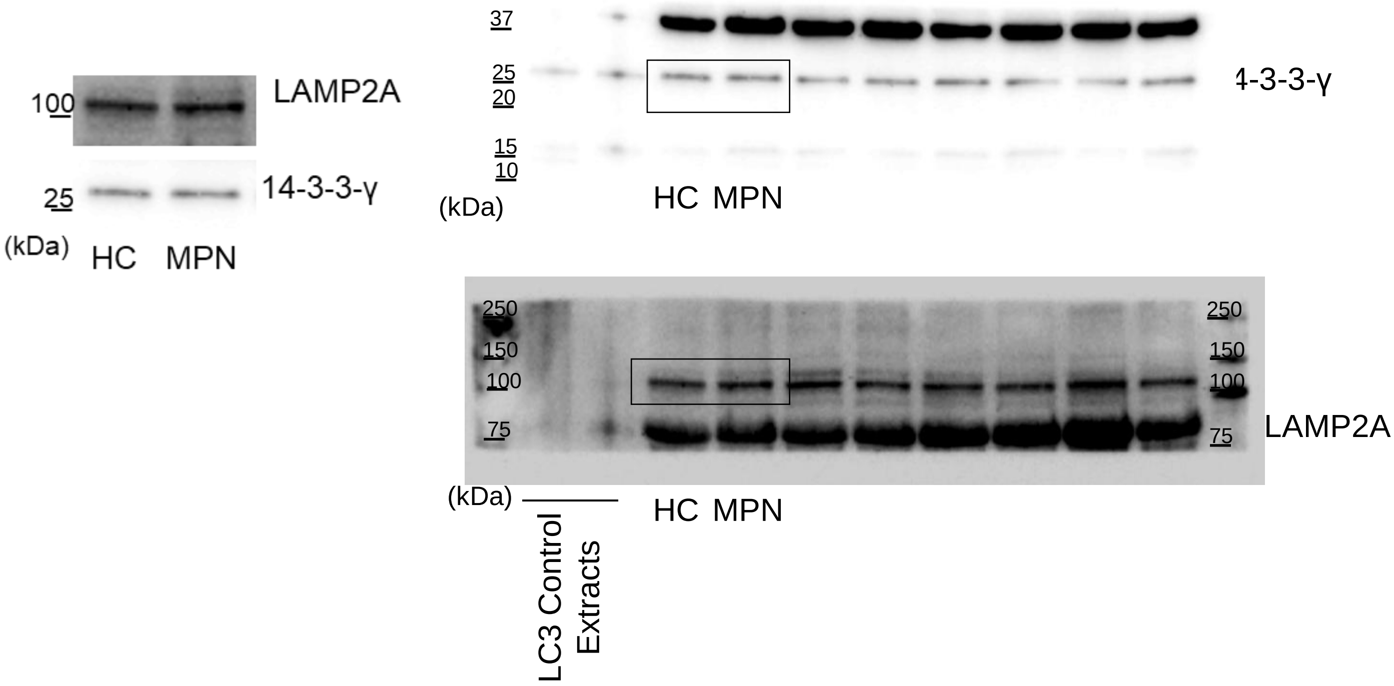

Supplementary Figure 2C

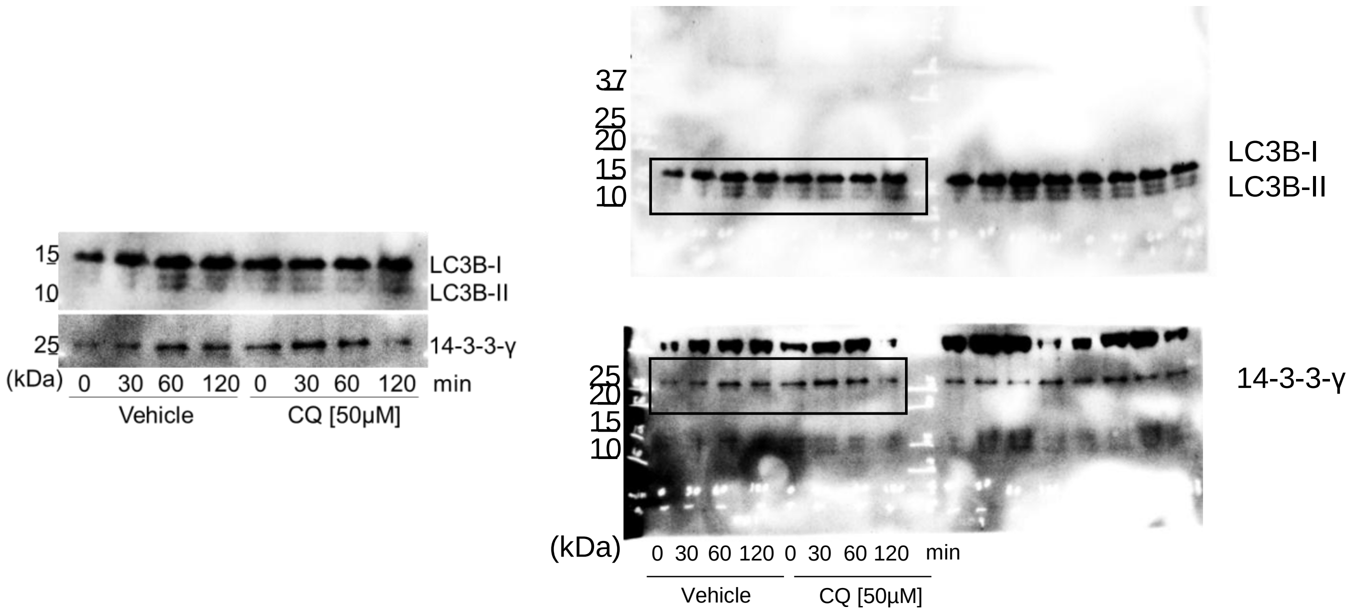

Supplementary Figure 2D

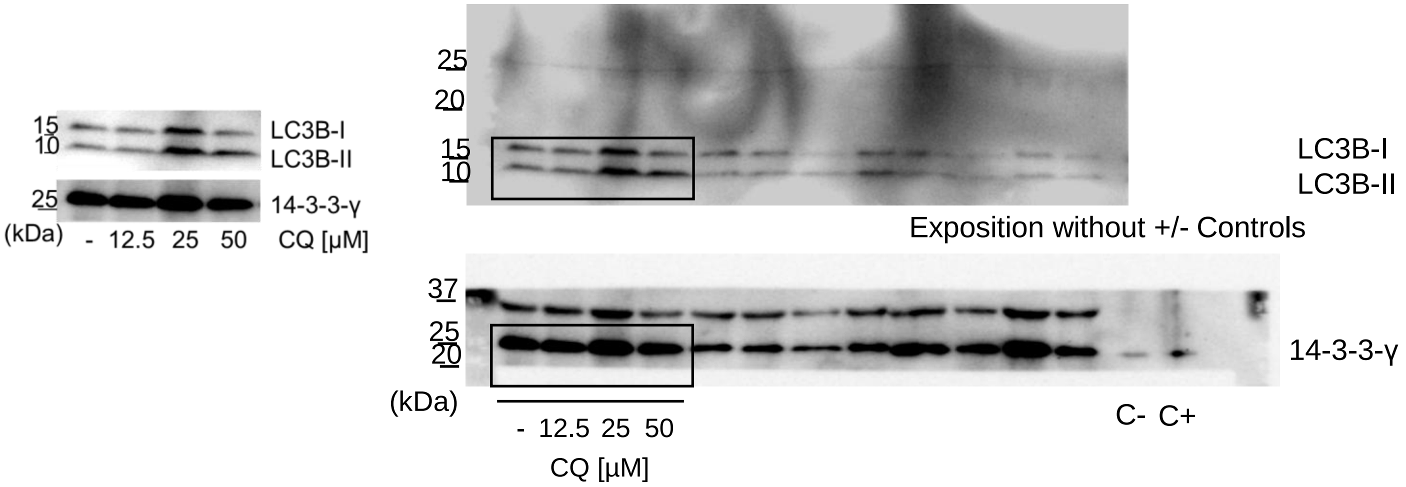

Supplementary Figure 3A

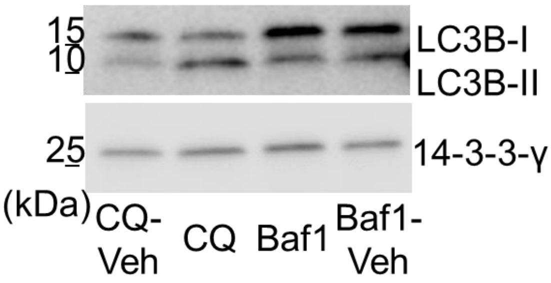

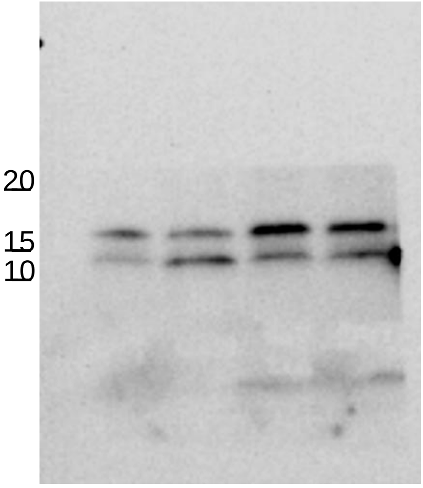

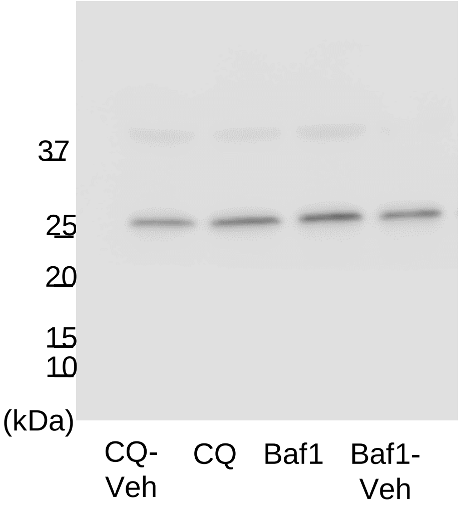

Supplementary Figure 4A

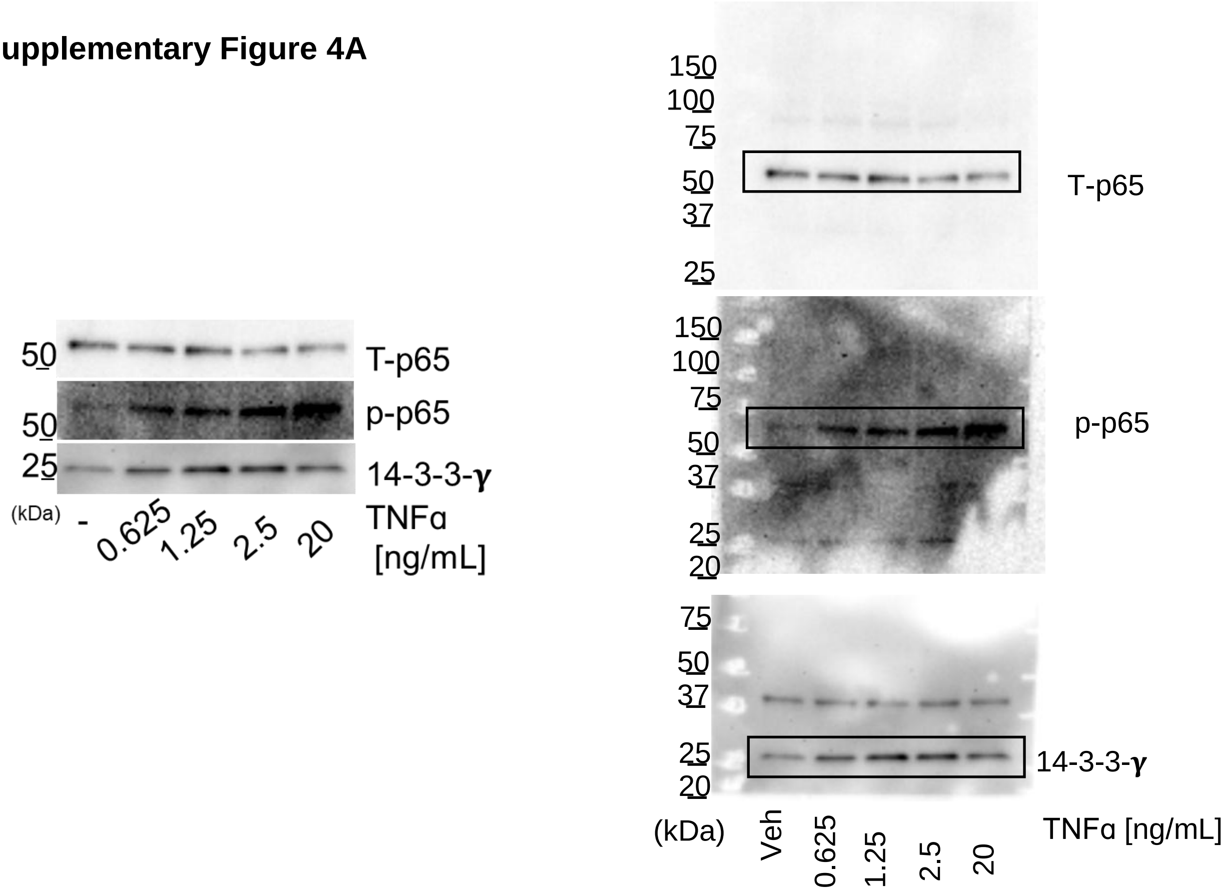

Supplementary Figure 4C

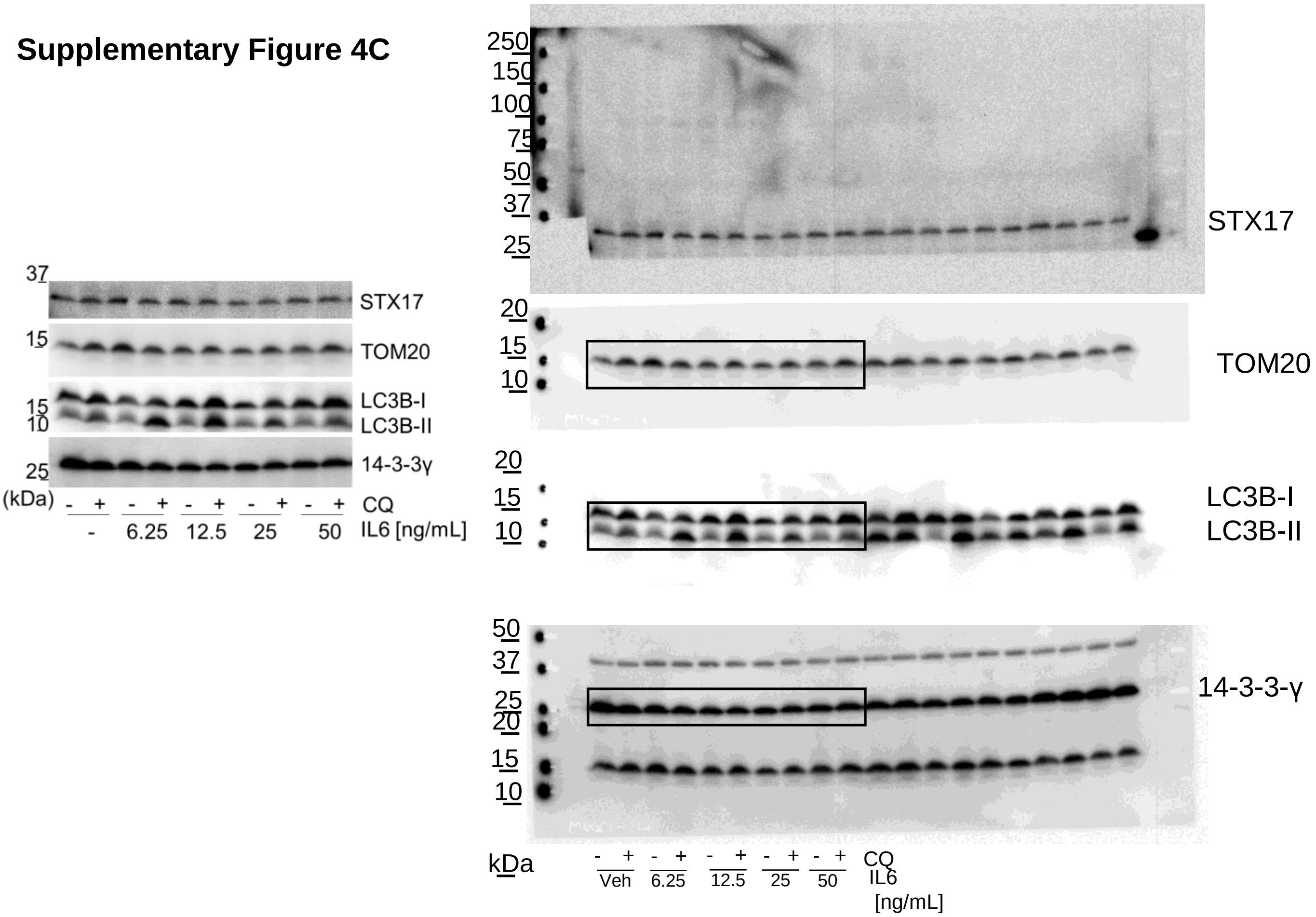

Supplementary Figure 4C

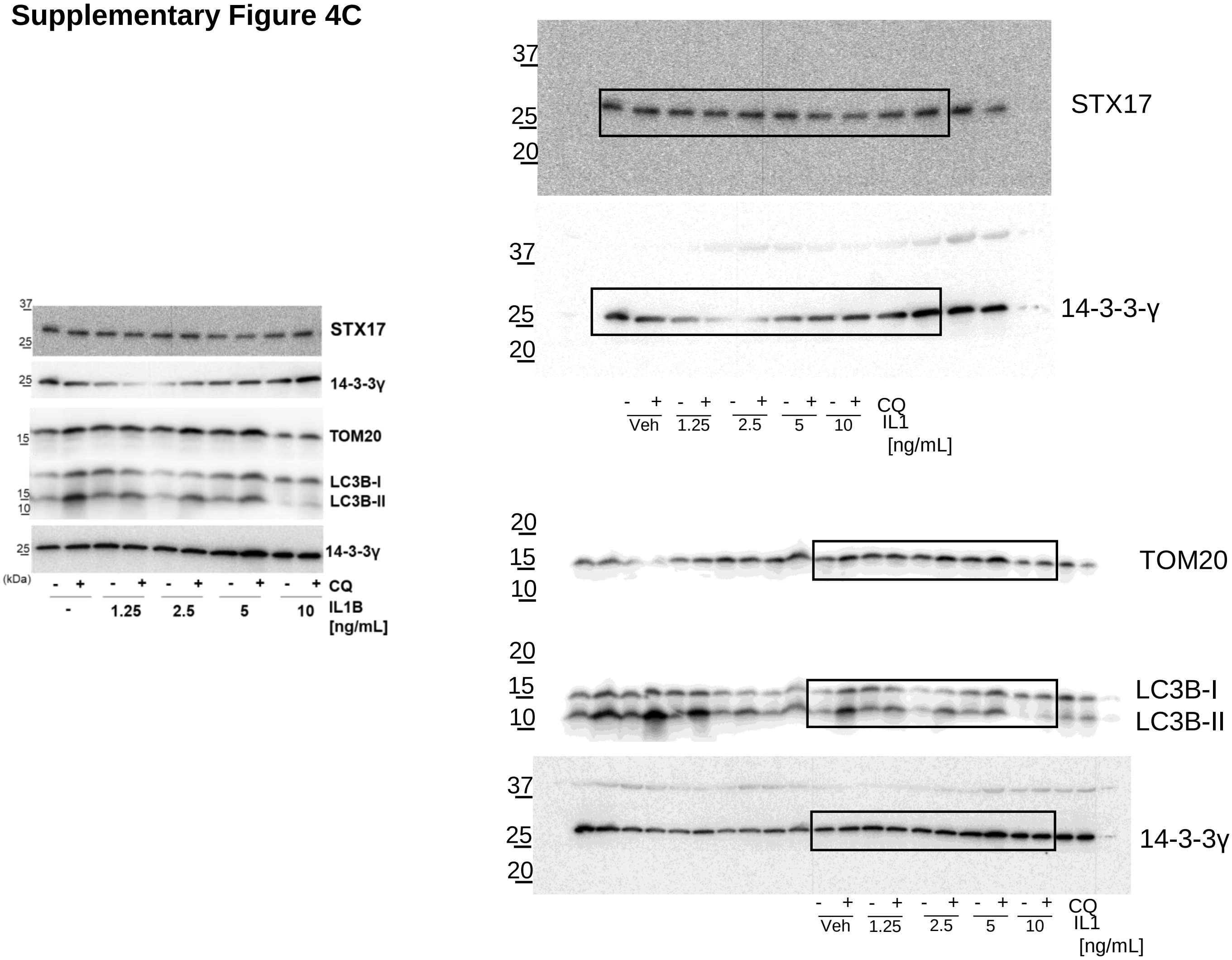

Supplementary Figure 4E

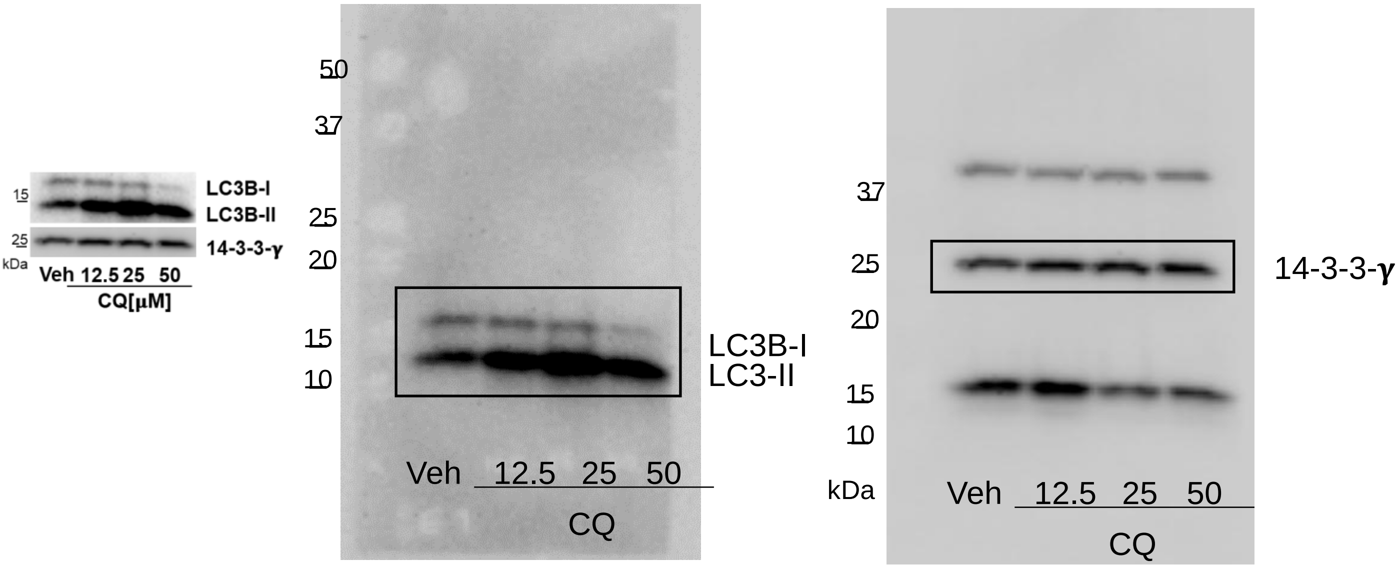

Supplementary Figure 5I

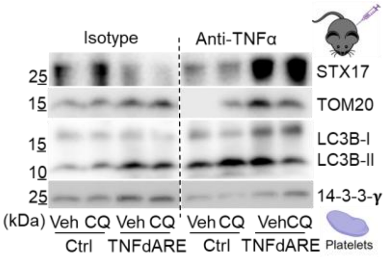

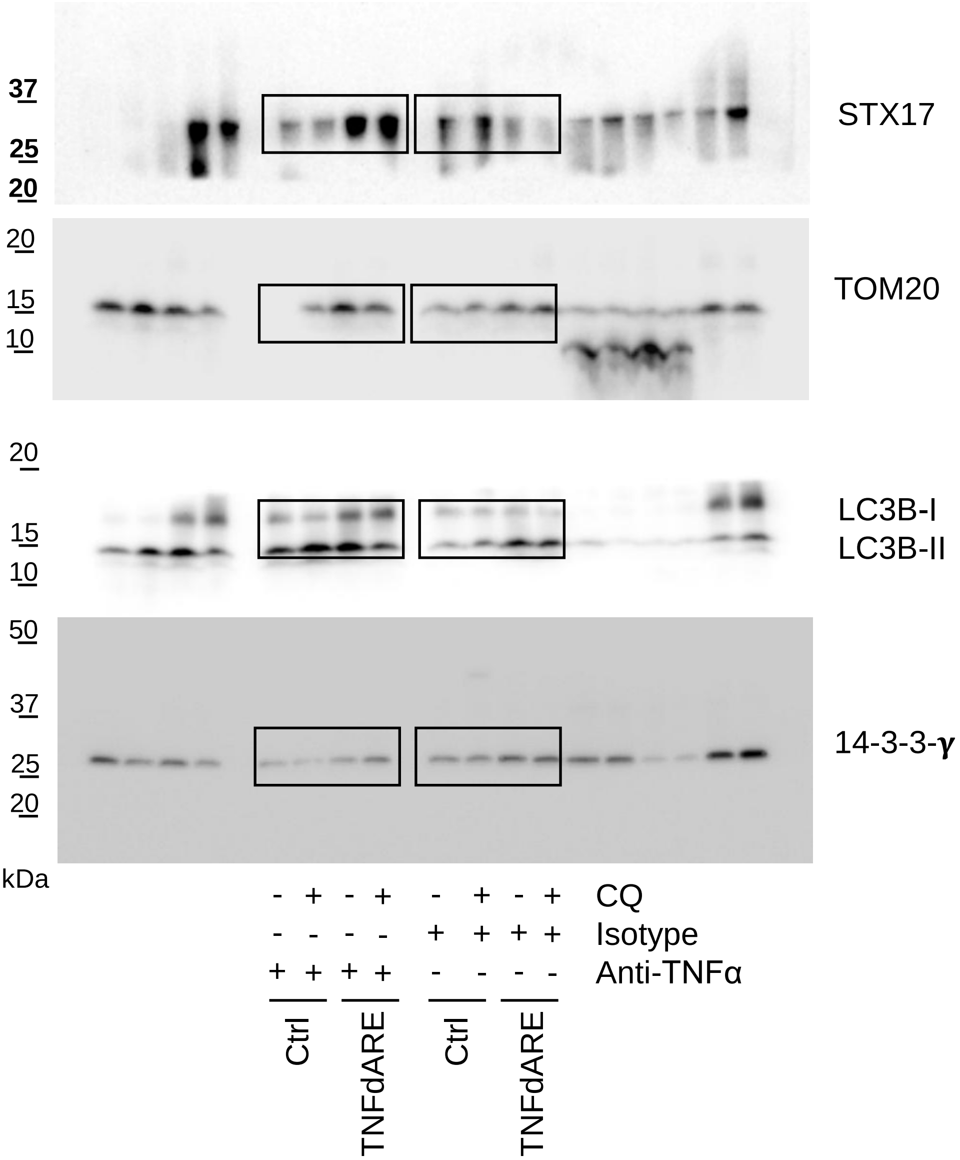

Supplement: Unedited blot and gel images [file jci-135-186065-s026.pdf]
